# Identification of hub genes related to the innate immune response activated during spinal cord injury

**DOI:** 10.1002/2211-5463.13472

**Published:** 2022-09-01

**Authors:** Jianfeng Li, Xizhe Liu, Huachuan Wu, Peng Guo, Baoliang Li, Jianmin Wang, Wei Tian, Dafu Chen, Manman Gao, Zhiyu Zhou, Shaoyu Liu

**Affiliations:** ^1^ Innovation Platform of Regeneration and Repair of Spinal Cord and Nerve Injury, Department of Orthopedic Surgery, The Seventh Affiliated Hospital Sun Yat‐sen University Shenzhen China; ^2^ Guangdong Provincial Key Laboratory of Orthopedics and Traumatology, Orthopedic Research Institute/Department of Spinal Surgery The First Affiliated Hospital of Sun Yat‐sen University Guangzhou China; ^3^ Laboratory of Bone Tissue Engineering, Beijing Laboratory of Biomedical Materials, Beijing Research Institute of Orthopedics and Traumatology Beijing Jishuitan Hospital China; ^4^ Department of Sport Medicine, Institute of Translational Medicine The First Affiliated Hospital of Shenzhen University, Shenzhen Second People's Hospital China; ^5^ Guangdong Key Laboratory for Biomedical Measurements and Ultrasound Imaging, School of Biomedical Engineering Shenzhen University Health Science Center China

**Keywords:** bioinformatics analysis, hub gene, immunity therapy, innate immune infiltration, spinal cord injury, temporal expression

## Abstract

Spinal cord injury (SCI) often leads to sensory and motor dysfunction. Two major factors that hinder spinal cord repair are local inflammation and glial scar formation after SCI, and thus appropriate immunotherapy may alleviate damage. To characterize changes in gene expression that occur during SCI and thereby identify putative targets for immunotherapy, here we analyzed the dataset GSE5296 (containing one control group and six SCI groups at different timepoints) to identify differentially‐expressed genes. Functional enrichment analysis was performed and a protein–protein interaction network was created to identify possible hub genes. Finally, we performed quantitative PCR to verify changes in gene expression. The CIBERSORT algorithm was used to analyze innate immune cell infiltration patterns. The dataset GSE162610 (containing one control group and three SCI groups at different timepoints) was analyzed to evaluate innate immune cell infiltration at the single‐cell level. The dataset GSE151371 (containing one control group [*n* = 10] and an SCI group [*n* = 38]) was used to detect the expression of hub genes in the blood from SCI patients. Differentially‐expressed innate immune‐related genes at each timepoint were identified, and the functions and related signaling pathways of these genes were examined. Six hub genes were identified and verified. We then analyzed the expression characteristics of these hub genes and characteristics of innate immune infiltration in SCI; finally, we examined ligand expression in the context of the CCL signaling pathway and COMPLEMENT signaling pathway networks. This study reveals the characteristics of innate immune cell infiltration and temporal expression patterns of hub genes, and may aid in the development of immunotherapies for SCI.

## Graphical abstract

Here we report the characteristics of innate immune cell infiltration in spinal cord injury (SCI) and temporal expression of innate immune‐related hub genes. Additionally, we report the involvement of certain signaling pathways for cell‐to‐cell communication and associated receptor‐ligand combinations in SCI. These findings may help efforts in the development of effective and timely therapies for SCI.

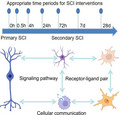

**Abbreviations**
*C3*
: complement 3
*Ccl2*
: C‐C motif chemokine ligand 2
*Ccr2*
: C‐C motif chemokine receptor 2
*CXCL10*
: C‐X‐C motif chemokine ligand 10
*Cxcl10*
: C‐X‐C motif chemokine ligand 10
DCs: dendritic cells
GEO: Gene Expression Omnibus
GO: Gene Ontology
*IL‐1R*
: interleukin 1 receptor type 1
*ITGAM*
: integrin subunit alpha M
*Itgam*
: integrin subunit alpha M
*Itgb2*
: integrin subunit beta 2
KEGG: Kyoto Encyclopedia of Genes and Genomes
*MAPK14*
: mitogen‐activated protein kinase 14
*Mapk14*
: mitogen‐activated protein kinase 14
*Mapk8*
: mitogen‐activated protein kinase 8
*MYD88*
: myeloid differentiation primary response 88
*Myd88*
: myeloid differentiation primary response 88
NCBI: National Center for Biotechnology Information
*NF‐κB*
: nuclear factor of kappa light polypeptide gene enhancer in B cells
NK: natural killer
OPC: oligodendrocyte progenitor cell
PCs: principal components
PPIN: protein–protein interaction network
qPCR: quantitative polymerase chain reaction
SCI: spinal cord injury
SEM: standard error of the mean
*STAT1*
: signal transducer and activator of transcription 1
*Stat1*
: signal transducer and activator of transcription 1
*STAT3*
: signal transducer and activator of transcription 3
*Stat3*
: signal transducer and activator of transcription 3
STRING: Search Tool for Retrieval of Interacting Genes/Proteins
*TLR*
: Toll‐like receptor
*TLR2*
: Toll‐like receptor 2
*Tlr2*
: Toll‐like receptor 2
*TLR4*
: Toll‐like receptor 4
UMAP: uniform manifold approximation and projection

Spinal cord injury (SCI) often leads to sensory and motor dysfunction. SCI is accompanied by a series of complications and psychological problems, and places a heavy burden on individuals, families, and society [1]. Reportedly, the global incidence of SCI is about 23 per million and mainly involves adult men, most of whom are victims of motor vehicle accidents and falls [2, 3, 4]. Despite great progress made in understanding the pathophysiological changes and regeneration mechanisms of SCI, the cure for SCI remains a medical problem. Two major factors that hinder spinal cord repair are local inflammation and glial scar formation after SCI. The inflammatory response in SCI can not only aggravate SCI, but also promote its repair. Human SCI is defined as a primary injury and can be divided into the immediate phase (< 2 h), acute phase (< 48 h), subacute phase (48 h to 14 days), intermediate phase (14 days to 6 months), and chronic phase (> 6 months) [3, 5]. Reportedly, the levels of proinflammatory cytokines increase in the spinal cord a few minutes after SCI in mice [6]. Inflammatory cells in blood vessels, such as neutrophils and macrophages, infiltrate the SCI area due to local vascular damage and cause inflammation driven by inflammatory chemokines [7, 8, 9]. These phenomena are inherent in this process, and the expressions of some innate immune‐related genes also change [10, 11, 12].

Based on the above findings, we obtained differentially‐expressed immune‐related genes in SCI tissues of mice through dataset analysis, analyzed the changes in related functions and signaling pathways, and identified hub genes and their temporal expression characteristics. We also performed SCI modeling of mice and quantitative PCR (qPCR) to verify the temporal expression changes of hub genes. After that, the communication relationship between some cells, as well as the temporal expression characteristics of hub genes in various cells, were obtained at the single‐cell level through dataset analysis. The expressions of hub genes in the blood of human SCI were explored through dataset analysis. The temporal characteristics of immune infiltration in SCI were also explored. The workflow of this study is shown in Fig. 1.

**Fig. 1:**
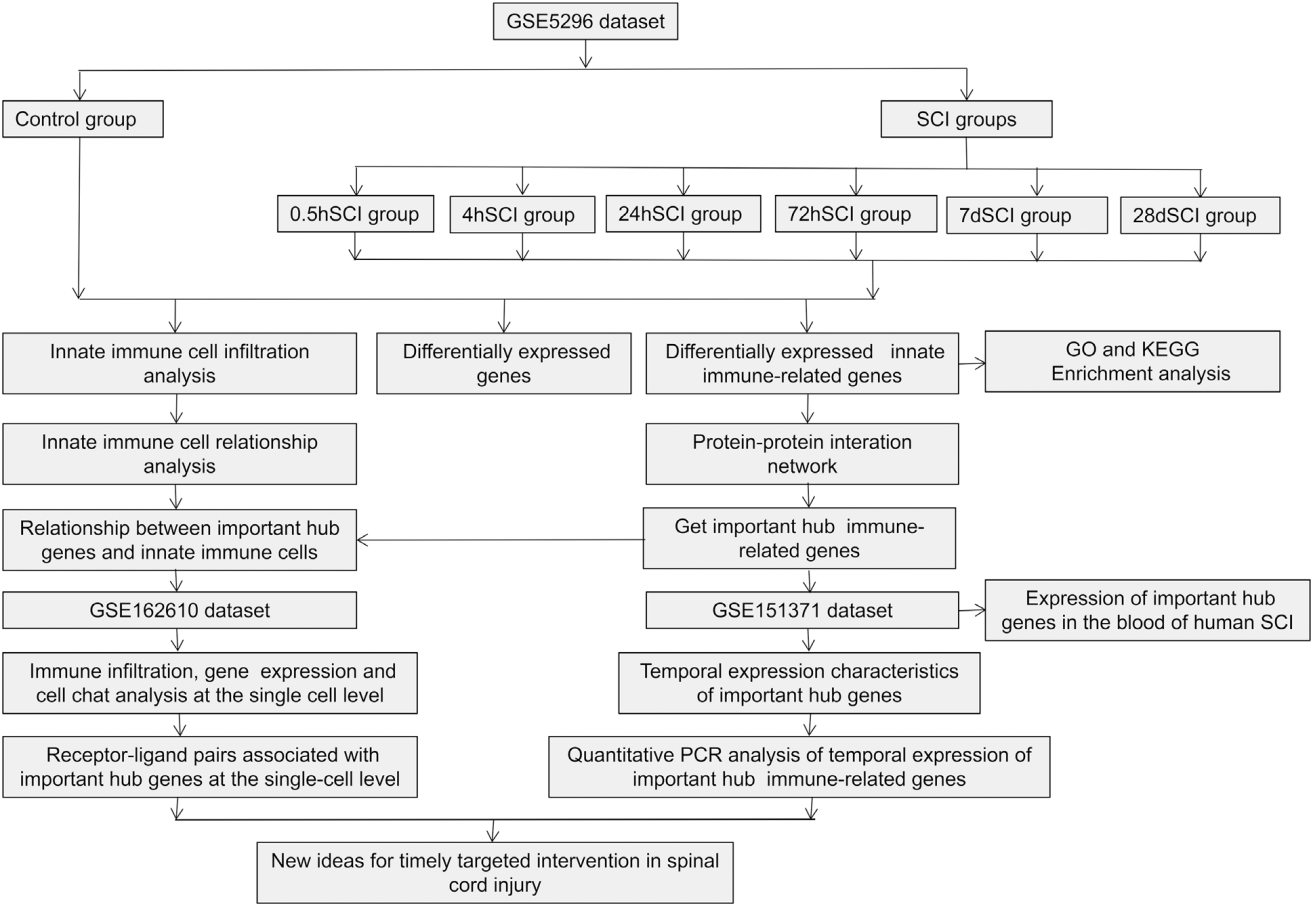
Workflow of this study.

## Materials and methods

### Animals in our validation experiments

Twenty‐one female C57BL/6 mice weighing 20–22 g, 6 weeks old, were purchased from Baishitong Biological Technology (Zhuhai, China). All animals were kept in the Laboratory Animal Center of Sun Yat‐Sen University, with the animal use permit number SYXK (Guangdong) 2015‐0107. The animal experiments were approved by the Institutional Animal Care and Use Committee of Sun Yat‐Sen University on April 19, 2021 (approval number: SYSU‐IACUC‐2021‐000196). The address is Laboratory Animal Center, Sun Yat‐Sen University, No. 74, Zhongshan Road II, Guangzhou, 510080, P.R. China. All mice were kept in an environment with controlled temperature and humidity, with a 12‐h light/dark cycle, fed on time, watered, and the litter changed. All breeding and experimental operations followed relevant guidelines provided by the Ethics Committee. Then we performed some experiments using these mice under isoflurane inhalation anesthesia, and constructed a moderate spinal cord contusion model at the T8 spinal segment according to Allen's blow method in the SCI group [13]. The control samples only received laminectomy and the T8 spinal segment was not injured. RNAs from the injured spinal cord tissues were extracted for verification of expression levels of hub genes.

### Dataset source and basic information

First, datasets GSE5296 microarray, GSE162610, and GSE151371 were downloaded from the Gene Expression Omnibus (GEO, https://www.ncbi.nlm.nih.gov/gds), and their platforms were GPL1261 (Affymetrix Mouse Genome 430 2.0 Array, San Francisco, CA, USA)), GPL19057 (Illumina NextSeq 500, San Diego, CA, USA) and GPL20301 (Illumina HiSeq 4000, San Diego, CA, USA), respectively. In GSE5296, C57BL/6 mice were used to construct SCI models under isoflurane anesthesia based on Allen's method [13]. Six SCI groups were given moderate injury at the T8 spinal segment, which was not injured in the control group. Then the injured segments, about 4 mm of spinal cord tissues, were obtained at certain timepoints for sequencing. Data were collected from the control group (*n* = 2) and six SCI groups at different timepoints (0.5, 4, 24, and 72 h and 7 and 28 days; *n* = 3/timepoint). Then GSE5296 was normalized using the function normalizeBetweenArrays in the package bioconductor limma, and the correction method was “quantile” [14]. In GSE162610, C57BL/6 female mice of 8–10 weeks were also used to construct a moderate spinal cord contusion model at the T8 spinal segment according to Allen's blow method in the SCI group [13], and the T8 spinal segment was not injured in the control group. This dataset included one control group (*n* = 5) and three SCI groups at different timepoints (1, 3, and 7 days, from 5, 3, and 3 mice, respectively). This dataset used the canonical correlation analysis algorithm in the package seurat r to correct the batch effect. GSE151371 was normalized before downloading and included one control group (blood from 10 health people) and one SCI group (blood from 38 SCI patients). Blood of humans was collected at 30.3 ± 18.9 h after SCI, but there were no special requirements for the time to collect blood from healthy volunteers in the control group. Lastly, the list of innate immunity‐related genes was downloaded from the Innate DB database (https://www.innatedb.com) to obtain the expression matrix of these genes using the package “limma.”

### Identification of differentially‐expressed genes and differentially‐expressed innate immune‐related genes

The differentially‐expressed genes and innate immune‐related genes in the control group and experimental groups were identified using the package bioconductor limma [15]. These genes were screened out using adjusted *P* < 0.05 as the significance threshold. The results were visualized using the packages “limma” and “pheatmap.”

### Gene Oncology and KEGG enrichment analysis of differentially‐expressed innate immune‐related genes

Gene Ontology (GO) and Kyoto Encyclopedia of Genes and Genomes (KEGG) pathway analyses of differentially‐expressed innate immune‐related genes and the selected innate immune‐related hub genes were performed with the busing packages “org.mm.eg.db,” “clusterprofiler,” “ggplot2,” and “enrichplot” [16]. The cutoff criterion was *P* < 0.05.

### Protein–protein interaction network and hub genes

Protein–protein interaction network (PPIN) analysis of innate immune‐related genes was performed using the Search Tool for Retrieval of Interacting Genes/Proteins (STRING) (https://www.string‐db.org/), and the minimum required interaction score was set to be 0.400. The nodes that were disconnected in the network were hidden in the PPIN. Then four genes of the top four degrees at the timepoint 0.5 h and 10 genes of the top 10 degrees at the timepoints 4, 24, and 72 h and 7 and 28 days were visualized using bar plots. Among the genes screened above, those that appeared at least at three timepoints were identified as hub genes. The genes that appeared at least at three timepoints were identified as hub genes. Then dynamic changes in the hub genes at different timepoints were visualized using R packages “ggplot” and “reshape” (Vienna, Austria). Then National Center for Biotechnology Information (NCBI) (https://www.ncbi.nlm.nih.gov/gene/) and UniProt (https://www.uniprot.org/) were searched to find the full names and functions of the hub genes.

### Quantitative PCR of gene expression

We constructed a similar mouse SCI model according to GSE5296 by using Allen's method. There were three mice in the blank group and the injury group at each timepoint. All animal operations were performed under general anesthesia after inhalation with isopentane (RWD Life Science, Shenzhen, China). Then spinal cord tissues were collected from the injured areas, and RNA was extracted from these tissues using an RNAeasy animal RNA extraction kit (Beyotime, Shanghai, China). The RNA was reverse‐transcribed into DNA using PrimeScript RT Master Mix (TAKARA, Dalian, China). Then the expression levels of hub genes at various timepoints after SCI were verified through qPCR. PowerUp SYBR reagents (Thermo Fisher Scientific, Waltham, MA, USA) and a qPCR platform (Bio‐Rad, Hercules, CA, USA) were used. The primer sequences used for qPCR were listed in Table 1.

**Table 1:** Primers used for RT‐qPCR.

Gene	Sequences (5′–3′)
*Gapdh*	Forward TGGAATCCTGTGGCATCCATGAAAC
	Reverse TAAAACGCAGCTCAGTAACAGTCCG
*Ccl2*	Forward TAAAAACCTGGATCGGAACCAAA
	Reverse GCATTAGCTTCAGATTTACGGGT
*MyD88*	Forward GACCGTGAGGATATACTGAAGGA
	Reverse GGCCACCTGTAAAGGCTTCTC
*Stat3*	Forward CACCTTGGATTGAGAGTCAAGAC
	Reverse AGGAATCGGCTATATTGCTGGT
*Cxcl10*	Forward CCAAGTGCTGCCGTCATTTTC
	Reverse GGCTCGCAGGGATGATTTCAA
*Tlr2*	Forward primer TCTAAAGTCGATCCGCGACAT
	Reverse primer CTACGGGCAGTGGTGAAAACT
*Mapk8*	Forward primer GTGGAATCAAGCACCTTCACT
	Reverse primer TCCTCGCCAGTCCAAAATCAA
*Itgam*	Forward primer GGCTCCGGTAGCATCAACAA
	Reverse primer ATCTTGGGCTAGGGTTTCTCT
*Mapk14*	Forward primer ACCTAGCTGTGAACGAAGACT
	Reverse primer GTAGCCACGTAGCCTGTCATC
*Stat1*	Forward primer TCACAGTGGTTCGAGCTTCAG
	Reverse primer CGAGACATCATAGGCAGCGTG

### Assessment of innate immune cell infiltration

The mouse reference gene file to define 11 subgroups of innate immune cells was downloaded [17]. The CIBERSORT algorithm was used to evaluate the infiltration of innate immune cells from the peripheral blood to injured spinal cord tissues [18]. R packages including “limma,” “ggplot2,” “ggpubr,” and “ggextra” and Spearman correlation tests were used to evaluate the relationship between the expressions of hub genes and the proportion of innate immune cells. Then *P* < 0.05 was set as the cutoff criterion to judge the correlations between them.

### Single‐cell sequencing dataset analysis of SCI innate immunity

GSE162610 was analyzed using some R packages, including “seurat,” “ggplot2,” “cowplot,” “matrix,” “dplyr,” “ggsci,” “cellchat,” and “singler.” We filtered the data to retain cells with more than 400 genes and 1000 transcripts to exclude genes expressed in fewer than 10 cells. Cells with more than 5% of mitochondrial genes were also filtered out. The Uniform Manifold Approximation and Projection (UMAP) and 16 significant principal components (PCs) used in dimensionality reduction and clustering. Annotation of cell types was based on the package “singler” [19], database CellMarker (http://bio‐bigdata.hrbmu.edu.cn/CellMarker/), and related articles [20, 21, 22]. All immune cell populations were extracted, reclustered and annotated according to GO function analysis and definition methods [20]. Then the proportion of cell subpopulations was calculated, and the expression levels of some hub genes in various cell subpopulations were displayed.

### Analysis of expressions of hub genes in the blood of SCI patients

The R package “ggpubr” was used to detect the expression statuses of hub genes in GSE151371, and human SCI blood transcriptome sequencing data. Then *P* < 0.05 was used as the significance threshold to screen differentially‐expressed genes.

### Statistical analysis

The qPCR data are expressed as mean ± SEM from at least three independent experiments. Student's *t*‐test was used for two‐group comparison of qPCR data. *P* < 0.05 was considered significant.

## Results

### Identification of differentially‐expressed genes and innate immune‐related genes

**Fig. 2:**
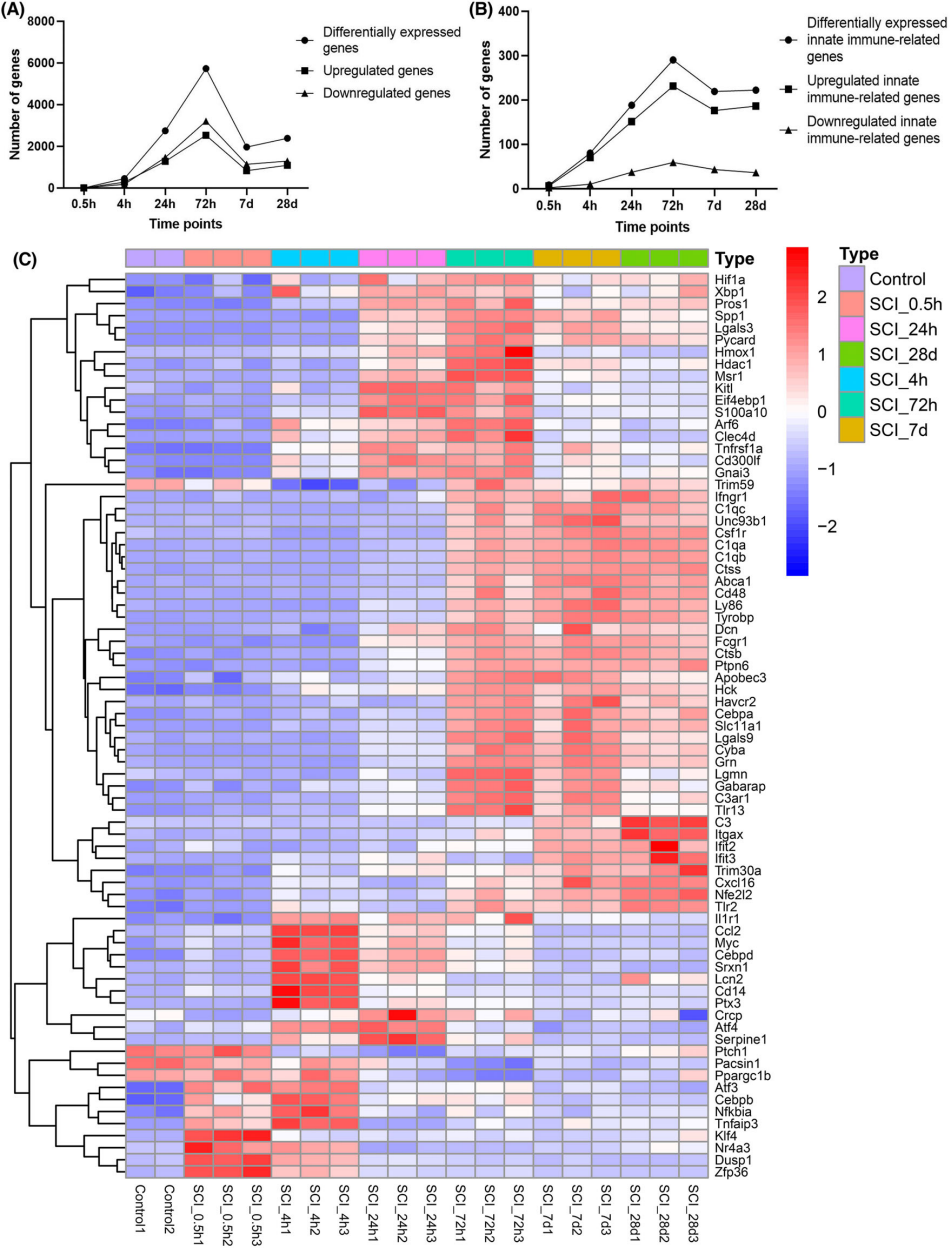
Differential expression of genes in SCI. (A) The number of genes in the SCI that are differently expressed throughout time. The abscissa shows each timepoint following SCI, while the ordinate reflects the number of genes. (B) Temporal evolution of the number of genes involved in innate immunity that are differently expressed in SCI. The abscissa indicates each timepoint following SCI, while the ordinate reflects the quantity of innate immunity‐related genes. (C) Heatmap of several SCI genes associated with innate immunity that exhibit differential expression. Each column represents an SCI sample, and each row is a gene associated with innate immunity that is differentially expressed. Statistical significance was defined as an adjustment *P* value < 0.5. d, day; h, hour.

Through dataset analysis, differentially‐expressed genes and innate immune‐related genes were identified. The number of differentially‐expressed genes peaked at 72 h after SCI, but the number of downregulated genes from 24 h to 28 days after SCI was larger than that of upregulated genes (Fig. 2A). The number of differentially‐expressed immune‐related genes maximized at 72 h, and maintained a high level until 28 days, but the number of upregulated genes was far greater than that of downregulated genes (Fig. 2B). Then, eight differentially‐expressed innate immune‐related genes at 0.5 h after SCI and 20 differentially‐expressed innate immune‐related genes with the lowest adjusted *P* value at other timepoints were visualized using heatmaps (Fig. 2C). All differentially‐expressed innate immune‐related genes are listed in Table S1.

### GO and KEGG enrichment analysis of differentially‐expressed innate immune‐related genes at each timepoint after SCI

κGO enrichment analysis showed the functions mainly included positive regulation of response to external stimulus, regulation of immune cell chemotaxis and migration, immune cell activation, regulation of DNA‐binding transcription factor, and negative regulation of angiogenesis. Ten GO terms with the lowest *P* value at each timepoint and related innate immune‐related genes are listed in Table S2. KEGG enrichment analysis demonstrated the complement and coagulation cascades. The *IL‐17* signaling pathway changed at 0.5 h after SCI. Some pathways, such as the *TNF*, *NF‐κB*, *IL‐17*, Toll‐like receptor, and NOD‐like receptor signaling pathways changed in the 4th hour to 28 days. Ten KEGG pathway terms of upregulated or downregulated immune‐related genes with the lowest *P* values at each timepoint are listed in Table S3.

### Construction of PPIN and screening of hub genes by dataset analysis

**Fig. 3:**
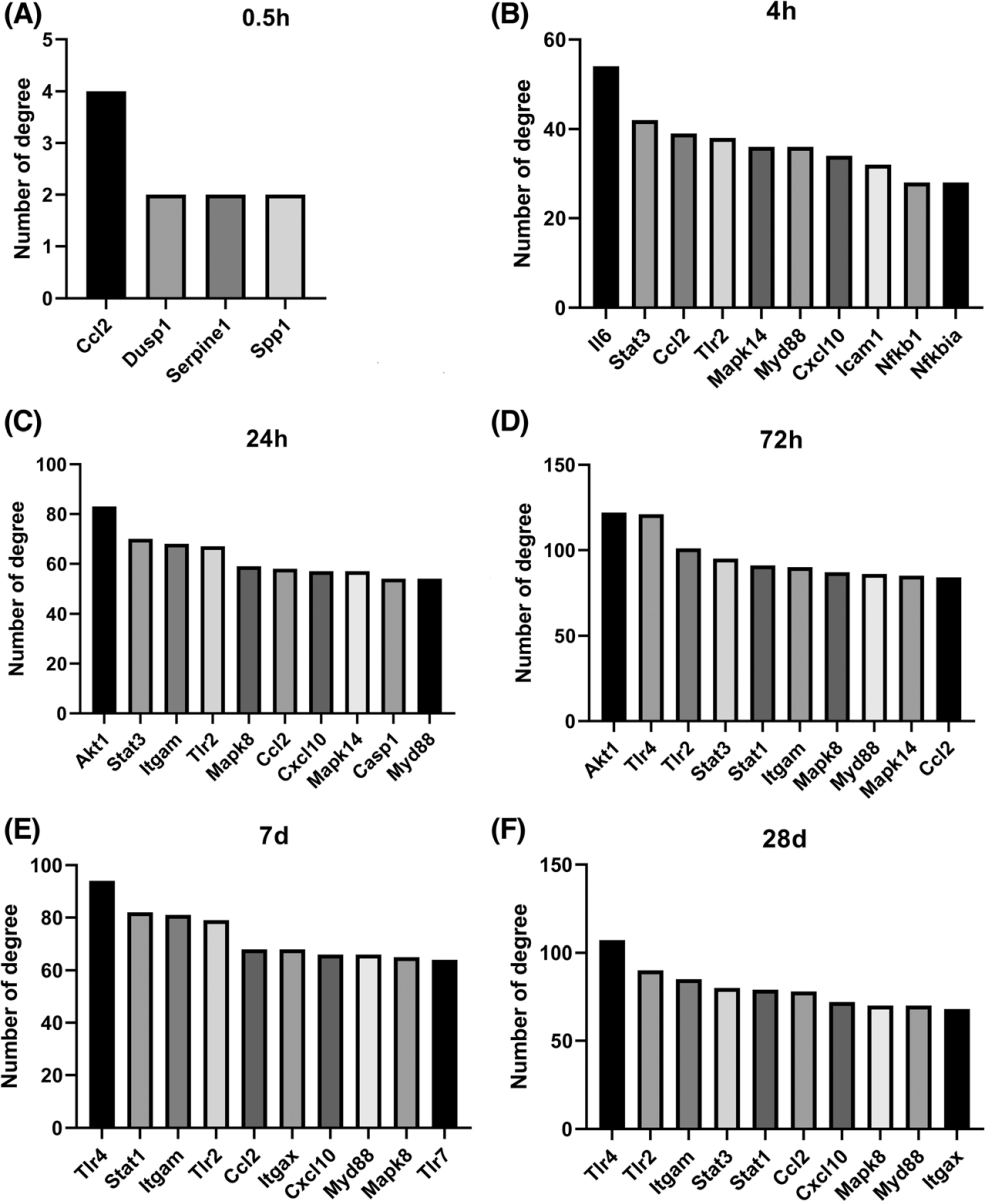
PPIN and hub gene identification. (A) Top 4 genes in the PPIN were screened based on their connectivity degree at 0.5 h after SCI. (B) Top 10 genes in the PPIN were screened based on their connectivity degree at 4 h after SCI. (C) Top 10 genes in the PPIN were screened based on their connectivity degree at 24 h after SCI. (D) Top 10 genes in the PPIN were screened based on their connectivity degree at 72 h after SCI. (E) Top 10 genes in the PPIN were screened based on their connectivity degree at 7 d after SCI. (F) Top 10 genes in the PPIN were screened based on their connectivity degree at 28 d after SCI. d, day; h, hour.

The PPIN at each timepoint was made. Four genes at 0.5 h and 10 genes at other timepoints were the top degrees (Fig. 3). Then nine hub genes that appeared in at least three timepoints were identified, including C‐C motif chemokine ligand 2 (*Ccl2*), signal transducer and activator of transcription 3 (*Stat3*), mitogen‐activated protein kinase 14 (*Mapk14*), signal transducer and activator of transcription 1 (*Stat1*), Toll‐like receptor 2 (*Tlr2*), C‐X‐C motif chemokine ligand 10 (*Cxcl10*), myeloid differentiation primary response 88 (*Myd88*), integrin subunit alpha M (*Itgam*) and mitogen‐activated protein kinase 8 (*Mapk8*). The full names and functions of these genes are listed in Table S4.

### Timing expression of hub genes of injured spinal cord tissue

**Fig. 4:**
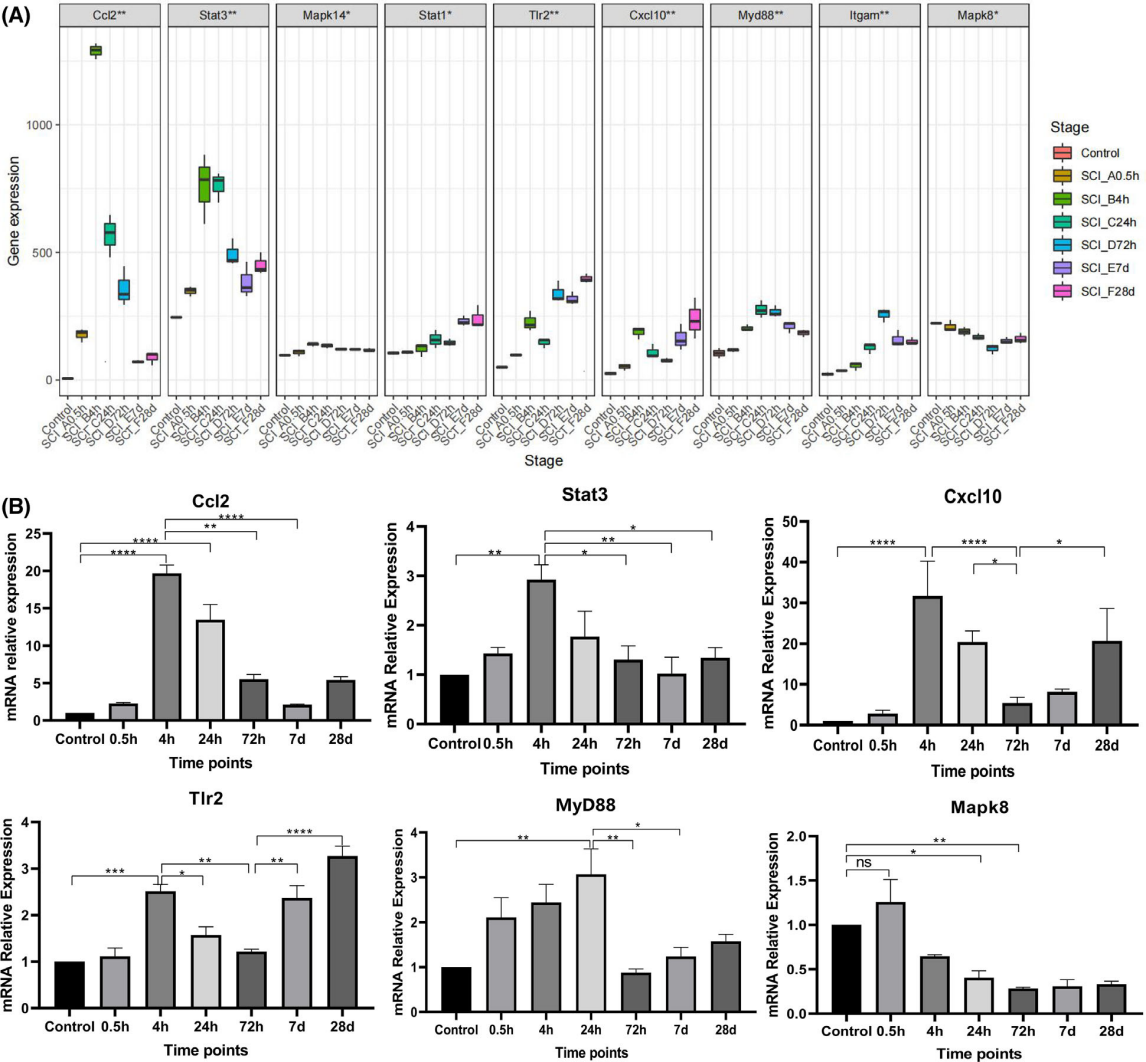
Dynamic expression of hub genes. (A) Trends in the temporal expression of hub genes. The ordinate shows the degree of gene expression, the bottom abscissa shows each timepoint, and the top abscissa shows the names of the genes. (B) The trends in the qPCR time‐series expression levels of various significant hub genes. Each timepoint is represented by the abscissa, and the ordinate shows the relative level of expression of genes relevant to the innate immune system. d, day; h, hour; Values are expressed as the means ± SEM. *n* = 3 for each group. Student's *t*‐test was used. ****P < 0.0001; ***P < 0.005; **P < 0.01; *P < 0.05. A *P* value < 0.5 was regarded as statistically significant.

The dataset analysis showed that the dynamic expression characteristics of the hub genes were different (Fig. 4A). Among them, *Ccl2, Stat3, Mapk14, Cxcl10,* and *Tlr2* peaked at the 4th hour, *Stat1* and *Myd88* peaked at the 24th hour, *Tlr2* and *Itgam* peaked at the 72nd hour, and *Mapk8* was downregulated after SCI. However, *Stat1*, *Tlr2*, and *Cxcl10* still had relatively high expression levels at 7 h and 28 days after SCI.

The verification results of qPCR showed that the changes in the expression levels of *Ccl2*, *Stat3*, *Cxcl10*, and *Tlr2* at each timepoint were consistent with the data analysis. The *Myd88* expression levels were consistent with the change before the 72nd hour in the data analysis. The *Mapk8* expression level was consistent with the data analysis from 4th hour to 28th day after SCI. However, the expression levels of *Itgam*, *Stat1*, and *Mapk14* were not consistent with the data analysis (Fig. 4B).

### Dataset analysis captures characteristics of innate immune cell infiltration in SCI tissues

**Fig. 5:**
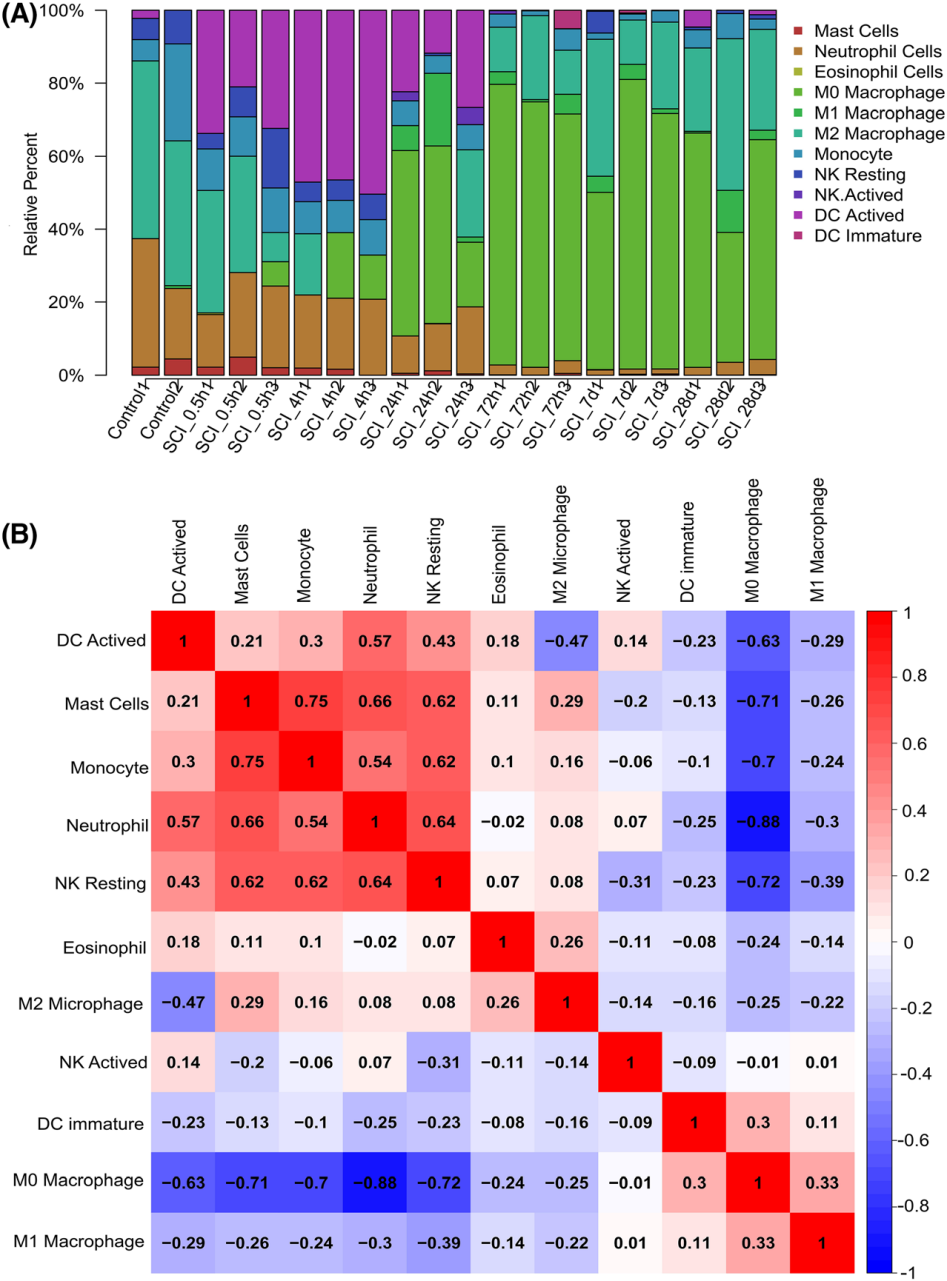
Infiltration of innate immune cells in spinal cord tissue after SCI. (A) The proportion of the 11 innate immune cell subgroups in SCI. Each GEO sample is represented on the *X*‐axis, and the percentage of each type of immune cell is represented on the *Y*‐axis. (B) Heatmap of correlation displaying 11 different types of innate immune cells. The square's color intensity conveys the correlation's strength. A positive correlation is denoted by red, whereas a negative correlation is denoted by blue. con, control; d, days; h, hours.

The infiltration levels of 11 types of innate immune cells in SCI were evaluated. The infiltration level of activated dendritic cells (DCs) increased and peaked at the 4th hour. The infiltration level of neutrophils maintained relatively high within 24 h of SCI, then declined and remained low. The infiltration level of M2‐type macrophages decreased from 0.5 to 24 h after SCI but increased after 72 h and remained at a high state. M1 macrophages began to appear 24 h after SCI and remained until the 28th day. The infiltration of innate immune cells in spinal cord tissues at each timepoint is shown in Fig. 5A.

The relationship among the infiltration of 11 types of innate immune cells was also evaluated. Neutrophils, monocytes, and resting natural killer (NK) cells all were significantly and positively correlated with mast cells, while activated DCs, mast cells, monocytes, neutrophils, and resting NK cells were all negatively correlated with M0 macrophages. Neutrophils were significantly and positively correlated with resting NK cells and activated DCs. Monocytes were obviously and positively correlated with NK cells (Fig. 5B).

### Dataset analysis captures correlation between hub innate immunity‐related genes and innate immune cells

**Fig. 6:**
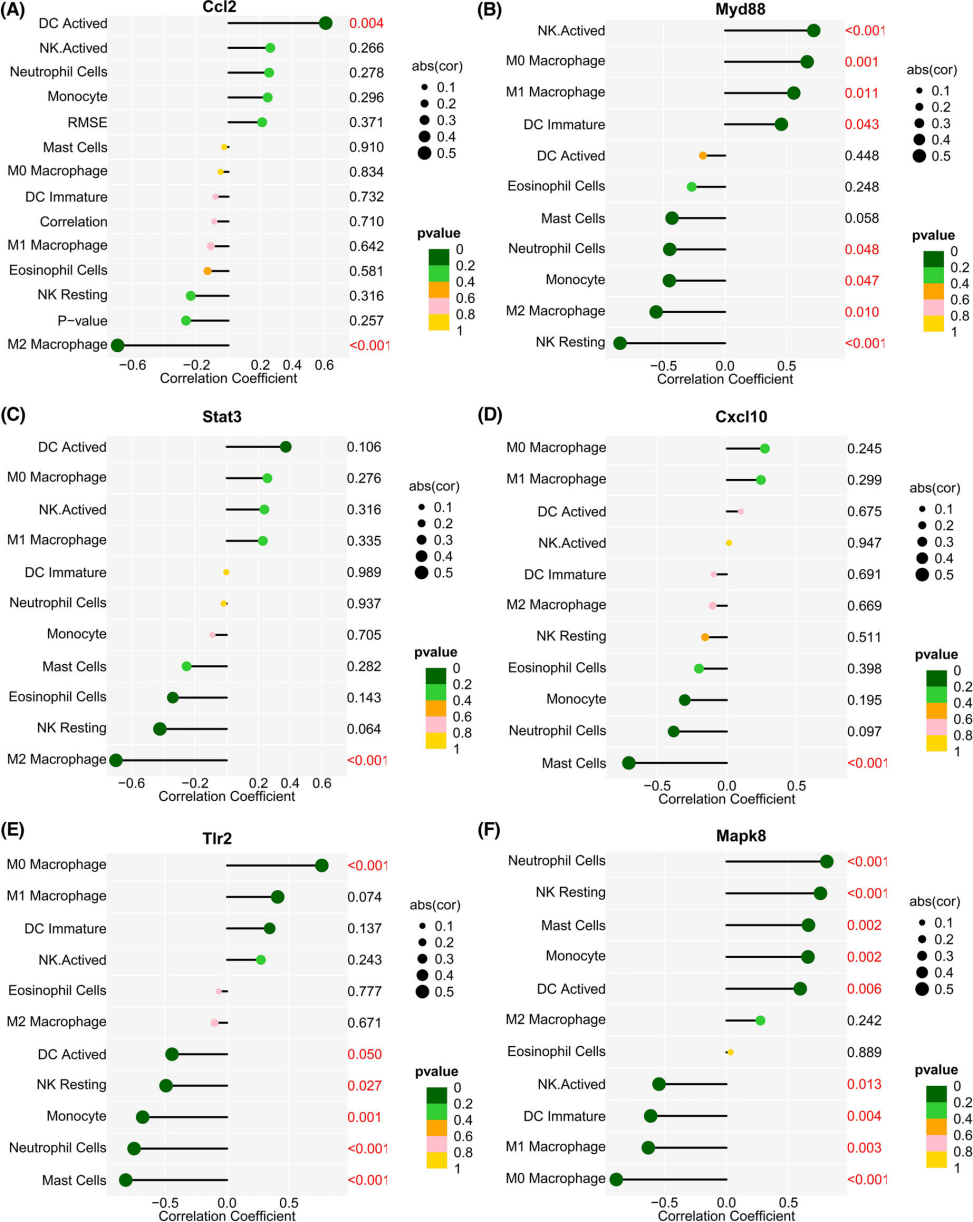
Correlation between innate immune‐related hub genes and innate immune cells. (A) Correlation between *Ccl2* and innate immune cells. (B) Correlation between *Myd88* and innate immune cells. (C) Correlation between *Stat3* and innate immune cells. (D) Correlation between *Cxcl10* and innate immune cells. (E) Correlation between *Tlr2* and innate immune cells. (F) Correlation between *Mapk8* and innate immune cells. The fraction of innate immune cells is represented by the ordinate on the left, the correlation test's *P* value is shown by the ordinate on the right, and the correlation coefficient is shown by the abscissa. The correlation coefficient is shown by the size of the circle, while the correlation test's *P* value is shown by the color of the circle. A *P* value < 0.5 was regarded as statistically significant.

The correlations between the hub innate immune‐related genes and the infiltration level of innate immune cells were analyzed (Fig. 6). *Ccl2* was positively correlated with activated DCs. *Myd88* was positively correlated with M0 macrophages, M1 macrophages, immature DCs, and activated NK cells. *Tlr2* was correlated positively with M0 macrophages, but negatively with activated DCs, NK resting cells, mast cells, monocytes, and neutrophils. *Cxcl10* was negatively correlated with mast cells. *Itgam* was positively correlated with immature DCs, M0 macrophages, M1 macrophages, and activated NK cells. *Mapk8* was positively correlated with neutrophils, NK resting cells, mast cells, monocytes, and activated DCs.

### Single‐cell sequencing dataset analysis

**Fig. 7:**
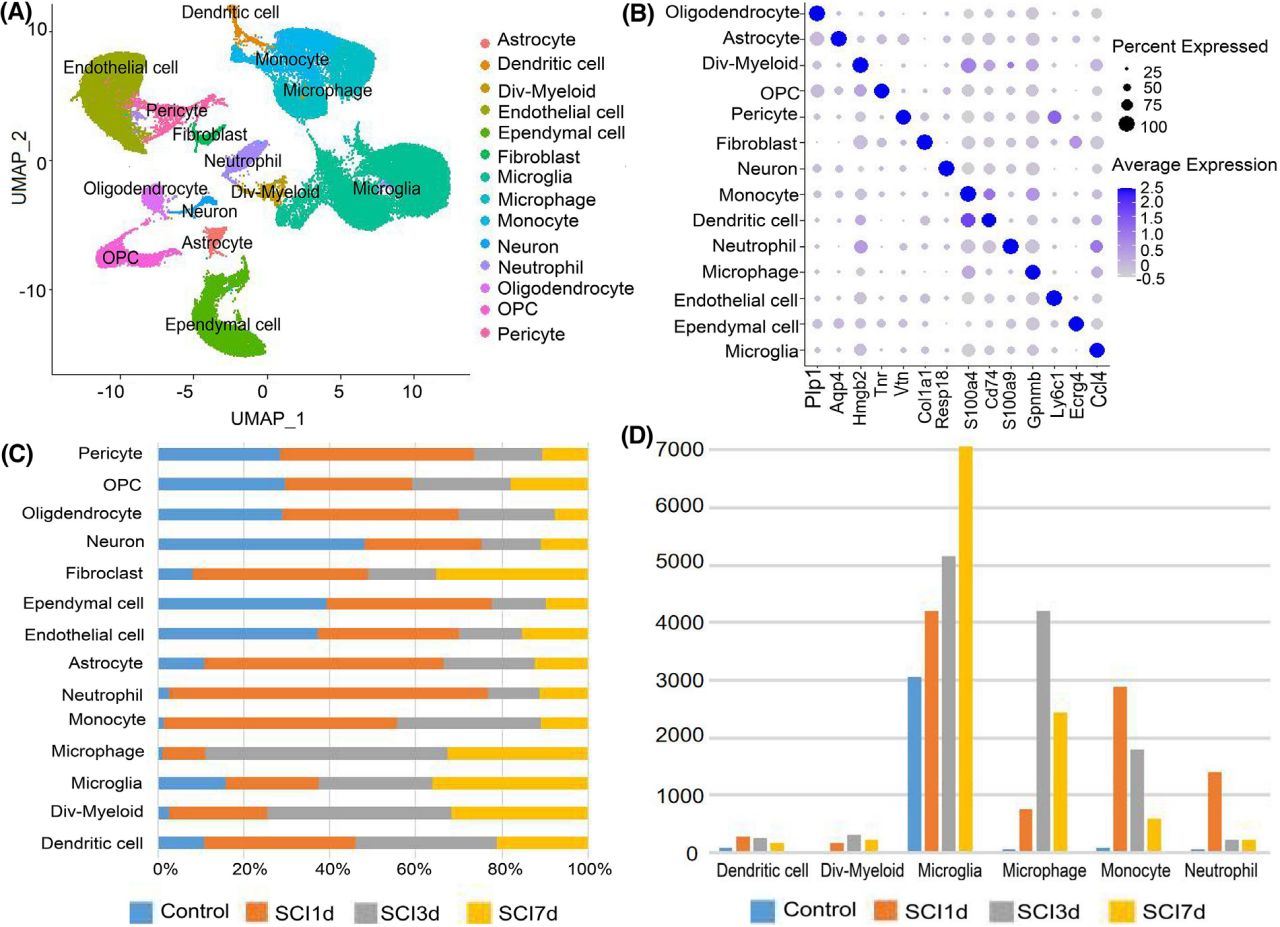
Cell clusters in SCI samples. (A) Cell clustering diagram with annotations. The UMAP graphic shows various cell types in different colors. (B) Cell type‐specific marker genes. The cell type is represented by the ordinate, the gene name by the abscissa, the circle's size by the percentage of that cell type's cells that express the gene, and the level of gene expression is indicated by the depth of the circle's color. (C) The proportion of cells in various samples. The abscissa denotes the proportion, and the ordinate the type of cell. (D) The number of various cell kinds present in the samples at various timepoints. The ordinate denotes the quantity of cells, and the abscissa, the type of cells. d, day; Div‐Myeloid, dividing‐myeloid; OPC, oligodendrocyte progenitor cell.

After batch correction of the data, the cell population was clustered and labeled (Fig. 7A,B). The percentage of homogeneous cells in different samples and the time‐series changes in the number of some innate immune cells were analyzed (Fig. 7C,D). The numbers of DCs, monocytes, and neutrophils all increased significantly on the first day after SCI. The number of neutrophils decreased significantly on the third day, the numbers of macrophages and mitotic myeloid cells peaked on the third day, and the number of microglia peaked on the seventh day.

**Fig. 8:**
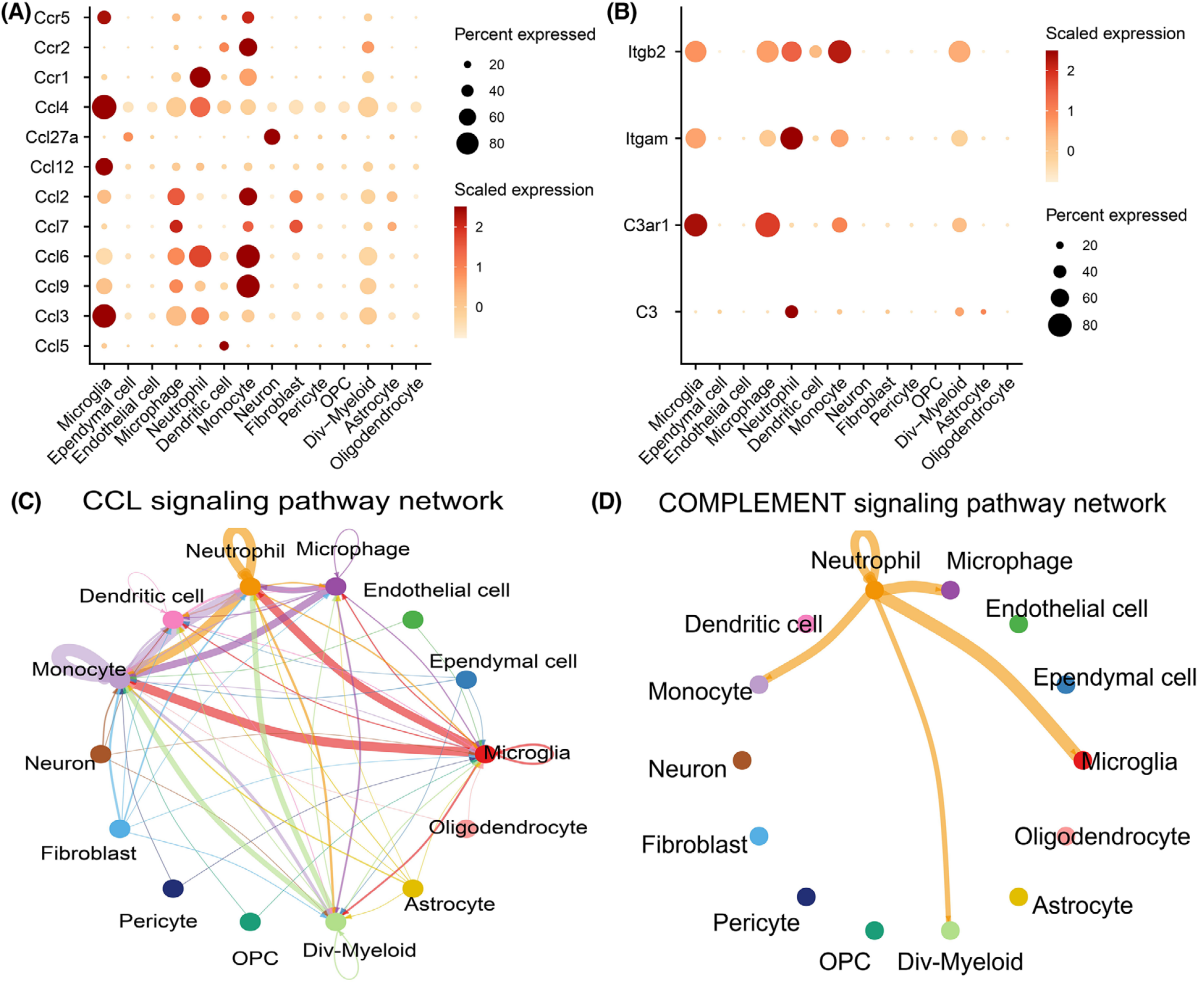
Cell communication in spinal cord after SCI. Expression of receptors and ligands in the CCL and COMPLEMENT signaling pathways (A) and (B). The diameter of the circle denotes the percentage of a cell type's cells that express a certain gene, and the intensity of the color denotes the degree of that expression. Between cells in the SCI, the CCL signaling pathway (C) and the COMPLEMENT signaling pathway (D). The strength of the signal is determined by the intercellular lines' thickness; the thicker the line, the stronger the signal intensity.

The communication relationships among different cells after SCI were analyzed. In the CCL signaling pathway network, the ligand *Ccl2* was mainly expressed in microglia, macrophages, monocytes, fibroblasts, and Dividing‐Myeloid cells. Receptor Ccr2 was mainly expressed in DCs, monocytes, and Dividing‐Myeloid cells (Fig. 8A,C). In the COMPLEMENT signaling pathway network, ligand complement 3 (C3) was mainly expressed in neutrophils and Dividing‐Myeloid cells, while its receptor Itgam was mainly expressed in microglia, macrophages, neutrophils, monocytes, and Dividing‐Myeloid cells (Fig. 8B,D).

**Fig. 9:**
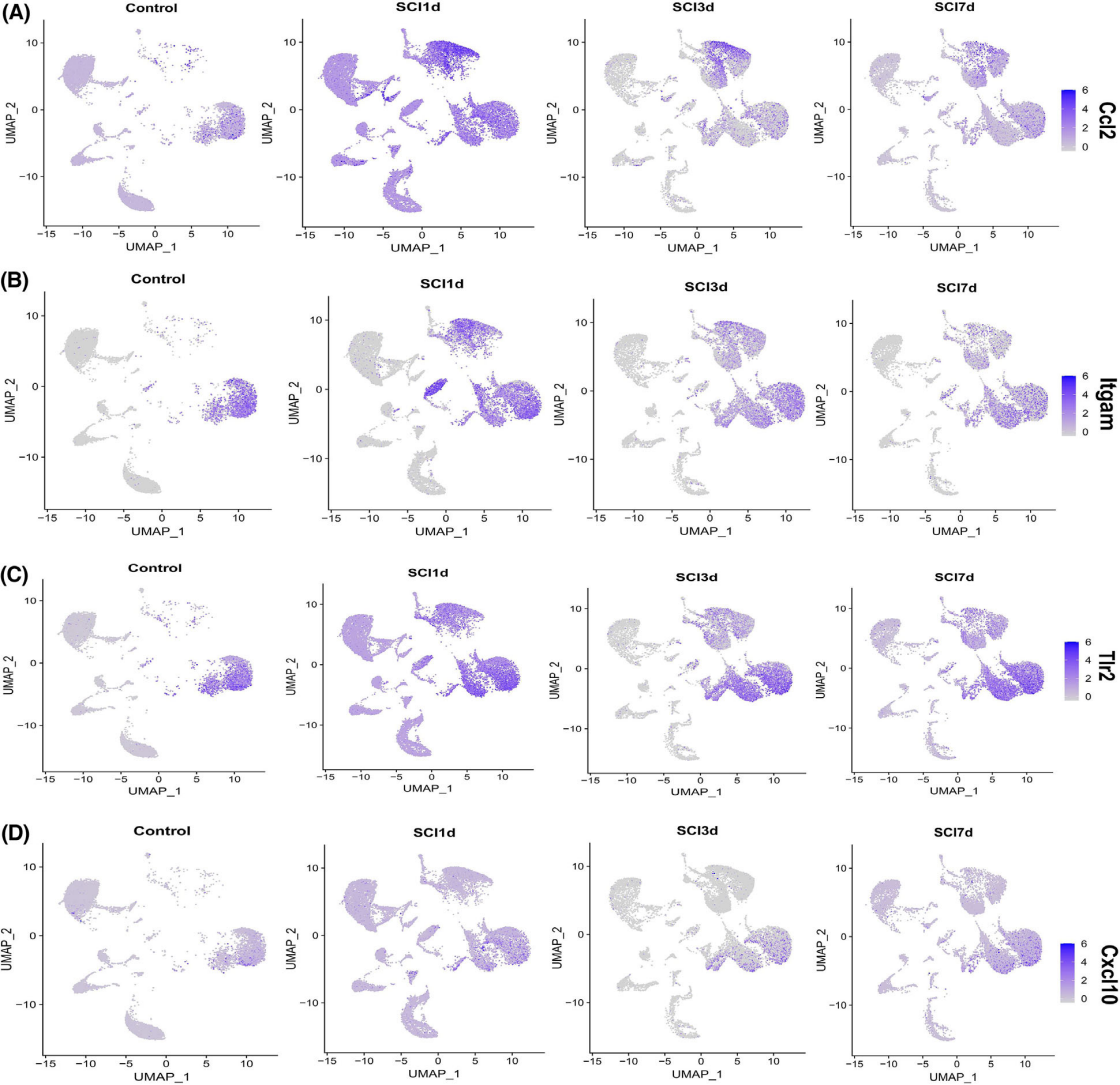
The expression of hub genes in the cells of spinal cord after SCI at different timepoints. (A) Expression of *Ccl2* in spinal cord tissue cells of the control group and each timepoint of SCI group. (B) Expression of *Itgam* in spinal cord tissue cells of control group and each timepoint of the SCI group. (C) Expression of *Tlr2* in spinal cord tissue cells of the control group and each timepoint of the SCI group. (D) Expression of *Cxcl10* in spinal cord tissue cells of the control group and each timepoint of the SCI group. The degree of gene expression is represented by the color intensity in the UMAP graph. d, days.

According to the annotated map of cell clusters (Fig. 7A), the *Ccl2* expression increased significantly at 1 day after SCI, and mainly occurred in macrophages, monocytes, and microglia. After that, the expression decreased and mainly occurred in monocytes and macrophages (Fig. 9A). *Itgam* was also significantly expressed in microglia, macrophages, monocytes, and neutrophils at 1 day after SCI, and the expression decreased significantly thereafter (Fig. 9B). *Tlr2* was mainly expressed in microglia, monocytes, macrophages, and neutrophils at 1 day after SCI, but on the 3rd and 7th day, it was mainly expressed in microglia (Fig. 9C). *Cxcl10* was mainly expressed in microglia after SCI (Fig. 9D).

### Expressions of hub genes in the blood of human SCI

**Fig. 10:**
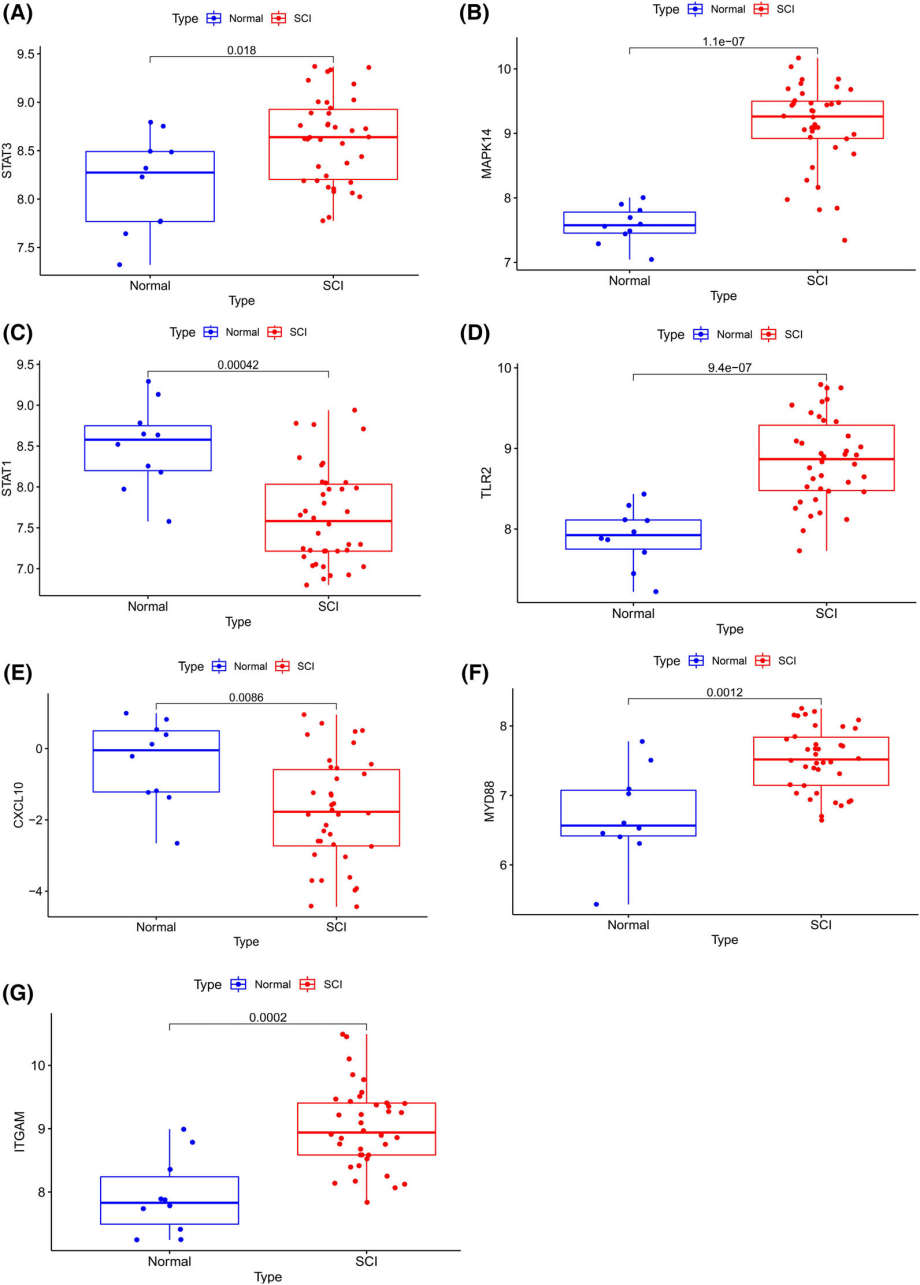
Expression of innate immune‐related genes in the blood cells of human SCI. (A) Differential expression of *STAT3* in blood cells of normal people and patients with SCI. (B) Differential expression of *MAPK14* in blood cells of normal people and patients with SCI. (C) Differential expression of *STAT1* in blood cells of normal people and patients with SCI. (D) Differential expression of *TLR2* in blood cells of normal people and patients with SCI. (E) Differential expression of *CXCL10* in blood cells of normal people and patients with SCI. (F) Differential expression of *MYD88* in blood cells of normal people and patients with SCI. (G) Differential expression of *ITGAM* in blood cells of normal people and patients with SCI. The abscissa denotes the type of sample, while the ordinate denotes the degree of gene expression. The gene name appears as the title of the vertical axis, and the title of the horizontal axis denotes the type of sample. *n* (normal) = 10; *n* (SCI) = 38; Student's *t*‐test was used. Statistical significance was defined as *P* < 0.5.

Analysis of blood immune changes of human SCI showed that the expressions of integrin subunit alpha M (*ITGAM*), mitogen‐activated protein kinase 14 (*MAPK14*), myeloid differentiation primary response 88 (*MYD88*), signal transducer and activator of transcription 3 (*STAT3*), Toll‐like receptor 2 (*TLR2*) in human blood were higher, and the expressions of signal transducer and activator of transcription 1 (*STAT1*) and C‐X‐C motif chemokine ligand 10 (*CXCL10*) were lower compared with the control group. These results suggest that these hub genes may also play an important role in human SCI (Fig. 10).

## Discussion

The blood–brain barrier is destroyed after SCI. The innate immune cells derived from peripheral blood can quickly participate in the regulation of the local microenvironment after SCI and play important roles in secondary SCI and repair [23, 24]. In our study, the differentially‐expressed innate immune‐related genes and hub genes were identified. Then the dynamic changes in the expression levels of these hub genes were analyzed and verified to identify the optimal time to target these genes. Furthermore, the infiltration of innate immune cells and their relationship with hub genes were characterized. Two hub genes related signaling pathways and receptor–ligand pairs were identified in the cells of SCI. These hub genes were also highly expressed in human blood after SCI. This result also reveals that finding characteristic blood immune‐related genes related to the severity of SCI may be of great significance for research, auxiliary diagnosis, and treatment of human SCI.

Dataset analysis showed the number of upregulated innate immune‐related genes was much larger than that of downregulated genes, which indicates a clear innate immune response after SCI. The infiltration levels of DCs and neutrophils increased rapidly within 24 h of SCI, and significantly reduced after 24 h. In addition, the *Ccl2* expression remained at a relatively high level within 24 h of SCI and was positively correlated with the infiltration level of activated DCs, indicating that *Ccl2* may play a relatively important role in the infiltration of activated DCs. Single‐cell sequencing data analysis demonstrated that hub genes were mainly expressed in innate immune cells, such as microglia, macrophages, monocytes, DCs, and neutrophils, but less expressed in glial cells, neurons, endothelial cells, and ependymal cells from spinal cord tissues. This result clarifies the relationship between cells in SCI tissues and the expression of hub genes, and indicates that the innate immune cells derived from peripheral blood and microglia constitute the main innate immune components in SCI.

Due to the breakdown of the blood–brain barrier after human SCI, the peripheral circulation communicates with the SCI area. When immune cells in the blood infiltrate the SCI area, the immune status in the circulating blood also changes [25, 26]. The blood–brain barrier is also destroyed after SCI in mice [27, 28]. Our dataset analysis shows that genes such as *Ccl2*, *Tlr2*, *Itgam*, and *Cxcl10* are highly expressed not only in microglia after SCI in mice, but also in peripheral blood‐derived immune cells such as macrophages, monocytes, and neutrophils. Due to the absence of sequencing data for mouse SCI blood in the database, there is also no sequencing data for human SCI tissues. Therefore, we analyzed the blood sequencing dataset of human SCI to explore the expression of hub genes in peripheral circulating blood after SCI. The blood of these patients was collected at 30.3 ± 18.9 h after SCI to detect the expression of hub genes. Most of the hub genes were also highly expressed in the blood of SCI patients, but only *CXCL10* was downregulated. These results suggest the expression of these genes may also play an important role in the tissues of human SCI, but the current research on the sequential expression of blood genes in human SCI is not comprehensive. Exploring the temporal expression characteristics of hub innate immune‐related genes in the blood of human SCI may have important significance for the diagnosis and treatment of SCI.

From the characteristics of the temporal expressions of hub genes verified above and the infiltration of innate immune cells, we can find a potential suitable time for immunotherapy of SCI in mice, which may experimentally underlie the immunotherapy of SCI. *Ccl2* acts through expressing the C‐C motif chemokine receptor 2 (*Ccr2*), thereby regulating the migration and infiltration of monocytes, T‐lymphocytes, and NK cells into inflammation areas [29, 30]. Our dataset analysis showed that the *Ccl2*‐*Ccr2* receptor ligand pair in the CCL signaling pathway network plays a regulatory role between microglia, macrophages, monocytes, fibroblasts, Dividing‐Myeloid cells and DCs, monocytes, and Dividing‐Myeloid cells. In addition, *Ccl2* can promote the polarization of M2‐type macrophages and enhance the growth of axons in peripheral nerve injury [9]. Inhibiting *Ccr2* after SCI can alleviate SCI in mice [31]. Both our dataset analysis and qPCR validation showed the *Ccl2* expression level quickly peaked at 4 h after SCI, indicating regulating *Ccl2* or its receptor *Ccr2* within 4 h of SCI may promptly promote the recovery of SCI. In addition, *Stat3* controls different pathways of emergency granulocyte production and mature neutrophils [32, 33], the migration of neutrophils [34], and the differentiation of DCs in the innate immune response [35]. Both our dataset analysis and qPCR validation showed that the *Stat3* expression level peaked at the 4th hour after SCI and remained at a high level before the 24th hour. In addition, the dataset analysis showed that the infiltration level of neutrophils and activated DCs also increased rapidly and remained at a higher level within 24 h after SCI, indicating that *Stat3* may play a role in urgently regulating the migration of neutrophils and the activation of DCs. Therefore, regulating *Stat3* within 4 h may better improve SCI recovery.

After peripheral nerve injury, *TLR2* induces the expressions of proinflammatory cytokines in the nerve injury area, then induce the activation and infiltration of macrophages into the nerve injury area, and plays a certain neuroprotective effect [36, 37]. Application of *Tlr2* agonists in the acute phase of SCI can promote spinal cord repair [11]. However, our dataset analysis and qPCR validation both showed that the *Tlr2* expression level not only increased within 4 h after SCI, but also reached higher levels on the 7th and 28th day after SCI. Therefore, regulating *Tlr2* expression in the acute, subacute, and intermediate phases of SCI may help with spinal cord repair. *Cxcl10* mainly mediates Thl‐type inflammatory responses as well as the chemotaxis of monocytes and T cells, prompting these immune cells to migrate to the injury site, strengthen Thl response, and destroy Th2 response [38]. Anti‐*Cxcl10* treatment of SCI in mice can increase neuronal survival and axonal sprouts, reduce cell apoptosis, and promote revascularization of the damaged spinal cords [39, 40, 41]. However, our dataset analysis and qPCR validation both showed that the *Cxcl10* expression level reached a small peak at the 24th hour after SCI but reached a higher level on the 28th day. Therefore, modulating *Cxcl10* in acute, subacute, and intermediate phases may promote the repair of SCI.

*Myd88* is an anchored adaptor protein primarily responsible for directing intracellular signal transduction, integrating, and transducing intracellular signals generated by the Toll‐like receptor (*TLR*) and interleukin 1 receptor type 1 (*IL‐1R*) superfamily. *MyD88* can promote the migration of white blood cells to the damaged area, which is essential for innate immune regulation [42, 43, 44]. Inhibition of the Toll‐like receptor 4 (*TLR4*)/*Myd88*/nuclear factor of kappa light polypeptide gene enhancer in B cells (*NF‐κB*) signaling pathway can reduce apoptosis and inflammation during SCI [45, 46]. In our study, both dataset analysis and qPCR validation showed that the *Myd88* expression level also reached a peak at the 24th hour and remained high at the 72nd hour. The dataset analysis also showed that *Myd88* was also positively correlated with the infiltration level of M1 macrophages and NK activation, suggesting that *Myd88* plays a relatively important role in the proinflammatory response during the acute and subacute phases of SCI. Therefore, inhibiting *Myd88* expression in these two phases of SCI may suppress inflammation and have a better treatment effect on reducing secondary SCI. Interestingly, both dataset analysis and qPCR validation showed that the *Mapk8* expression was downregulated after SCI, and minimized at 72 h after SCI. Only a few articles have studied the *Mapk8* gene in SCI. Therefore, the important role of *Mapk8* in SCI is worthy of further study. Regulating the *Mapk8* expression at about 72 h in the subacute phase may be a focus of research.

*Itgam* , an integrin α‐M, can form a dimer with integrin subunit beta 2 (*Itgb2*) to participate in various adhesion interactions of monocytes, macrophages, and granulocytes, and to mediate the uptake of complement‐coated particles as well as the migration of immune cells to infected or injured areas [47, 48, 49]. *Itgam* can also promote the polarization of microglia and macrophages to the M2 type. Our dataset analysis showed that the *C3*‐(*Itgam*+*Itgb2*) receptor ligand pair in the COMPLEMENT signaling pathway network played a regulatory role between neutrophils, Dividing‐Myeloid cells and microglia, macrophages, neutrophils, monocytes, and Dividing‐Myeloid cells. The dataset analysis also showed that the *Itgam* expression level peaked at 72 h, and then gradually decreased. This result also indicates that regulating *Itgam* before 72 h after SCI may improve spinal cord repair.

However, our data were mainly obtained from analysis of sequencing data, with a brief experimental validation of gene expression using qPCR. In addition, the sample size is relatively small. Although we identified hub genes and found that some hub genes played a certain role in spinal cord repair, intervention only on one gene may not lead to more ideal spinal cord repair results. In the future, *in vivo* and *in vitro* experiments with larger sample sizes are required to analyze and verify the roles of these hub genes in SCI repair.

## Conclusion

This study revealed the characteristics of innate immune cell infiltration and identified hub innate immune‐related genes. The characteristics of temporal expression of these genes after SCI may have certain significance in allowing us to intervene in these hub genes and innate immune cells at the right time to reduce secondary SCI and promote spinal cord repair. These hub innate immune‐related genes are also highly expressed in human blood, and the temporal changes of the innate immunity of human SCI blood are worthy of further study. Therefore, this study provides certain ideas and the basis for immunotherapy of SCI. We anticipate that our research can provide certain experimental basis and ideas for the research and treatment of SCI.

## Conflict of interest

The authors declare no conflicts of interest.

## Author contributions

JL involved in methodology, formal analysis, investigation, and writing—original draft. XL involved in methodology, investigation, and writing—original draft. HW involved in formal analysis, investigation, and writing—review and editing. PG involved in methodology and writing—review and editing. BL involved in methodology and investigation. JW involved in conceptualization and writing—review and editing. WT involved in visualization and funding acquisition. DC involved in conceptualization, project administration and funding acquisition. MG involved in conceptualization, data curation, writing—review and editing, revision, and project administration. ZZ involved in conceptualization, data curation, writing—review and editing, supervision, project administration, and funding acquisition. SL involved in conceptualization, resources, supervision, project administration, and funding acquisition.

## Supporting information


**Table S1.** Differentially expressed innate immune‐related genes between control group and SCI group at each timepoint.
**Table S1A.** Differentially expressed innate immune‐related genes at 0.5 hours after SCI.
**Table S1B.** Differentially expressed innate immune‐related genes at 4 hours after SCI.
**Table S1C.** Differentially expressed innate immune‐related genes at 24 hours after SCI.
**Table S1D.** Differentially expressed innate immune‐related genes at 72 hours after SCI.
**Table S1E.** Differentially expressed innate immune‐related genes at 7 days after SCI.
**Table S1F.** Differentially expressed innate immune‐related genes at 28 days after SCI.
**Table S2.** Thirty GO terms with the lowest P value at each timepoint.
**Table S3.** Ten KEGG terms of upregulated immune‐related genes and downregulated immune‐related genes with the lowest P values at each timepoint.
**Table S4.** Annotation of hub innate immune‐related genes.

## Data Availability

Publicly available datasets were analyzed in this study. All of the raw data used in this study are derived from the public GEO data portal (https://www.ncbi.nlm.nih.gov/geo/; Accession numbers: GSE5296, GSE162610, GSE151371).
