# Supplementary material for: Identification of hub genes related to the innate immune response activated during spinal cord injury
Source: FEBS Open Bio. 2022 Sep 1;12(10):1839–56. doi: 10.1002/2211-5463.13472 (PMC9527585; doi:10.1002/2211-5463.13472)
Supplement: Supplementary file 1 — Table S1. Differentially expressed innate immune‐related genes between control group and SCI group at each timepoint. Table S1A. Differentially expressed innate immune‐related genes at 0.5 hours after SCI. Table S1B. Differentially expressed innate immune‐related genes at 4 hours after SCI. Table S1C. Differentially expressed innate immune‐related genes at 24 hours after SCI. Table S1D. Differentially expressed innate immune‐related genes at 72 hours after SCI. Table S1E. Differentially expressed innate immune‐related genes at 7 days after SCI. Table S1F. Differentially expressed innate immune‐related genes at 28 days after SCI. Table S2. Thirty GO terms with the lowest P value at each timepoint. Table S3. Ten KEGG terms of upregulated immune‐related genes and downregulated immune‐related genes with the lowest P values at each timepoint. Table S4. Annotation of hub innate immune‐related genes. [file FEB4-12-1839-s001.doc]

**Supplementary Tables**

**Identification of hub genes related to the innate immune response activated during spinal cord injury**

Jianfeng Li1,2, #, Xizhe Liu2, #, Huachuan Wu1,2, #, Peng Guo1, Baoliang Li1, Jianmin Wang1, Wei Tian5, Dafu Chen5, Manman Gao3,4, *, Zhiyu Zhou1,2, *, Shaoyu Liu1,2

1Innovation Platform of Regeneration and Repair of Spinal Cord and Nerve Injury, Department of Orthopedic Surgery, The Seventh Affiliated Hospital, Sun Yat-sen University, Shenzhen, Guangdong, China, 518107

2Guangdong Provincial Key Laboratory of Orthopedics and Traumatology, Orthopedic Research Institute/Department of Spinal Surgery, The First Affiliated Hospital of Sun Yat-sen University, Guangzhou, Guangdong, China, 510080

3Department of Sport Medicine, Inst Translat Med, The First Affiliated Hospital of Shenzhen University, Shenzhen Second People, s Hospital, Shenzhen, Guangdong, China, 518000

4Guangdong Key Laboratory for Biomedical Measurements and Ultrasound Imaging, School of Biomedical Engineering, Shenzhen University Health Science Center, Shenzhen, Guangdong, China, 518000

5Laboratory of Bone Tissue Engineering, Beijing Laboratory of Biomedical Materials, Beijing Research Institute of Orthopedics and Traumatology, Beijing Jishuitan Hospital, Beijing, China, 100035

***Correspondence:**

Zhiyu Zhou, Ph.D., M.D., E-mail: zhouzhy23@mail.sysu.edu.cn.

Manman Gao, Ph.D., M.D., E-mail: gaomanm@mail2.sysu.edu.cn.

Correspondence to: Zhiyu Zhou, Department of Orthopedic Surgery, The Seventh Affiliated

Hospital, Sun Yat-sen University, 518107, Shenzhen, China

Telephone number: +8613316942792

Email: [zhouzhy23@mail.sysu.edu.cn](mailto:zhouzhy23@mail.sysu.edu.cn)

Correspondence to: Manman Gao, Department of Sport Medicine, The First Affiliated Hospital of Shenzhen University, 518000, Shenzhen, China

Telephone number: +8613570217044

Email: gaomanm@mail2.sysu.edu.cn.

**Supplemental Table 1 Differentially expressed innate immune-related genes between control group**

**and SCI group at each time point.**

Supplemental Table 1A Differentially expressed innate immune-related genes at 0.5 hours after SCI

| Gene Type | Gene | adj.P.Val |
| --- | --- | --- |
| Upregulated gene | Dusp1 | 0.00723312 |
| Upregulated gene | Zfp36 | 0.010066382 |
| Upregulated gene | Klf4 | 0.013631969 |
| Upregulated gene | C1qa | 0.019285455 |
| Upregulated gene | Ccl2 | 0.048860872 |
| Upregulated gene | Serpine1 | 0.048860872 |
| Downregulated gene | Crcp | 0.034621375 |

Supplemental Table 1B Differentially expressed innate immune-related genes at 4 hours after SCI

| Gene type | Gene | adj.P.Val |
| --- | --- | --- |
| Upregulated gene | Lcn2 | 0.000521671 |
| Upregulated gene | Cd14 | 0.014418447 |
| Upregulated gene | Dusp1 | 0.002751695 |
| Upregulated gene | Il33 | 0.030504631 |
| Upregulated gene | Nfkbia | 0.014418447 |
| Upregulated gene | Il6 | 0.023282877 |
| Upregulated gene | Ccl2 | 0.000157578 |
| Upregulated gene | Cxcl1 | 0.015378998 |
| Upregulated gene | Cebpd | 0.000157578 |
| Upregulated gene | Cryab | 0.030504631 |
| Upregulated gene | Zfp36 | 0.009384891 |
| Upregulated gene | Srxn1 | 0.017925654 |
| Upregulated gene | Atf3 | 0.000820291 |
| Upregulated gene | Ptx3 | 0.017744092 |
| Upregulated gene | Cebpb | 0.003357778 |
| Upregulated gene | Stat3 | 0.041182879 |
| Upregulated gene | Myc | 0.01010446 |
| Upregulated gene | Rela | 0.030504631 |
| Upregulated gene | Id2 | 0.028795578 |
| Upregulated gene | S100a10 | 0.009384891 |
| Upregulated gene | Anxa2 | 0.015829139 |
| Upregulated gene | Lgals3 | 0.03715372 |
| Upregulated gene | Atf4 | 0.014065706 |
| Upregulated gene | Hmox1 | 0.000940035 |
| Upregulated gene | Ctsb | 0.030504631 |
| Upregulated gene | Tnfrsf1a | 0.004604474 |
| Upregulated gene | Tyrobp | 0.020501768 |
| Upregulated gene | Sqstm1 | 0.030504631 |
| Upregulated gene | Icam1 | 0.015378998 |
| Upregulated gene | Plaur | 0.030504631 |
| Upregulated gene | Gbp2 | 0.026543044 |
| Upregulated gene | Nfil3 | 0.034513726 |
| Upregulated gene | Nfkb1 | 0.015829139 |
| Upregulated gene | Tollip | 0.046827386 |
| Upregulated gene | Tlr2 | 0.030504631 |
| Upregulated gene | Bcl3 | 0.014418447 |
| Upregulated gene | Irgm1 | 0.033076607 |
| Upregulated gene | Ipo7 | 0.026543044 |
| Upregulated gene | Cxcl10 | 0.015829139 |
| Upregulated gene | Igtp | 0.033076607 |
| Upregulated gene | Birc3 | 0.015378998 |
| Upregulated gene | Mapkapk2 | 0.030504631 |
| Upregulated gene | Pros1 | 0.005790006 |
| Upregulated gene | Csf1 | 0.020488589 |
| Upregulated gene | Hs2st1 | 0.015540601 |
| Upregulated gene | Tnfaip3 | 0.006350345 |
| Upregulated gene | Bcl10 | 0.030504631 |
| Upregulated gene | Myd88 | 0.034513726 |
| Upregulated gene | Il1r1 | 0.006853928 |
| Upregulated gene | Jak2 | 0.034513726 |
| Upregulated gene | C3ar1 | 0.029219217 |
| Upregulated gene | Ptges | 0.037650623 |
| Upregulated gene | Nr4a3 | 0.009384891 |
| Upregulated gene | Ifit1 | 0.034513726 |
| Upregulated gene | Cxcl16 | 0.014065706 |
| Upregulated gene | Pmaip1 | 0.041182879 |
| Upregulated gene | Il4ra | 0.015378998 |
| Upregulated gene | Cd86 | 0.034513726 |
| Upregulated gene | Il1a | 0.023282877 |
| Upregulated gene | Tnfsf9 | 0.026543044 |
| Upregulated gene | Tlr13 | 0.020488589 |
| Upregulated gene | Nlrc5 | 0.026543044 |
| Upregulated gene | Fgr | 0.030504631 |
| Upregulated gene | Iigp1 | 0.015716851 |
| Upregulated gene | Mapk14 | 0.030504631 |
| Upregulated gene | Eif4ebp1 | 0.03715372 |
| Upregulated gene | Tgfb1 | 0.029219217 |
| Upregulated gene | Cflar | 0.030504631 |
| Upregulated gene | Zc3h12a | 0.034513726 |
| Upregulated gene | Casp8 | 0.045352647 |
| Downregulated gene | Rgmb | 0.04605089 |
| Downregulated gene | Trib2 | 0.044054621 |
| Downregulated gene | Ppargc1a | 0.030504631 |
| Downregulated gene | Vldlr | 0.026543044 |
| Downregulated gene | Trim13 | 0.03715372 |
| Downregulated gene | Ccdc88a | 0.029278015 |
| Downregulated gene | Ptch1 | 0.006853928 |
| Downregulated gene | Trim59 | 0.005790006 |
| Downregulated gene | Csf1r | 0.042210713 |
| Downregulated gene | Pltp | 0.039746 |

Supplemental Table 1C Differentially expressed innate immune-related genes at 24 hours after SCI

| Gene type | Gene | adj.P.Val |
| --- | --- | --- |
| Upregulated gene | Spp1 | 0.00235201 |
| Upregulated gene | Lgals3 | 0.00505362 |
| Upregulated gene | Il33 | 0.022326171 |
| Upregulated gene | S100a10 | 0.00235201 |
| Upregulated gene | Hmox1 | 0.023821238 |
| Upregulated gene | Serpine1 | 0.004563678 |
| Upregulated gene | Anxa2 | 0.013830394 |
| Upregulated gene | Lcn2 | 0.016904279 |
| Upregulated gene | Tyrobp | 0.004037283 |
| Upregulated gene | Ctsb | 0.006511971 |
| Upregulated gene | Ctss | 0.00542556 |
| Upregulated gene | Ppp1ca | 0.022108529 |
| Upregulated gene | Ifitm2 | 0.02199916 |
| Upregulated gene | Hspd1 | 0.008884509 |
| Upregulated gene | Cebpd | 0.005388485 |
| Upregulated gene | Cd14 | 0.006869713 |
| Upregulated gene | Cyba | 0.004037283 |
| Upregulated gene | Grn | 0.004563678 |
| Upregulated gene | Itgb2 | 0.02292605 |
| Upregulated gene | C1qa | 0.02798172 |
| Upregulated gene | Ccl2 | 0.006869713 |
| Upregulated gene | Tnfrsf1a | 0.010758356 |
| Upregulated gene | Stat3 | 0.004872899 |
| Upregulated gene | Ly86 | 0.044670065 |
| Upregulated gene | Gabarap | 0.012127791 |
| Upregulated gene | Srxn1 | 0.004563678 |
| Upregulated gene | Xbp1 | 0.004371643 |
| Upregulated gene | Cdc42 | 0.005928701 |
| Upregulated gene | Clec4d | 0.004563678 |
| Upregulated gene | Thbs1 | 0.019790396 |
| Upregulated gene | C1qb | 0.017449484 |
| Upregulated gene | Pros1 | 0.00235201 |
| Upregulated gene | Gbp2 | 0.017533835 |
| Upregulated gene | C1qc | 0.046479483 |
| Upregulated gene | Atf4 | 0.007234667 |
| Upregulated gene | Dusp1 | 0.032829669 |
| Upregulated gene | Rela | 0.018001428 |
| Upregulated gene | Atf3 | 0.006683952 |
| Upregulated gene | Msrb1 | 0.013040515 |
| Upregulated gene | Cebpb | 0.012569691 |
| Upregulated gene | Ppp1cc | 0.022693954 |
| Upregulated gene | Dhx15 | 0.02199916 |
| Upregulated gene | C3ar1 | 0.004563678 |
| Upregulated gene | Plec | 0.004867614 |
| Upregulated gene | Myc | 0.022901264 |
| Upregulated gene | Ltbr | 0.02044711 |
| Upregulated gene | Cd300lf | 0.004563678 |
| Upregulated gene | Igtp | 0.005315259 |
| Upregulated gene | Gnai3 | 0.005150326 |
| Upregulated gene | Ptx3 | 0.02798172 |
| Upregulated gene | Cops5 | 0.00901293 |
| Upregulated gene | Clec7a | 0.005150326 |
| Upregulated gene | Anxa1 | 0.009739124 |
| Upregulated gene | Eif4ebp1 | 0.004037283 |
| Upregulated gene | Irgm1 | 0.013934028 |
| Upregulated gene | Bcl3 | 0.005388485 |
| Upregulated gene | Plaur | 0.006613141 |
| Upregulated gene | Arf6 | 0.004563678 |
| Upregulated gene | Zfp36 | 0.005928701 |
| Upregulated gene | Eif4e | 0.016405565 |
| Upregulated gene | Clec4n | 0.006765283 |
| Upregulated gene | Msr1 | 0.004037283 |
| Upregulated gene | Hdac1 | 0.019032026 |
| Upregulated gene | Myd88 | 0.017449484 |
| Upregulated gene | Lgals9 | 0.005928701 |
| Upregulated gene | Cxcl2 | 0.03210017 |
| Upregulated gene | Ddx21 | 0.005388485 |
| Upregulated gene | Oasl2 | 0.016311053 |
| Upregulated gene | Nras | 0.005928701 |
| Upregulated gene | Hs2st1 | 0.006749183 |
| Upregulated gene | Pycard | 0.007234667 |
| Upregulated gene | Trim30a | 0.004037283 |
| Upregulated gene | Fcgr1 | 0.004563678 |
| Upregulated gene | Tlr13 | 0.004037283 |
| Upregulated gene | Csf1 | 0.006869713 |
| Upregulated gene | Kitl | 0.004456627 |
| Upregulated gene | Slc11a1 | 0.004867614 |
| Upregulated gene | Axl | 0.022693954 |
| Upregulated gene | Casp8 | 0.006613141 |
| Upregulated gene | Cfp | 0.024722764 |
| Upregulated gene | Abca1 | 0.025578352 |
| Upregulated gene | Eps8 | 0.030895195 |
| Upregulated gene | Ptpn11 | 0.017449484 |
| Upregulated gene | Cd97 | 0.025230929 |
| Upregulated gene | Pla2g4a | 0.045918407 |
| Upregulated gene | Cebpa | 0.013034265 |
| Upregulated gene | Il4ra | 0.016405565 |
| Upregulated gene | Cxcl1 | 0.005388485 |
| Upregulated gene | Itgam | 0.016311053 |
| Upregulated gene | Il1rap | 0.01259182 |
| Upregulated gene | Hck | 0.00911758 |
| Upregulated gene | Ptpn6 | 0.013934028 |
| Upregulated gene | Tlr2 | 0.013934028 |
| Upregulated gene | Icam1 | 0.011456787 |
| Upregulated gene | Tgfb1 | 0.008884509 |
| Upregulated gene | Psmb8 | 0.016311053 |
| Upregulated gene | Plcg2 | 0.00901293 |
| Upregulated gene | Trim25 | 0.005315259 |
| Upregulated gene | Cxcl10 | 0.043701417 |
| Upregulated gene | Lrrfip2 | 0.022036519 |
| Upregulated gene | Edn1 | 0.012569691 |
| Upregulated gene | Iigp1 | 0.033908003 |
| Upregulated gene | Jak2 | 0.00901293 |
| Upregulated gene | Bcl10 | 0.012317596 |
| Upregulated gene | Cd48 | 0.00911758 |
| Upregulated gene | Nfe2l2 | 0.038503759 |
| Upregulated gene | Mertk | 0.02655673 |
| Upregulated gene | Il1r1 | 0.034819952 |
| Upregulated gene | Pyhin1 | 0.00901293 |
| Upregulated gene | Klf4 | 0.022099682 |
| Upregulated gene | Ddx41 | 0.039343768 |
| Upregulated gene | Akt1 | 0.034393538 |
| Upregulated gene | Cd36 | 0.046539181 |
| Upregulated gene | Stat6 | 0.012569691 |
| Upregulated gene | 5-Mar | 0.019220787 |
| Upregulated gene | Inpp5d | 0.010948838 |
| Upregulated gene | Clec4e | 0.004563678 |
| Upregulated gene | Cr1l | 0.007344364 |
| Upregulated gene | Polr3d | 0.048531692 |
| Upregulated gene | Irf8 | 0.03714126 |
| Upregulated gene | Rac2 | 0.03210017 |
| Upregulated gene | Tnfaip8l2 | 0.020962402 |
| Upregulated gene | Gbp6 | 0.033399039 |
| Upregulated gene | H2-Ab1 | 0.021978127 |
| Upregulated gene | Notch1 | 0.040770243 |
| Upregulated gene | Fgr | 0.02380746 |
| Upregulated gene | Ripk3 | 0.012801504 |
| Upregulated gene | Traf2 | 0.017070326 |
| Upregulated gene | Trim15 | 0.016311053 |
| Upregulated gene | Mefv | 0.012569691 |
| Upregulated gene | Adam17 | 0.01970329 |
| Upregulated gene | Casp1 | 0.012569691 |
| Upregulated gene | Zc3hav1 | 0.007234667 |
| Upregulated gene | Btk | 0.027451735 |
| Upregulated gene | Rab8a | 0.037457885 |
| Upregulated gene | Polr3h | 0.03047204 |
| Upregulated gene | Naip2 | 0.028647899 |
| Upregulated gene | Eif2ak2 | 0.038526897 |
| Upregulated gene | Ifnar1 | 0.035724949 |
| Upregulated gene | Mapk14 | 0.02655673 |
| Upregulated gene | Lcp2 | 0.034669827 |
| Upregulated gene | Scaf11 | 0.037579708 |
| Upregulated gene | Trim21 | 0.016904279 |
| Upregulated gene | Herc6 | 0.023821238 |
| Upregulated gene | Ikbke | 0.03047204 |
| Upregulated gene | Unc93b1 | 0.013680855 |
| Upregulated gene | Apobec3 | 0.025578352 |
| Upregulated gene | Lst1 | 0.021978127 |
| Upregulated gene | Plscr1 | 0.049781673 |
| Upregulated gene | Irf5 | 0.025578352 |
| Upregulated gene | Ptges | 0.022693954 |
| Downregulated gene | Tsc1 | 0.039343768 |
| Downregulated gene | Cd55 | 0.036893257 |
| Downregulated gene | Angpt1 | 0.027249958 |
| Downregulated gene | Sema3a | 0.01776662 |
| Downregulated gene | Zfpm2 | 0.017449484 |
| Downregulated gene | Drd2 | 0.016439103 |
| Downregulated gene | Tlr3 | 0.023034704 |
| Downregulated gene | Arntl | 0.039145595 |
| Downregulated gene | Nr3c1 | 0.019032026 |
| Downregulated gene | Trpm2 | 0.01206799 |
| Downregulated gene | Mapk8 | 0.023371243 |
| Downregulated gene | Map4k2 | 0.00901293 |
| Downregulated gene | Cyld | 0.02380746 |
| Downregulated gene | Rgs2 | 0.046891693 |
| Downregulated gene | Trib2 | 0.038560741 |
| Downregulated gene | Traf3 | 0.03714126 |
| Downregulated gene | Vldlr | 0.021978127 |
| Downregulated gene | Tbk1 | 0.013934028 |
| Downregulated gene | Gpsm1 | 0.015668996 |
| Downregulated gene | Pik3c3 | 0.037850379 |
| Downregulated gene | Tyro3 | 0.012569691 |
| Downregulated gene | Trim13 | 0.025578352 |
| Downregulated gene | Trim59 | 0.013879424 |
| Downregulated gene | Edil3 | 0.00911758 |
| Downregulated gene | Ppargc1b | 0.024314914 |
| Downregulated gene | Ppargc1a | 0.019032026 |
| Downregulated gene | C3 | 0.03714126 |
| Downregulated gene | Snca | 0.032249554 |
| Downregulated gene | Fbxo9 | 0.033218505 |
| Downregulated gene | Ptch1 | 0.004037283 |
| Downregulated gene | Tril | 0.012569691 |
| Downregulated gene | Trim32 | 0.011456787 |
| Downregulated gene | Chga | 0.022693954 |
| Downregulated gene | Pacsin1 | 0.027893276 |
| Downregulated gene | Stmn1 | 0.030503398 |
| Downregulated gene | Pltp | 0.005388485 |
| Downregulated gene | Cd81 | 0.044802669 |

Supplemental Table 1D Differentially expressed innate immune-related genes at 72 hours after SCI

| Gene type | Gene | adj.P.Val |
| --- | --- | --- |
| Upregulated gene | Spp1 | 0.00013726 |
| Upregulated gene | Lgals3 | 0.001141035 |
| Upregulated gene | Ctss | 0.001287248 |
| Upregulated gene | C1qa | 0.006462915 |
| Upregulated gene | C1qc | 0.00796831 |
| Upregulated gene | Tyrobp | 0.00689851 |
| Upregulated gene | Grn | 0.000782241 |
| Upregulated gene | Hmox1 | 0.023093017 |
| Upregulated gene | C1qb | 0.001749502 |
| Upregulated gene | Lgmn | 0.000782241 |
| Upregulated gene | Ctsb | 0.001749502 |
| Upregulated gene | Cyba | 0.004542052 |
| Upregulated gene | Ly86 | 0.003506048 |
| Upregulated gene | Rpl19 | 0.025960264 |
| Upregulated gene | Csf1r | 0.003275364 |
| Upregulated gene | Gabarap | 0.001270119 |
| Upregulated gene | Ifitm2 | 0.004395361 |
| Upregulated gene | Itgb2 | 0.004720948 |
| Upregulated gene | S100a10 | 0.006717012 |
| Upregulated gene | Il33 | 0.013235483 |
| Upregulated gene | Dcn | 0.003506048 |
| Upregulated gene | Abca1 | 0.028256161 |
| Upregulated gene | C3ar1 | 0.000904948 |
| Upregulated gene | Cdc42 | 0.004395361 |
| Upregulated gene | Ppp1ca | 0.00789307 |
| Upregulated gene | Anxa2 | 0.003959787 |
| Upregulated gene | Fstl1 | 0.028801918 |
| Upregulated gene | Msrb1 | 0.003925917 |
| Upregulated gene | Thbs1 | 0.002971573 |
| Upregulated gene | Cav1 | 0.008438876 |
| Upregulated gene | Hsp90b1 | 0.039083595 |
| Upregulated gene | Clec4d | 0.006554163 |
| Upregulated gene | Lgals9 | 0.002394587 |
| Upregulated gene | Lum | 0.00796831 |
| Upregulated gene | Tnfrsf1a | 0.003566299 |
| Upregulated gene | Ybx1 | 0.004542052 |
| Upregulated gene | Nfkbia | 0.032415205 |
| Upregulated gene | Anxa1 | 0.006357014 |
| Upregulated gene | Slc11a1 | 0.002326275 |
| Upregulated gene | Serping1 | 0.015820308 |
| Upregulated gene | Pros1 | 0.00789307 |
| Upregulated gene | Cd36 | 0.036233872 |
| Upregulated gene | Cd48 | 0.034011195 |
| Upregulated gene | Xbp1 | 0.003055086 |
| Upregulated gene | Cd14 | 0.001115311 |
| Upregulated gene | Cebpd | 0.009108058 |
| Upregulated gene | Serpine1 | 0.035396121 |
| Upregulated gene | Cebpa | 0.001287248 |
| Upregulated gene | Atf3 | 0.001287248 |
| Upregulated gene | Cfh | 0.007475459 |
| Upregulated gene | Gnai2 | 0.028796986 |
| Upregulated gene | Hdac1 | 0.001287248 |
| Upregulated gene | Unc93b1 | 0.012391093 |
| Upregulated gene | Mapk3 | 0.005220225 |
| Upregulated gene | Ccl2 | 0.010498383 |
| Upregulated gene | Lcn2 | 0.027711612 |
| Upregulated gene | Hif1a | 0.000782241 |
| Upregulated gene | Casp8 | 0.007486273 |
| Upregulated gene | Tlr13 | 0.002326275 |
| Upregulated gene | Sirpa | 0.031967239 |
| Upregulated gene | Arf6 | 0.001158354 |
| Upregulated gene | Cebpb | 0.006303123 |
| Upregulated gene | Txnip | 0.009498282 |
| Upregulated gene | Rac1 | 0.047316466 |
| Upregulated gene | Lbp | 0.010400863 |
| Upregulated gene | Msr1 | 0.00013726 |
| Upregulated gene | Dhx15 | 0.002326275 |
| Upregulated gene | Mfge8 | 0.023093017 |
| Upregulated gene | Tlr2 | 0.004542052 |
| Upregulated gene | C3 | 0.032819403 |
| Upregulated gene | Srxn1 | 0.004542052 |
| Upregulated gene | Stat3 | 0.009950239 |
| Upregulated gene | Clec4n | 0.003055086 |
| Upregulated gene | Gnai3 | 0.001287248 |
| Upregulated gene | Cxcr4 | 0.006380454 |
| Upregulated gene | Itgam | 0.003275364 |
| Upregulated gene | Irf8 | 0.007105915 |
| Upregulated gene | Pycard | 0.001141035 |
| Upregulated gene | C5ar1 | 0.003309536 |
| Upregulated gene | Cd300lf | 0.003403963 |
| Upregulated gene | Ppp1cc | 0.036188648 |
| Upregulated gene | Mertk | 0.003570419 |
| Upregulated gene | Eif4ebp1 | 0.004970581 |
| Upregulated gene | Cfp | 0.003925917 |
| Upregulated gene | Fcgr1 | 0.000917668 |
| Upregulated gene | Ptpn6 | 0.001749502 |
| Upregulated gene | Plcg2 | 0.008408461 |
| Upregulated gene | Axl | 0.001749502 |
| Upregulated gene | H2-Aa | 0.002326275 |
| Upregulated gene | Ezh2 | 0.007339162 |
| Upregulated gene | Pla2g4a | 0.011417536 |
| Upregulated gene | Plaur | 0.002058136 |
| Upregulated gene | Nfe2l2 | 0.011040186 |
| Upregulated gene | Inpp5d | 0.003700084 |
| Upregulated gene | Ltbr | 0.002288162 |
| Upregulated gene | Zfp36 | 0.017377335 |
| Upregulated gene | Tgfb1 | 0.002357384 |
| Upregulated gene | Icam1 | 0.007105915 |
| Upregulated gene | Ptx3 | 0.000782241 |
| Upregulated gene | Cd97 | 0.008408461 |
| Upregulated gene | Hck | 0.000782241 |
| Upregulated gene | Akirin2 | 0.032819403 |
| Upregulated gene | Myd88 | 0.007475459 |
| Upregulated gene | Stat6 | 0.004542052 |
| Upregulated gene | Adam17 | 0.005381708 |
| Upregulated gene | Mrc1 | 0.036233872 |
| Upregulated gene | Nras | 0.00796831 |
| Upregulated gene | Ifngr1 | 0.001115311 |
| Upregulated gene | Bst2 | 0.007105915 |
| Upregulated gene | Csf1 | 0.003353618 |
| Upregulated gene | Eps8 | 0.03945457 |
| Upregulated gene | Rela | 0.005220225 |
| Upregulated gene | Trim30a | 0.002326275 |
| Upregulated gene | H2-Ab1 | 0.002598036 |
| Upregulated gene | Tnfaip8l2 | 0.014323995 |
| Upregulated gene | Rac2 | 0.007487865 |
| Upregulated gene | Myc | 0.021610519 |
| Upregulated gene | Mapkapk2 | 0.021387184 |
| Upregulated gene | Cxcl16 | 0.014323995 |
| Upregulated gene | Havcr2 | 0.001264725 |
| Upregulated gene | Kitl | 0.011656316 |
| Upregulated gene | Pmaip1 | 0.007487865 |
| Upregulated gene | Btk | 0.007487865 |
| Upregulated gene | Gbp2 | 0.002288162 |
| Upregulated gene | Naip2 | 0.001749502 |
| Upregulated gene | Ntn1 | 0.010432823 |
| Upregulated gene | Bcl10 | 0.003590212 |
| Upregulated gene | Ehmt2 | 0.044373344 |
| Upregulated gene | Psmb8 | 0.001909708 |
| Upregulated gene | Trim25 | 0.007105915 |
| Upregulated gene | Clec5a | 0.043049481 |
| Upregulated gene | Bcl3 | 0.0359699 |
| Upregulated gene | Nfil3 | 0.020191036 |
| Upregulated gene | Anpep | 0.032367705 |
| Upregulated gene | Rb1 | 0.006462915 |
| Upregulated gene | Prkcd | 0.041136112 |
| Upregulated gene | Apobec3 | 0.002089251 |
| Upregulated gene | Ap3b1 | 0.018311239 |
| Upregulated gene | Tmem173 | 0.008408461 |
| Upregulated gene | Lcp2 | 0.001287248 |
| Upregulated gene | Nfkb1 | 0.021325691 |
| Upregulated gene | Tlr1 | 0.00745514 |
| Upregulated gene | Lair1 | 0.007105915 |
| Upregulated gene | Naip5 | 0.004542052 |
| Upregulated gene | Hs2st1 | 0.011948198 |
| Upregulated gene | Casp1 | 0.004395361 |
| Upregulated gene | Lst1 | 0.003570419 |
| Upregulated gene | Klf4 | 0.024693556 |
| Upregulated gene | Ly96 | 0.004681676 |
| Upregulated gene | Il4ra | 0.014323995 |
| Upregulated gene | Cd180 | 0.003051297 |
| Upregulated gene | AI607873 | 0.009057416 |
| Upregulated gene | Ddx41 | 0.004106526 |
| Upregulated gene | Rarres2 | 0.003055086 |
| Upregulated gene | Ctnnd1 | 0.049419821 |
| Upregulated gene | Ifih1 | 0.032912428 |
| Upregulated gene | Casp7 | 0.006293446 |
| Upregulated gene | Zc3hav1 | 0.01235233 |
| Upregulated gene | Eif4e | 0.028095723 |
| Upregulated gene | Ddx21 | 0.038932628 |
| Upregulated gene | P2rx7 | 0.003829014 |
| Upregulated gene | Pstpip1 | 0.023871891 |
| Upregulated gene | Cr1l | 0.003506048 |
| Upregulated gene | Jak3 | 0.031328017 |
| Upregulated gene | Irak2 | 0.021552863 |
| Upregulated gene | Fcna | 0.009108058 |
| Upregulated gene | Ifit2 | 0.005744866 |
| Upregulated gene | Ripk3 | 0.01295036 |
| Upregulated gene | Rab8a | 0.018563896 |
| Upregulated gene | Mavs | 0.016790309 |
| Upregulated gene | Mr1 | 0.011730455 |
| Upregulated gene | Was | 0.007487865 |
| Upregulated gene | Cxcl10 | 0.009645466 |
| Upregulated gene | Daglb | 0.046817814 |
| Upregulated gene | Yy1 | 0.013114214 |
| Upregulated gene | Elf1 | 0.007594054 |
| Upregulated gene | Tlr7 | 0.005655825 |
| Upregulated gene | Sirt1 | 0.01420704 |
| Upregulated gene | Syk | 0.013859999 |
| Upregulated gene | Akt1 | 0.040820317 |
| Upregulated gene | Irf5 | 0.005197453 |
| Upregulated gene | Fgr | 0.021789458 |
| Upregulated gene | Ifnar1 | 0.031967239 |
| Upregulated gene | 5-Mar | 0.027069265 |
| Upregulated gene | Cd200r1 | 0.011807568 |
| Upregulated gene | Trim21 | 0.008229518 |
| Upregulated gene | Pik3cg | 0.005030411 |
| Upregulated gene | Oasl2 | 0.040201454 |
| Upregulated gene | Orai1 | 0.011417536 |
| Upregulated gene | Polr3c | 0.023093017 |
| Upregulated gene | Trim12c | 0.007105915 |
| Upregulated gene | Stat1 | 0.044941392 |
| Upregulated gene | Pyhin1 | 0.00789307 |
| Upregulated gene | Jak2 | 0.03151574 |
| Upregulated gene | Plscr1 | 0.01420704 |
| Upregulated gene | Nlrc5 | 0.014154502 |
| Upregulated gene | Cflar | 0.02199542 |
| Upregulated gene | Cd1d1 | 0.006554163 |
| Upregulated gene | Tirap | 0.032039036 |
| Upregulated gene | Ikbke | 0.025889125 |
| Upregulated gene | Gbp7 | 0.032874036 |
| Upregulated gene | Birc3 | 0.036188648 |
| Upregulated gene | Irak4 | 0.008718183 |
| Upregulated gene | Daxx | 0.049419821 |
| Upregulated gene | Tank | 0.012391093 |
| Upregulated gene | Rhbdf2 | 0.034184418 |
| Upregulated gene | Rnf135 | 0.046817814 |
| Upregulated gene | Tlr4 | 0.01295036 |
| Upregulated gene | Ikbkb | 0.021183715 |
| Upregulated gene | Tnfaip3 | 0.047406422 |
| Upregulated gene | Il1rl2 | 0.014323995 |
| Upregulated gene | Nr1h3 | 0.023476098 |
| Upregulated gene | Traf2 | 0.039961308 |
| Upregulated gene | Il1r2 | 0.045507516 |
| Upregulated gene | Clec4e | 0.011417536 |
| Upregulated gene | Akna | 0.030359434 |
| Upregulated gene | Tlr6 | 0.01295036 |
| Upregulated gene | Zbp1 | 0.044373344 |
| Upregulated gene | Mapk14 | 0.025889125 |
| Upregulated gene | Trib3 | 0.035906418 |
| Upregulated gene | Map3k8 | 0.035906418 |
| Upregulated gene | Trim15 | 0.039010493 |
| Upregulated gene | Iigp1 | 0.027503695 |
| Upregulated gene | Trem1 | 0.028256161 |
| Upregulated gene | Clec4a2 | 0.026942303 |
| Upregulated gene | Padi4 | 0.032819403 |
| Upregulated gene | Zc3h12a | 0.03151574 |
| Upregulated gene | Akap10 | 0.0266465 |
| Upregulated gene | Cd40 | 0.041740943 |
| Upregulated gene | Relb | 0.034345006 |
| Upregulated gene | Trim14 | 0.049427038 |
| Downregulated gene | Sarm1 | 0.020451388 |
| Downregulated gene | Chat | 0.030723457 |
| Downregulated gene | Cd55 | 0.021552863 |
| Downregulated gene | Bcl11b | 0.038932628 |
| Downregulated gene | Polr3g | 0.034184418 |
| Downregulated gene | Camk2a | 0.007996557 |
| Downregulated gene | Rgmb | 0.011417536 |
| Downregulated gene | Sema3a | 0.02052641 |
| Downregulated gene | Nfatc2 | 0.008074283 |
| Downregulated gene | Zfpm2 | 0.007487865 |
| Downregulated gene | Camkk2 | 0.03151574 |
| Downregulated gene | Ip6k1 | 0.016131122 |
| Downregulated gene | Dusp16 | 0.007996557 |
| Downregulated gene | Clock | 0.021865155 |
| Downregulated gene | Drd2 | 0.005220225 |
| Downregulated gene | Cyld | 0.01891492 |
| Downregulated gene | Tecpr1 | 0.012194766 |
| Downregulated gene | 1700021K19Rik | 0.026942303 |
| Downregulated gene | Arntl | 0.011656316 |
| Downregulated gene | Tbkbp1 | 0.024027408 |
| Downregulated gene | Vldlr | 0.022066725 |
| Downregulated gene | Rora | 0.020838659 |
| Downregulated gene | Trib2 | 0.007487865 |
| Downregulated gene | Map4k2 | 0.002716506 |
| Downregulated gene | Ecsit | 0.007487865 |
| Downregulated gene | Trpm2 | 0.006380454 |
| Downregulated gene | Ccdc88a | 0.041740943 |
| Downregulated gene | Pik3c3 | 0.042602412 |
| Downregulated gene | Vegfa | 0.010461632 |
| Downregulated gene | Gpsm1 | 0.009950239 |
| Downregulated gene | Mapk8 | 0.009108058 |
| Downregulated gene | Mfn2 | 0.029122677 |
| Downregulated gene | Traf3 | 0.019199126 |
| Downregulated gene | Stub1 | 0.014323995 |
| Downregulated gene | Mtor | 0.001287248 |
| Downregulated gene | Adar | 0.004106526 |
| Downregulated gene | Dlk1 | 0.004542052 |
| Downregulated gene | Trim13 | 0.009057416 |
| Downregulated gene | Mfn1 | 0.01567626 |
| Downregulated gene | Edil3 | 0.01379208 |
| Downregulated gene | Srebf2 | 0.032819403 |
| Downregulated gene | Gsk3a | 0.023414833 |
| Downregulated gene | Mapk1 | 0.038454435 |
| Downregulated gene | Ptch1 | 0.002999934 |
| Downregulated gene | Ppargc1a | 0.003291103 |
| Downregulated gene | Pin1 | 0.00673072 |
| Downregulated gene | Snca | 0.035906418 |
| Downregulated gene | Ubqln1 | 0.003487368 |
| Downregulated gene | Ppargc1b | 0.001158354 |
| Downregulated gene | Dhcr7 | 0.00468656 |
| Downregulated gene | Idi1 | 0.007695387 |
| Downregulated gene | Trim32 | 0.003010897 |
| Downregulated gene | Pura | 0.02355479 |
| Downregulated gene | Rheb | 0.034424464 |
| Downregulated gene | Ppp3r1 | 0.00673072 |
| Downregulated gene | Fbxo9 | 0.006852577 |
| Downregulated gene | Stmn1 | 0.035006319 |
| Downregulated gene | Chga | 0.004681676 |
| Downregulated gene | Pacsin1 | 0.001035278 |

Supplemental Table 1E Differentially expressed innate immune-related genes at 7 days after SCI

| Gene type | Gene | adj.P.Val |
| --- | --- | --- |
| Upregulated gene | Spp1 | 0.008299313 |
| Upregulated gene | C1qc | 0.004897688 |
| Upregulated gene | C1qa | 0.006213144 |
| Upregulated gene | Ctss | 0.002959439 |
| Upregulated gene | Lgals3 | 0.009195907 |
| Upregulated gene | Tyrobp | 0.006213144 |
| Upregulated gene | Grn | 0.010636663 |
| Upregulated gene | C1qb | 0.002959439 |
| Upregulated gene | Ly86 | 0.012556271 |
| Upregulated gene | Ctsb | 0.008299313 |
| Upregulated gene | Clec7a | 0.008299313 |
| Upregulated gene | Csf1r | 0.009608187 |
| Upregulated gene | Cyba | 0.012981533 |
| Upregulated gene | Lgmn | 0.01744229 |
| Upregulated gene | C3 | 0.030765349 |
| Upregulated gene | Abca1 | 0.00477898 |
| Upregulated gene | Itgb2 | 0.018443791 |
| Upregulated gene | Gabarap | 0.017145227 |
| Upregulated gene | C3ar1 | 0.020053193 |
| Upregulated gene | Trem2 | 0.009608187 |
| Upregulated gene | Unc93b1 | 0.008299313 |
| Upregulated gene | Cdc42 | 0.0330186 |
| Upregulated gene | Cd5l | 0.041923624 |
| Upregulated gene | Lgals9 | 0.022653852 |
| Upregulated gene | Cd48 | 0.012826377 |
| Upregulated gene | Il33 | 0.040642588 |
| Upregulated gene | Ctnnb1 | 0.025146997 |
| Upregulated gene | Sirpa | 0.009608187 |
| Upregulated gene | Ifit3 | 0.008299313 |
| Upregulated gene | Cd36 | 0.024783066 |
| Upregulated gene | Slc11a1 | 0.039647583 |
| Upregulated gene | Ppp1ca | 0.033352435 |
| Upregulated gene | S100a10 | 0.015325549 |
| Upregulated gene | Cebpa | 0.017959727 |
| Upregulated gene | Tnfrsf1a | 0.040642588 |
| Upregulated gene | Srebf1 | 0.024180354 |
| Upregulated gene | Hmox1 | 0.032838682 |
| Upregulated gene | Nfkbia | 0.043872547 |
| Upregulated gene | Txnip | 0.017056203 |
| Upregulated gene | Mertk | 0.008745718 |
| Upregulated gene | Cd14 | 0.009195907 |
| Upregulated gene | Cfh | 0.028726789 |
| Upregulated gene | Ybx1 | 0.028726789 |
| Upregulated gene | Atf3 | 0.015325549 |
| Upregulated gene | Irf8 | 0.015115701 |
| Upregulated gene | Tlr2 | 0.006213144 |
| Upregulated gene | Pros1 | 0.007985342 |
| Upregulated gene | Xbp1 | 0.042971713 |
| Upregulated gene | Tlr13 | 0.016863 |
| Upregulated gene | Axl | 0.01744229 |
| Upregulated gene | Gbp2 | 0.023264032 |
| Upregulated gene | Bst2 | 0.047260772 |
| Upregulated gene | Oasl2 | 0.03830577 |
| Upregulated gene | Cxcr4 | 0.031400737 |
| Upregulated gene | Nfe2l2 | 0.00477898 |
| Upregulated gene | Fcgr1 | 0.013343311 |
| Upregulated gene | Itgax | 0.00477898 |
| Upregulated gene | Cxcl16 | 0.039130485 |
| Upregulated gene | Trim30a | 0.018129019 |
| Upregulated gene | Ptpn6 | 0.00477898 |
| Upregulated gene | Casp8 | 0.024162064 |
| Upregulated gene | Hif1a | 0.024162064 |
| Upregulated gene | Hdac1 | 0.00477898 |
| Upregulated gene | H2-Aa | 0.015115701 |
| Upregulated gene | Cebpd | 0.009195907 |
| Upregulated gene | Abcg1 | 0.033352435 |
| Upregulated gene | Cebpb | 0.012556271 |
| Upregulated gene | Clec4d | 0.024162064 |
| Upregulated gene | Tnfaip8l2 | 0.009195907 |
| Upregulated gene | Pycard | 0.022177072 |
| Upregulated gene | Plcg2 | 0.027477081 |
| Upregulated gene | Ifngr1 | 0.030383467 |
| Upregulated gene | Gas6 | 0.046963083 |
| Upregulated gene | Ifit1 | 0.031315794 |
| Upregulated gene | Adam17 | 0.041923624 |
| Upregulated gene | Akirin2 | 0.039130485 |
| Upregulated gene | Cfp | 0.036053939 |
| Upregulated gene | Hck | 0.009195907 |
| Upregulated gene | Inpp5d | 0.024162064 |
| Upregulated gene | Cxcl10 | 0.049587201 |
| Upregulated gene | Itgam | 0.024180354 |
| Upregulated gene | Stat6 | 0.024880427 |
| Upregulated gene | Arf6 | 0.015325549 |
| Upregulated gene | Havcr2 | 0.041923624 |
| Upregulated gene | Cd300a | 0.020053193 |
| Upregulated gene | Stat1 | 0.012556271 |
| Upregulated gene | Cd300lf | 0.023063527 |
| Upregulated gene | Psmb8 | 0.024162064 |
| Upregulated gene | Gnai3 | 0.027966808 |
| Upregulated gene | Ifih1 | 0.026077036 |
| Upregulated gene | Lair1 | 0.008299313 |
| Upregulated gene | Ifit2 | 0.00477898 |
| Upregulated gene | H2-Ab1 | 0.010636663 |
| Upregulated gene | C5ar1 | 0.03580128 |
| Upregulated gene | Igtp | 0.039091555 |
| Upregulated gene | Rac2 | 0.028726789 |
| Upregulated gene | Csf1 | 0.024162064 |
| Upregulated gene | Myd88 | 0.03045303 |
| Upregulated gene | Msr1 | 0.024162064 |
| Upregulated gene | Ehmt2 | 0.025146997 |
| Upregulated gene | Clec4n | 0.040253899 |
| Upregulated gene | Ptx3 | 0.008299313 |
| Upregulated gene | Prkcd | 0.016863 |
| Upregulated gene | Irgm1 | 0.009608187 |
| Upregulated gene | Naip5 | 0.010250443 |
| Upregulated gene | Mrc1 | 0.012556271 |
| Upregulated gene | Iigp1 | 0.017987766 |
| Upregulated gene | Clec5a | 0.025503903 |
| Upregulated gene | Serpine1 | 0.047260772 |
| Upregulated gene | Eif4ebp1 | 0.022653852 |
| Upregulated gene | Rarres2 | 0.010636663 |
| Upregulated gene | Lcp2 | 0.017987766 |
| Upregulated gene | Apobec3 | 0.008299313 |
| Upregulated gene | Btk | 0.024162064 |
| Upregulated gene | Bcl10 | 0.024162064 |
| Upregulated gene | Naip2 | 0.016863 |
| Upregulated gene | Gbp6 | 0.025146997 |
| Upregulated gene | Tmem173 | 0.048945179 |
| Upregulated gene | Plaur | 0.012826377 |
| Upregulated gene | Cd180 | 0.032350222 |
| Upregulated gene | Nampt | 0.012556271 |
| Upregulated gene | Daglb | 0.01744229 |
| Upregulated gene | Tlr7 | 0.024783066 |
| Upregulated gene | Lst1 | 0.012556271 |
| Upregulated gene | Casp1 | 0.010250443 |
| Upregulated gene | Cd86 | 0.011255912 |
| Upregulated gene | Myc | 0.033195496 |
| Upregulated gene | Ccl2 | 0.009195907 |
| Upregulated gene | Trim21 | 0.040253899 |
| Upregulated gene | Tlr1 | 0.009608187 |
| Upregulated gene | Zc3hav1 | 0.024783066 |
| Upregulated gene | Rb1 | 0.045729135 |
| Upregulated gene | Mr1 | 0.032610767 |
| Upregulated gene | P2rx7 | 0.049319218 |
| Upregulated gene | Trim12c | 0.042079324 |
| Upregulated gene | Ly96 | 0.008299313 |
| Upregulated gene | Syk | 0.028726789 |
| Upregulated gene | Was | 0.024162064 |
| Upregulated gene | Jak3 | 0.040721501 |
| Upregulated gene | Siglech | 0.046963083 |
| Upregulated gene | Gbp7 | 0.031315794 |
| Upregulated gene | Pyhin1 | 0.017145227 |
| Upregulated gene | Herc6 | 0.009608187 |
| Upregulated gene | Pten | 0.043063176 |
| Upregulated gene | Irf5 | 0.010636663 |
| Upregulated gene | Bcl3 | 0.020436269 |
| Upregulated gene | Il1r1 | 0.015325549 |
| Upregulated gene | Ddx41 | 0.030640172 |
| Upregulated gene | Slc15a4 | 0.027966808 |
| Upregulated gene | Fgr | 0.040253899 |
| Upregulated gene | Klf4 | 0.027966808 |
| Upregulated gene | Cd200r1 | 0.018522789 |
| Upregulated gene | Pik3ap1 | 0.023063527 |
| Upregulated gene | Ddx21 | 0.025503903 |
| Upregulated gene | Nlrc5 | 0.047260772 |
| Upregulated gene | Pik3cg | 0.049319218 |
| Upregulated gene | Zbp1 | 0.033018437 |
| Upregulated gene | Pstpip1 | 0.024162064 |
| Upregulated gene | Tlr8 | 0.022653852 |
| Upregulated gene | Elf1 | 0.015325549 |
| Upregulated gene | Il4ra | 0.041086736 |
| Upregulated gene | Foxo3 | 0.033352435 |
| Upregulated gene | Mknk1 | 0.0330186 |
| Upregulated gene | Cr1l | 0.045297536 |
| Upregulated gene | Eif4e | 0.015454958 |
| Upregulated gene | Irak4 | 0.033352435 |
| Upregulated gene | Cflar | 0.027966808 |
| Upregulated gene | Trim14 | 0.03959792 |
| Upregulated gene | Tlr4 | 0.030084519 |
| Upregulated gene | Il1rl2 | 0.024162064 |
| Upregulated gene | Prdm1 | 0.036942076 |
| Upregulated gene | Mapk14 | 0.024162064 |
| Upregulated gene | Scaf11 | 0.028726789 |
| Upregulated gene | Map3k8 | 0.047485874 |
| Upregulated gene | Tlr6 | 0.040253899 |
| Upregulated gene | Scarb1 | 0.045796655 |
| Downregulated gene | Cd55 | 0.039130485 |
| Downregulated gene | Sema3a | 0.028726789 |
| Downregulated gene | Bcl11b | 0.031156448 |
| Downregulated gene | Nfatc2 | 0.041923624 |
| Downregulated gene | Polr3a | 0.049861435 |
| Downregulated gene | Lrrfip2 | 0.047789266 |
| Downregulated gene | Mmp9 | 0.039130485 |
| Downregulated gene | Drd2 | 0.028726789 |
| Downregulated gene | Camkk2 | 0.033195496 |
| Downregulated gene | Trpm2 | 0.020436269 |
| Downregulated gene | Dab2ip | 0.044565036 |
| Downregulated gene | Ecsit | 0.024168853 |
| Downregulated gene | Map4k2 | 0.025146997 |
| Downregulated gene | Trib2 | 0.033352435 |
| Downregulated gene | Trim59 | 0.009608187 |
| Downregulated gene | Tbkbp1 | 0.039130485 |
| Downregulated gene | Arntl | 0.023949327 |
| Downregulated gene | Adar | 0.0261058 |
| Downregulated gene | Clock | 0.024162064 |
| Downregulated gene | Mapk8 | 0.017987766 |
| Downregulated gene | Gsk3a | 0.041086736 |
| Downregulated gene | Vldlr | 0.028726789 |
| Downregulated gene | Mtor | 0.015054711 |
| Downregulated gene | Dlk1 | 0.015115701 |
| Downregulated gene | Trim13 | 0.043063176 |
| Downregulated gene | Vegfa | 0.024783066 |
| Downregulated gene | Traf3 | 0.039802379 |
| Downregulated gene | Ppargc1a | 0.02116014 |
| Downregulated gene | Ubqln1 | 0.028726789 |
| Downregulated gene | Srebf2 | 0.039251743 |
| Downregulated gene | Ptch1 | 0.008745718 |
| Downregulated gene | Ppargc1b | 0.006213144 |
| Downregulated gene | Pin1 | 0.022177072 |
| Downregulated gene | Ppp3r1 | 0.039099478 |
| Downregulated gene | Dhcr7 | 0.012981533 |
| Downregulated gene | Trim32 | 0.010636663 |
| Downregulated gene | Ddx1 | 0.015325549 |
| Downregulated gene | Fbxo9 | 0.041923624 |
| Downregulated gene | Rheb | 0.039130485 |
| Downregulated gene | Idi1 | 0.012826377 |
| Downregulated gene | Chga | 0.024257389 |
| Downregulated gene | Stmn1 | 0.042971713 |
| Downregulated gene | Pacsin1 | 0.009195907 |

Supplemental Table 1F Differentially expressed innate immune-related genes at 28 days after SCI

| Gene type | Gene | adj.P.Val |
| --- | --- | --- |
| Upregulated gene | Ctss | 0.000333729 |
| Upregulated gene | C1qa | 0.000333729 |
| Upregulated gene | Tyrobp | 0.000898809 |
| Upregulated gene | C1qc | 0.002843831 |
| Upregulated gene | Spp1 | 0.000539859 |
| Upregulated gene | C3 | 0.000539859 |
| Upregulated gene | Lgals3 | 0.000333729 |
| Upregulated gene | C1qb | 0.000282683 |
| Upregulated gene | Grn | 0.000886449 |
| Upregulated gene | Ctsb | 0.000886449 |
| Upregulated gene | Clec7a | 0.001616167 |
| Upregulated gene | Csf1r | 0.000430757 |
| Upregulated gene | Ly86 | 0.000539859 |
| Upregulated gene | Lcn2 | 0.048524688 |
| Upregulated gene | Cyba | 0.002104955 |
| Upregulated gene | Serping1 | 0.002187078 |
| Upregulated gene | Abca1 | 0.001880291 |
| Upregulated gene | Itgb2 | 0.002795887 |
| Upregulated gene | Dcn | 0.000539859 |
| Upregulated gene | Pltp | 0.008418991 |
| Upregulated gene | Ifitm2 | 0.001065196 |
| Upregulated gene | Gabarap | 0.009635503 |
| Upregulated gene | Il33 | 0.001804189 |
| Upregulated gene | Ifit3 | 0.027250603 |
| Upregulated gene | Lgmn | 0.039999675 |
| Upregulated gene | S100a10 | 0.002775583 |
| Upregulated gene | Cd48 | 0.000333729 |
| Upregulated gene | Hsp90b1 | 0.036060057 |
| Upregulated gene | Trem2 | 0.007966553 |
| Upregulated gene | Nfkbia | 0.003743852 |
| Upregulated gene | Cdc42 | 0.008418991 |
| Upregulated gene | Srebf1 | 0.030484103 |
| Upregulated gene | Slc11a1 | 0.000886449 |
| Upregulated gene | C3ar1 | 0.024096455 |
| Upregulated gene | Cd14 | 0.002625894 |
| Upregulated gene | Igf1 | 0.024347604 |
| Upregulated gene | Ppp1ca | 0.029232539 |
| Upregulated gene | Unc93b1 | 0.000430757 |
| Upregulated gene | Itgax | 0.002565405 |
| Upregulated gene | Xbp1 | 0.048395964 |
| Upregulated gene | Tlr2 | 0.000539859 |
| Upregulated gene | Txnip | 0.010000333 |
| Upregulated gene | Lgals9 | 0.000539859 |
| Upregulated gene | Anxa2 | 0.001880291 |
| Upregulated gene | Pros1 | 0.001913302 |
| Upregulated gene | Gbp2 | 0.023873158 |
| Upregulated gene | Sirpa | 0.008095782 |
| Upregulated gene | Cebpa | 0.007286347 |
| Upregulated gene | Mertk | 0.010392905 |
| Upregulated gene | Tnfrsf1a | 0.002659246 |
| Upregulated gene | Cd5l | 0.022990078 |
| Upregulated gene | Nfe2l2 | 0.000964127 |
| Upregulated gene | Cfh | 0.047025528 |
| Upregulated gene | Rac1 | 0.021412393 |
| Upregulated gene | Ybx1 | 0.017966129 |
| Upregulated gene | Axl | 0.006349959 |
| Upregulated gene | Irf8 | 0.002632726 |
| Upregulated gene | Trim30a | 0.022990078 |
| Upregulated gene | Hif1a | 0.009635503 |
| Upregulated gene | H2-Aa | 0.004684471 |
| Upregulated gene | Cebpd | 0.012845009 |
| Upregulated gene | Cxcl16 | 0.000333729 |
| Upregulated gene | Cxcl10 | 0.047025528 |
| Upregulated gene | Lbp | 0.007055629 |
| Upregulated gene | Stat3 | 0.012110965 |
| Upregulated gene | Ptpn6 | 0.003855976 |
| Upregulated gene | Gnai2 | 0.04725364 |
| Upregulated gene | Abcg1 | 0.010000333 |
| Upregulated gene | Fcgr1 | 0.002612485 |
| Upregulated gene | Atf3 | 0.002632726 |
| Upregulated gene | Cebpb | 0.003257329 |
| Upregulated gene | Anxa1 | 0.033920795 |
| Upregulated gene | Igtp | 0.045724396 |
| Upregulated gene | Ifngr1 | 0.010504192 |
| Upregulated gene | Bst2 | 0.023873158 |
| Upregulated gene | Icam1 | 0.002795887 |
| Upregulated gene | Tmed7 | 0.013596034 |
| Upregulated gene | Cxcr4 | 0.004355179 |
| Upregulated gene | Pycard | 0.003271464 |
| Upregulated gene | Akirin2 | 0.027769606 |
| Upregulated gene | Tnfaip8l2 | 0.001553524 |
| Upregulated gene | Tlr13 | 0.000886449 |
| Upregulated gene | Mfge8 | 0.003056588 |
| Upregulated gene | Psmb8 | 0.002795887 |
| Upregulated gene | Stat1 | 0.035846625 |
| Upregulated gene | Oas1a | 0.004355179 |
| Upregulated gene | Hdac1 | 0.014114557 |
| Upregulated gene | Plcg2 | 0.002632726 |
| Upregulated gene | Hck | 0.002104955 |
| Upregulated gene | Rarres2 | 0.003486406 |
| Upregulated gene | Itgam | 0.003403822 |
| Upregulated gene | H2-Ab1 | 0.003486406 |
| Upregulated gene | Gnai3 | 0.007651152 |
| Upregulated gene | Gbp6 | 0.030484103 |
| Upregulated gene | Ifih1 | 0.015481873 |
| Upregulated gene | Casp8 | 0.010000333 |
| Upregulated gene | Ltbr | 0.003976279 |
| Upregulated gene | Rela | 0.013596034 |
| Upregulated gene | C1ra | 0.020698392 |
| Upregulated gene | Stat6 | 0.008418991 |
| Upregulated gene | Ptx3 | 0.013481319 |
| Upregulated gene | Prkcd | 0.001880291 |
| Upregulated gene | Arf6 | 0.042382895 |
| Upregulated gene | Csf1 | 0.018527767 |
| Upregulated gene | Clec4d | 0.003889435 |
| Upregulated gene | Havcr2 | 0.007160548 |
| Upregulated gene | Lair1 | 0.001913302 |
| Upregulated gene | Rb1 | 0.007966553 |
| Upregulated gene | Inpp5d | 0.003271464 |
| Upregulated gene | Adam17 | 0.014736459 |
| Upregulated gene | Tgfb1 | 0.005700314 |
| Upregulated gene | Anpep | 0.010000333 |
| Upregulated gene | Zfp36 | 0.023873158 |
| Upregulated gene | Naip2 | 0.015547277 |
| Upregulated gene | Ccl2 | 0.033537626 |
| Upregulated gene | Nfkb1 | 0.028333764 |
| Upregulated gene | Gbp7 | 0.023979809 |
| Upregulated gene | Myd88 | 0.030878462 |
| Upregulated gene | Cd86 | 0.007907248 |
| Upregulated gene | Cd300lf | 0.022990078 |
| Upregulated gene | Casp1 | 0.002261863 |
| Upregulated gene | Sirt1 | 0.041570493 |
| Upregulated gene | Angpt1 | 0.001720161 |
| Upregulated gene | Mapkapk2 | 0.001913302 |
| Upregulated gene | Nr3c1 | 0.036098657 |
| Upregulated gene | Mrc1 | 0.022990078 |
| Upregulated gene | Bcl10 | 0.003485728 |
| Upregulated gene | Clec5a | 0.002632726 |
| Upregulated gene | Tlr3 | 0.042230169 |
| Upregulated gene | Naip5 | 0.003316074 |
| Upregulated gene | Daglb | 0.049214379 |
| Upregulated gene | Lcp2 | 0.002795887 |
| Upregulated gene | Eif4ebp1 | 0.003271464 |
| Upregulated gene | Tlr1 | 0.004355179 |
| Upregulated gene | Serpine1 | 0.010391621 |
| Upregulated gene | Trim25 | 0.017302776 |
| Upregulated gene | C2 | 0.007651152 |
| Upregulated gene | Cd300a | 0.002625894 |
| Upregulated gene | Myc | 0.04198398 |
| Upregulated gene | Tlr7 | 0.021412393 |
| Upregulated gene | Btk | 0.043330822 |
| Upregulated gene | Trim21 | 0.045839693 |
| Upregulated gene | Irf1 | 0.023873158 |
| Upregulated gene | Tmem173 | 0.048923911 |
| Upregulated gene | AI607873 | 0.015403614 |
| Upregulated gene | Trim12c | 0.023873158 |
| Upregulated gene | Nampt | 0.004798156 |
| Upregulated gene | Apobec3 | 0.002664876 |
| Upregulated gene | Plaur | 0.023979809 |
| Upregulated gene | Lst1 | 0.00487139 |
| Upregulated gene | Rac2 | 0.027073119 |
| Upregulated gene | Zc3hav1 | 0.002843831 |
| Upregulated gene | Herc6 | 0.036098657 |
| Upregulated gene | Cr1l | 0.008182755 |
| Upregulated gene | Slc15a4 | 0.010418516 |
| Upregulated gene | Siglech | 0.023204312 |
| Upregulated gene | Was | 0.024347604 |
| Upregulated gene | Cd36 | 0.014299342 |
| Upregulated gene | Cd180 | 0.024770397 |
| Upregulated gene | Bcl3 | 0.026478193 |
| Upregulated gene | Fgr | 0.011266277 |
| Upregulated gene | Msr1 | 0.023391203 |
| Upregulated gene | Ly96 | 0.010504192 |
| Upregulated gene | Pyhin1 | 0.036098657 |
| Upregulated gene | Irf5 | 0.036256944 |
| Upregulated gene | Eif2ak2 | 0.023204312 |
| Upregulated gene | Peli1 | 0.013596034 |
| Upregulated gene | Pik3cg | 0.017966129 |
| Upregulated gene | C5ar1 | 0.017900126 |
| Upregulated gene | Jak2 | 0.029946436 |
| Upregulated gene | Tnfaip3 | 0.013596034 |
| Upregulated gene | P2rx7 | 0.014736459 |
| Upregulated gene | Il1r1 | 0.035379312 |
| Upregulated gene | Coch | 0.019532421 |
| Upregulated gene | Cflar | 0.042230169 |
| Upregulated gene | Pik3ap1 | 0.034928524 |
| Upregulated gene | Map3k8 | 0.031284687 |
| Upregulated gene | Cd200r1 | 0.019111862 |
| Upregulated gene | Oas1b | 0.023873158 |
| Upregulated gene | Clec4n | 0.037203512 |
| Upregulated gene | Syk | 0.04997643 |
| Upregulated gene | Akna | 0.037203512 |
| Upregulated gene | Irak4 | 0.033370815 |
| Upregulated gene | Tlr4 | 0.03109553 |
| Upregulated gene | Relb | 0.035379312 |
| Upregulated gene | Il1a | 0.031202919 |
| Downregulated gene | Sarm1 | 0.04200387 |
| Downregulated gene | Bcl11b | 0.045724396 |
| Downregulated gene | Zfpm2 | 0.047025528 |
| Downregulated gene | Nfatc2 | 0.026478193 |
| Downregulated gene | Zfpm1 | 0.023873158 |
| Downregulated gene | Ip6k1 | 0.029946436 |
| Downregulated gene | Trpm2 | 0.016109916 |
| Downregulated gene | Mmp9 | 0.030253822 |
| Downregulated gene | Lrrfip2 | 0.035379312 |
| Downregulated gene | Drd2 | 0.010504192 |
| Downregulated gene | Trib2 | 0.023873158 |
| Downregulated gene | Mapk8 | 0.039609251 |
| Downregulated gene | Dab2ip | 0.022990078 |
| Downregulated gene | Arntl | 0.047025528 |
| Downregulated gene | Vegfa | 0.042230169 |
| Downregulated gene | Tbkbp1 | 0.023204312 |
| Downregulated gene | Adar | 0.021412393 |
| Downregulated gene | Gpsm1 | 0.024223152 |
| Downregulated gene | Mfn1 | 0.02412796 |
| Downregulated gene | Mtor | 0.004401252 |
| Downregulated gene | Ubqln1 | 0.039609251 |
| Downregulated gene | Dlk1 | 0.013481319 |
| Downregulated gene | Ppargc1a | 0.037203512 |
| Downregulated gene | Ptch1 | 0.015152512 |
| Downregulated gene | Traf3 | 0.013481319 |
| Downregulated gene | Mapk1 | 0.024096455 |
| Downregulated gene | Ddx1 | 0.033370815 |
| Downregulated gene | Pin1 | 0.022354244 |
| Downregulated gene | Srebf2 | 0.035492668 |
| Downregulated gene | Trim32 | 0.022990078 |
| Downregulated gene | Ppp3r1 | 0.030705218 |
| Downregulated gene | Dhcr7 | 0.005700314 |
| Downregulated gene | Fbxo9 | 0.023873158 |
| Downregulated gene | Chga | 0.017352514 |
| Downregulated gene | Idi1 | 0.003316074 |
| Downregulated gene | Pacsin1 | 0.001834686 |

**Supplemental Table 2 Thirty GO terms with the lowest P value at each time point**

| Sub-groups | Category | GO terms | P | Genes |
| --- | --- | --- | --- | --- |
| 0.5h | GO_BP | regulation of monocyte chemotaxis | 6.96E-08 | Dusp1, Serpine1, Ccl2 |
|  |  | myeloid leukocyte migration | 4.18E-07 | Dusp1, Serpine1, Spp1, Ccl2 |
|  |  | leukocyte chemotaxis | 4.52E-07 | Dusp1, Serpine1, Spp1, Ccl2 |
|  |  | regulation of mononuclear cell migration | 5.23E-07 | Dusp1, Serpine1, Ccl2 |
|  |  | monocyte chemotaxis | 6.99E-07 | Dusp1, Serpine1, Ccl2 |
|  |  | cell chemotaxis | 1.81E-06 | Dusp1, Serpine1, Spp1, Ccl2 |
|  |  | mononuclear cell migration | 2.44E-06 | Dusp1, Serpine1, Ccl2 |
|  |  | leukocyte migration | 3.02E-06 | Dusp1, Serpine1, Spp1, Ccl2 |
|  |  | regulation of leukocyte chemotaxis | 5.86E-06 | Dusp1, Serpine1, Ccl2 |
|  |  | negative regulation of angiogenesis | 6.02E-06 | Klf4, Serpine1, Ccl2 |
|  | GO_CC | mRNA cap binding complex | 0.003408967 | Zfp36 |
|  |  | RNA cap binding complex | 0.003408967 | Zfp36 |
|  |  | RISC-loading complex | 0.003749303 | Zfp36 |
|  |  | DNA polymerase complex | 0.004769703 | Crcp |
|  |  | CCR4-NOT complex | 0.005789189 | Zfp36 |
|  |  | RNA polymerase III complex | 0.006128814 | Crcp |
|  |  | secretory granule | 0.006988268 | Crcp, Serpine1 |
|  |  | exosome (RNase complex) | 0.007825419 | Zfp36 |
|  |  | exoribonuclease complex | 0.007825419 | Zfp36 |
|  |  | endoribonuclease complex | 0.010196408 | Zfp36 |
|  | GO_MF | cytokine activity | 0.002405915 | Spp1, Ccl2 |
|  |  | RNA polymerase II sequence-specific DNA-binding transcription factor binding | 0.004220052 | Klf4 |
|  |  | MAP kinase tyrosine/serine/threonine phosphatase activity | 0.004571019 | Dusp1 |
|  |  | MAP kinase phosphatase activity | 0.005272627 | Dusp1 |
|  |  | RNA polymerase III activity | 0.005623269 | Crcp |
|  |  | phosphatidylinositol 3-kinase regulator activity | 0.006674546 | Klf4 |
|  |  | C-C chemokine binding | 0.008774183 | Zfp36 |
|  |  | mRNA 3'-UTR AU-rich region binding | 0.009822545 | Zfp36 |
|  |  | 14-3-3 protein binding | 0.010171783 | Zfp36 |
|  |  | AU-rich element binding | 0.010520913 | Zfp36 |
| 4h | GO_BP | positive regulation of cytokine production | 1.50E-26 | Atf4, Bcl10, Bcl3, C3ar1, Casp8, Ccl2, Cd14, Cebpb, Csf1r, Fgr, Il1a, Il1r1, Il33, Il4ra, Il6, Jak2, Mapk14, Mapkapk2, Myd88, Nr4a3, Rela, Stat3, Tgfb1, Tlr2, Tnfrsf1a, Tnfsf9, Tyrobp |
|  |  | cellular response to molecule of bacterial origin | 2.70E-22 | Bcl10, Ccl2, Cd14, Cd86, Cebpb, Cxcl1, Cxcl10, Cxcl16, Gbp2, Il6, Jak2, Mapk14, Myd88, Nfkb1, Nfkbia, Rela, Tgfb1, Tlr2, Tnfaip3, Zc3h12a, Zfp36 |
|  |  | cellular response to biotic stimulus | 1.34E-21 | Bcl10, Ccl2, Cd14, Cd86, Cebpb, Cxcl1, Cxcl10, Cxcl16, Gbp2, Il6, Jak2, Mapk14, Myd88, Nfkb1, Nfkbia, Rela, Tgfb1, Tlr2, Tnfaip3, Zc3h12a, Zfp36 |
|  |  | response to molecule of bacterial origin | 2.68E-21 | Bcl10, Ccl2, Cd14, Cd86, Cebpb, Cxcl1, Cxcl10, Cxcl16, Gbp2, Il6, Jak2, Mapk14, Mapkapk2, Myd88, Nfkb1, Nfkbia, Rela, Tgfb1, Tlr2, Tnfaip3, Zc3h12a, Zfp36 |
|  |  | cellular response to lipopolysaccharide | 4.59E-21 | Bcl10, Ccl2, Cd14, Cd86, Cebpb, Cxcl1, Cxcl10, Cxcl16, Gbp2, Il6, Jak2, Mapk14, Myd88, Nfkb1, Nfkbia, Rela, Tgfb1, Tnfaip3, Zc3h12a, Zfp36 |
|  |  | regulation of cytokine biosynthetic process | 2.26E-20 | Bcl10, Bcl3, Cebpb, Il1a, Il6, Jak2, Mapkapk2, Myd88, Nfkb1, Rela, Stat3, Tlr2, Trib2, Tyrobp, Zfp36 |
|  |  | response to lipopolysaccharide | 2.33E-20 | Bcl10, Ccl2, Cd14, Cd86, Cebpb, Cxcl1, Cxcl10, Cxcl16, Gbp2, Il6, Jak2, Mapk14, Mapkapk2, Myd88, Nfkb1, Nfkbia, Rela, Tgfb1, Tnfaip3, Zc3h12a, Zfp36 |
|  |  | cytokine biosynthetic process | 1.17E-19 | Bcl10, Bcl3, Cebpb, Il1a, Il6, Jak2, Mapkapk2, Myd88, Nfkb1, Rela, Stat3, Tlr2, Trib2, Tyrobp, Zfp36 |
|  |  | cytokine metabolic process | 1.94E-19 | Bcl10, Bcl3, Cebpb, Il1a, Il6, Jak2, Mapkapk2, Myd88, Nfkb1, Rela, Stat3, Tlr2, Trib2, Tyrobp, Zfp36 |
|  |  | regulation of DNA-binding transcription factor activity | 3.76E-18 | Bcl10, Cflar, Hmox1, Icam1, Id2, Il6, Jak2, Myd88, Nfkbia, Nlrc5, Ppargc1a, Ptch1, Rela, Stat3, Tgfb1, Tlr2, Tnfaip3, Trib2, Trim13, Zc3h12a |
|  | GO_CC | membrane raft | 2.21E-11 | Anxa2, Bcl10, Birc3, Casp8, Cd14, Cflar, Ctsb, Hmox1, Icam1, Jak2, Ptch1, S100a10, Tlr2, Tnfrsf1a |
|  |  | membrane microdomain | 2.30E-11 | Anxa2, Bcl10, Birc3, Casp8, Cd14, Cflar, Ctsb, Hmox1, Icam1, Jak2, Ptch1, S100a10, Tlr2, Tnfrsf1a |
|  |  | membrane region | 3.53E-11 | Anxa2, Bcl10, Birc3, Casp8, Cd14, Cflar, Ctsb, Hmox1, Icam1, Jak2, Ptch1, S100a10, Tlr2, Tnfrsf1a |
|  |  | extracellular organelle | 4.30E-05 | Anxa2, Cd86, Gbp2, Icam1, Iigp1 |
|  |  | immunological synapse | 0.000296436 | Bcl10, Icam1, Lgals3 |
|  |  | caveola | 0.000304017 | Ctsb, Hmox1, Jak2, Ptch1 |
|  |  | receptor complex | 0.000398422 | Cd14, Csf1r, Il4ra, Il6, Tlr2, Tnfrsf1a, Vldlr |
|  |  | autolysosome | 0.000620379 | Irgm1, Sqstm1 |
|  |  | symbiont-containing vacuole membrane | 0.000742806 | Gbp2, Iigp1 |
|  |  | plasma membrane raft | 0.000839903 | Ctsb, Hmox1, Jak2, Ptch1 |
|  | GO_MF | cytokine receptor binding | 4.91E-13 | Casp8, Ccl2, Cflar, Csf1, Cxcl1, Cxcl10, Cxcl16, Il1a, Il6, Jak2, Myd88, Stat3, Tgfb1, Tnfsf9, Tollip |
|  |  | cytokine activity | 4.34E-09 | Ccl2, Csf1, Cxcl1, Cxcl10, Cxcl16, Il1a, Il33, Il6, Tgfb1, Tnfsf9 |
|  |  | ubiquitin-like protein ligase binding | 2.13E-07 | Bcl10, Casp8, Cebpb, Myc, Nfkbia, Ppargc1a, Rela, Sqstm1, Tollip, Trib2 |
|  |  | protease binding | 8.43E-07 | Anxa2, Bcl10, Cflar, Il1r1, Lcn2, Tnfaip3, Tnfrsf1a |
|  |  | receptor ligand activity | 1.13E-06 | Ccl2, Csf1, Cxcl1, Cxcl10, Cxcl16, Il1a, Il33, Il6, Lgals3, Tgfb1, Tnfsf9 |
|  |  | ubiquitin protein ligase binding | 1.31E-06 | Bcl10, Casp8, Myc, Nfkbia, Ppargc1a, Rela, Sqstm1, Tollip, Trib2 |
|  |  | chemokine receptor binding | 3.89E-06 | Ccl2, Cxcl1, Cxcl10, Cxcl16, Stat3 |
|  |  | growth factor receptor binding | 1.29E-05 | Il1a, Il1r1, Il6, Jak2, Myd88, Tollip |
|  |  | Toll-like receptor binding | 1.49E-05 | Myd88, Tlr2, Tollip |
|  |  | chemokine activity | 1.74E-05 | Ccl2, Cxcl1, Cxcl10, Cxcl16 |
|  | GO_BP | positive regulation of cytokine production | 1.50E-26 | Atf4, Bcl10, Bcl3, C3ar1, Casp8, Ccl2, Cd14, Cebpb, Csf1r, Fgr, Il1a, Il1r1, Il33, Il4ra, Il6, Jak2, Mapk14, Mapkapk2, Myd88, Nr4a3, Rela, Stat3, Tgfb1, Tlr2, Tnfrsf1a, Tnfsf9, Tyrobp |
| 24h | GO_BP | positive regulation of cytokine production | 3.12E-51 | Tyrobp, Cyba, Xbp1, Serpine1, C3ar1, Clec4e, Slc11a1, Stat3, Ddx21, Bcl3, Lgals9, Nras, Casp8, Clec4n, Ccl2, Cd14, Zc3hav1, Pycard, Atf4, Tgfb1, Hspd1, Plcg2, Jak2, Anxa1, Tnfrsf1a, Trim32, Bcl10, Casp1, Cebpb, Il1rap, Unc93b1, Tbk1, Tlr2, Trim15, Il4ra, Drd2, Traf2, Myd88, Ptpn11, Rela, Hdac1, Adam17, Thbs1, Lrrfip2, Il33, Tlr3, Fgr, Mapk14, Il1r1, Ifnar1, Irf8, C3, Eif2ak2, Cd81, Cd36, Polr3d |
|  |  | positive regulation of defense response | 1.14E-37 | Tlr13, Trim30a, Cyba, Arf6, Serpine1, Cd300lf, Fcgr1, Grn, Clec4e, Cxcl1, Ctss, Lgals9, Cd14, Zc3hav1, Pycard, Hspd1, Jak2, Tnfrsf1a, Bcl10, Tyro3, Cebpb, Tril, Cebpa, Unc93b1, Tbk1, Irgm1, Tlr2, Trim15, Myd88, Rela, Lrrfip2, Il33, Tlr3, Btk, Ikbke, Snca, Traf3, C3, Cd81, Pla2g4a, Cd36, Polr3d, Plscr1 |
|  |  | regulation of immune effector process | 2.41E-30 | Xbp1, Fcgr1, Grn, Lgals3, Ddx21, Cxcl1, Lgals9, Ccl2, Zc3hav1, Pycard, Cr1l, Tgfb1, Hspd1, Anxa1, Stat6, Tril, Ripk3, Ptpn6, Tlr2, Trim15, Itgam, Il4ra, Traf2, Il33, Itgb2, Tlr3, Fgr, Hmox1, Apobec3, Mapk14, Angpt1, Btk, Rac2, Il1r1, Cd55, Traf3, C3, Cd81, Cd36 |
|  |  | myeloid leukocyte activation | 4.05E-30 | Tyrobp, Clec4d, Cd300lf, Grn, Slc11a1, Lgals9, Pycard, Tgfb1, Hspd1, Jak2, Cd48, Bcl10, Casp1, Cebpa, Tlr2, Itgam, Il4ra, Thbs1, Ltbr, Il33, Chga, Itgb2, Tlr3, Fgr, Hmox1, Btk, C1qa, Rac2, Snca, Lcp2, Plscr1 |
|  |  | positive regulation of response to external stimulus | 4.39E-30 | Cyba, Serpine1, Fcgr1, C3ar1, Grn, Cxcl1, Ctss, Lgals9, Ccl2, Csf1, Zc3hav1, Tgfb1, Hspd1, Jak2, Tnfrsf1a, Edn1, Cebpb, Cebpa, Tbk1, Tlr2, Trim15, Myd88, Adam17, Thbs1, Il33, Tlr3, Mapk14, Btk, Rac2, Snca, C3, Cxcl10, Ly86, Cd81, Pla2g4a |
|  |  | response to molecule of bacterial origin | 1.94E-28 | Xbp1, Serpine1, Slc11a1, Cxcl1, Zfp36, Lgals9, Ccl2, Cd14, Pycard, Tgfb1, Plcg2, Jak2, Bcl10, Casp1, Cebpb, Tlr2, Myd88, Gbp2, Rela, Adam17, Axl, Mapk8, Irf5, Abca1, Mapk14, Cxcl2, Snca, Gbp6, Akt1, Ifnar1, Irf8, Eif2ak2, Cxcl10, Ly86, Cd36, Plscr1 |
|  |  | leukocyte migration | 1.53E-27 | Spp1, Kitl, Serpine1, C3ar1, Lgals3, Cxcl1, Lgals9, Cdc42, Ccl2, Csf1, Pycard, Tgfb1, Anxa1, Icam1, Trpm2, Edn1, Ripk3, Tlr2, Itgam, Myd88, Adam17, Thbs1, Il33, Chga, Itgb2, Mapk14, Eps8, Cxcl2, Rac2, Dusp1, Akt1, Il1r1, Cxcl10, Cd81 |
|  |  | response to lipopolysaccharide | 9.45E-27 | Xbp1, Serpine1, Slc11a1, Cxcl1, Zfp36, Lgals9, Ccl2, Cd14, Pycard, Tgfb1, Plcg2, Jak2, Bcl10, Casp1, Cebpb, Myd88, Gbp2, Rela, Adam17, Axl, Mapk8, Abca1, Mapk14, Cxcl2, Snca, Gbp6, Akt1, Ifnar1, Irf8, Eif2ak2, Cxcl10, Ly86, Cd36, Plscr1 |
|  |  | cytokine-mediated signaling pathway | 2.81E-26 | Cd300lf, Stat3, Cxcl1, Ccl2, Csf1, Pycard, Jak2, Tnfrsf1a, Trim32, Stat6, Casp1, Il1rap, Cebpa, Ptpn6, Tbk1, Irgm1, Traf2, Myd88, Rela, Adam17, Ifitm2, Axl, Cyld, Irf5, Angpt1, Ikbke, Cxcl2, Iigp1, Akt1, Il1r1, Ifnar1, Traf3, Cxcl10 |
|  |  | regulation of innate immune response | 4.41E-26 | Tlr13, Trim30a, Cyba, Arf6, Cd300lf, Grn, Clec4e, Lgals9, Cd14, Zc3hav1, Pycard, Hspd1, Bcl10, Tyro3, Tril, Unc93b1, Tbk1, Irgm1, Tlr2, Trim15, Drd2, Myd88, Rela, Lrrfip2, Tlr3, Fgr, Ikbke, Traf3, Cd36, Polr3d, Plscr1 |
|  | GO_CC | membrane raft | 5.87E-21 | S100a10, Ptch1, Fcgr1, Gnai3, Ctsb, Casp8, Cd14, Hspd1, Jak2, Hck, Cd48, Tnfrsf1a, Icam1, Bcl10, Stat6, Anxa2, Tlr2, Itgam, Traf2, Adam17, Itgb2, Hmox1, Abca1, Angpt1, Btk, Lcp2, Cd55, Cd36, Plscr1 |
|  |  | membrane microdomain | 6.33E-21 | S100a10, Ptch1, Fcgr1, Gnai3, Ctsb, Casp8, Cd14, Hspd1, Jak2, Hck, Cd48, Tnfrsf1a, Icam1, Bcl10, Stat6, Anxa2, Tlr2, Itgam, Traf2, Adam17, Itgb2, Hmox1, Abca1, Angpt1, Btk, Lcp2, Cd55, Cd36, Plscr1 |
|  |  | membrane region | 1.55E-20 | S100a10, Ptch1, Fcgr1, Gnai3, Ctsb, Casp8, Cd14, Hspd1, Jak2, Hck, Cd48, Tnfrsf1a, Icam1, Bcl10, Stat6, Anxa2, Tlr2, Itgam, Traf2, Adam17, Itgb2, Hmox1, Abca1, Angpt1, Btk, Lcp2, Cd55, Cd36, Plscr1 |
|  |  | receptor complex | 2.17E-09 | Cd14, Cr1l, Hspd1, Tnfrsf1a, Tyro3, Tril, Ptpn6, Tlr2, Il4ra, Traf2, Vldlr, Axl, Itgb2, Mertk, Eps8, Traf3, Notch1, Cd36 |
|  |  | extracellular organelle | 2.63E-07 | Serpine1, Hspd1, Anxa1, Icam1, Anxa2, Gbp2, Gbp6, Iigp1, Cd81 |
|  |  | plasma membrane raft | 5.43E-07 | Ptch1, Ctsb, Jak2, Hck, Itgam, Itgb2, Hmox1, Lcp2, Cd36 |
|  |  | endocytic vesicle | 1.12E-06 | Arf6, Slc11a1, Cdc42, Ccl2, Unc93b1, Irgm1, Drd2, Abca1, Rab8a, Pik3c3 |
|  |  | lysosome | 1.61E-06 | Grn, Slc11a1, Ctss, Ctsb, Zc3hav1, Hck, Anxa1, Trpm2, Gabarap, Bcl10, Unc93b1, Anxa2, Irgm1, Ifnar1, Pik3c3 |
|  |  | actin cytoskeleton | 1.64E-06 | Cyba, Hck, Anxa1, Inpp5d, Trim32, Gabarap, Msrb1, Anxa2, Ptpn11, Gbp2, Adam17, Mapk8, Fgr, Rac2, Snca, Tsc1 |
|  |  | lytic vacuole | 1.66E-06 | Grn, Slc11a1, Ctss, Ctsb, Zc3hav1, Hck, Anxa1, Trpm2, Gabarap, Bcl10, Unc93b1, Anxa2, Irgm1, Ifnar1, Pik3c3 |
|  | GO_MF | cytokine receptor binding | 1.14E-10 | Kitl, Cd300lf, Stat3, Cxcl1, Casp8, Ccl2, Csf1, Pycard, Tgfb1, Jak2, Il1rap, Traf2, Myd88, Adam17, Angpt1, Cxcl2, Traf3, Cxcl10 |
|  |  | ubiquitin-like protein ligase binding | 4.57E-08 | Xbp1, Casp8, Hspd1, Gabarap, Bcl10, Cebpb, Traf2, Rela, Ppargc1a, Ltbr, H2-Ab1, Myc, Ikbke, Traf3, Trib2 |
|  |  | lipoprotein particle binding | 6.96E-08 | Msr1, Hspd1, Thbs1, Vldlr, Abca1, Cd36 |
|  |  | protein-lipid complex binding | 6.96E-08 | Msr1, Hspd1, Thbs1, Vldlr, Abca1, Cd36 |
|  |  | protein phosphatase binding | 8.28E-08 | Eif4ebp1, Stat3, Lgals3, Stat6, Tbk1, Traf2, Ppp1ca, Ppp1cc, Mapk14, Akt1, Traf3 |
|  |  | ubiquitin protein ligase binding | 1.45E-07 | Xbp1, Casp8, Hspd1, Gabarap, Bcl10, Traf2, Rela, Ppargc1a, Ltbr, H2-Ab1, Myc, Ikbke, Traf3, Trib2 |
|  |  | phosphatase binding | 1.01E-06 | Eif4ebp1, Stat3, Lgals3, Stat6, Tbk1, Traf2, Ppp1ca, Ppp1cc, Mapk14, Akt1, Traf3 |
|  |  | cytokine activity | 1.11E-06 | Spp1, Kitl, Grn, Cxcl1, Ccl2, Csf1, Tgfb1, Edn1, Il33, Cxcl2, Cxcl10 |
|  |  | protease binding | 2.35E-06 | Xbp1, Serpine1, Pycard, Hspd1, Tnfrsf1a, Bcl10, Anxa2, Lcn2, Il1r1 |
|  |  | growth factor receptor binding | 2.35E-06 | Cd300lf, Pycard, Jak2, Il1rap, Myd88, Adam17, Angpt1, Il1r1, Plscr1 |
| 72h | GO_BP | positive regulation of cytokine production | 2.20E-66 | Hif1a, C3ar1, Ifngr1, Cd14, Pycard, Havcr2, Hdac1, Slc11a1, Tgfb1, Lgals9, Thbs1, Trim32, Xbp1, Clec4n, Csf1r, C5ar1, Tnfrsf1a, Bcl10, P2rx7, Casp1, Dlk1, Cyba, Naip5, Tlr2, Ly96, Drd2, Mapk3, Rela, Adam17, Tlr7, Cebpb, Anxa1, Cd1d1, Tyrobp, Irf8, Tlr1, Myd88, Casp8, Lum, Nras, Plcg2, Tmem173, Stat3, Lbp, Ccl2, Clec4e, Zc3hav1, Unc93b1, Tlr6, Tlr4, Il33, Syk, Il4ra, Il1rl2, Mavs, Rora, Mapkapk2, Fgr, Polr3c, Mapk14, Jak2, Ifnar1, Tirap, C3, Akirin2, Ifih1, Polr3g, Serpine1, Bcl3, Cd36, Ddx21, Trim15, Traf2, Cd40, Clec5a, Stat1, Rnf135 |
|  |  | positive regulation of defense response | 1.75E-48 | Grn, Fcgr1, Cd14, Pycard, Arf6, Havcr2, Cebpa, Ctss, Trim30a, Tlr13, Lgals9, Cd300lf, Ubqln1, Tnfrsf1a, Bcl10, Cyba, Tlr2, Ly96, Pik3cg, Mapk3, Rela, Tlr7, Cebpb, Tlr1, Myd88, Btk, Tmem173, Cav1, Lbp, Clec4e, Pla2g4a, Zc3hav1, Unc93b1, Tlr6, Tlr4, Il33, Nlrc5, Plscr1, Mavs, Traf3, Sarm1, Mapkapk2, Irak2, Clock, Polr3c, Nr1h3, Ikbke, Jak2, Tirap, Nfkbia, C3, Ifih1, Polr3g, Serpine1, Snca, Cd36, Trim15, Zbp1, Tnfaip3 |
|  |  | response to molecule of bacterial origin | 3.26E-44 | Cd14, Pycard, Havcr2, Axl, Gbp2, Slc11a1, Tgfb1, Lgals9, Cd180, Xbp1, C5ar1, Ly86, Bcl10, P2rx7, Casp1, Tlr2, Ly96, Irf5, Mapk3, Rela, Adam17, Cebpb, Irf8, Tlr1, Myd88, Plcg2, Mapk8, Cxcl10, Lbp, Ccl2, Tlr6, Tlr4, Plscr1, Cxcl16, Zfp36, Nfkb1, Mapkapk2, Irak2, Nr1h3, Mapk14, Abca1, Zc3h12a, Jak2, Ifnar1, Sirpa, Tirap, Nfkbia, Akirin2, Serpine1, Snca, Mrc1, Cd36, Mapk1, Akt1, Stat1, Tnfaip3 |
|  |  | regulation of innate immune response | 1.61E-41 | Grn, Cd14, Pycard, Arf6, Havcr2, Trim30a, Tlr13, Lgals9, Cd300lf, Ubqln1, Bcl10, Adar, Cyba, Tlr2, Ly96, Drd2, Rela, Tlr7, Tlr1, Myd88, Tmem173, Cav1, Lbp, Clec4e, Zc3hav1, Unc93b1, Tlr6, Tlr4, Nlrc5, Plscr1, Serping1, Mavs, Traf3, Sarm1, Mapkapk2, Irak2, Fgr, Polr3c, Nr1h3, Ikbke, Tirap, Nfkbia, Ifih1, Polr3g, Cd36, Trim15, Zbp1, Rnf135, Tnfaip3 |
|  |  | myeloid leukocyte activation | 1.13E-39 | Grn, Ifngr1, Pycard, Havcr2, Cebpa, Lcp2, Ltbr, Slc11a1, Tgfb1, Lgals9, Thbs1, Itgam, C5ar1, Cd300lf, Bcl10, Casp1, Tlr2, Chga, Itgb2, Tlr7, C1qa, Cd1d1, Clec4d, Tyrobp, Tlr1, Rac2, Btk, Lbp, Tlr6, Tlr4, Il33, Syk, Plscr1, Il4ra, Rora, Fgr, Hmox1, Nr1h3, Jak2, Cd48, Relb, Snca, Prkcd |
|  |  | regulation of DNA-binding transcription factor activity | 2.12E-39 | Hck, Pycard, Ppargc1b, Havcr2, Tgfb1, Ptch1, Trim32, Ppargc1a, Bcl10, Tlr2, Itgb2, Mapk3, Rela, Trim25, Icam1, Ezh2, Myd88, Trib2, Camk2a, Trim21, Tmem173, Cav1, Irak4, Trim13, Stat3, Vegfa, Tlr6, Tlr4, Ripk3, Syk, Nlrc5, Sirt1, Mavs, Cyld, Traf3, Ikbkb, Irak2, Clock, Cflar, Hmox1, Klf4, Zc3h12a, Jak2, Tirap, Nfkbia, Cd36, Mapk1, Trim15, Traf2, Akt1, Cd40, Tnfaip3, Trim14 |
|  |  | response to lipopolysaccharide | 3.17E-39 | Cd14, Pycard, Havcr2, Axl, Gbp2, Slc11a1, Tgfb1, Lgals9, Cd180, Xbp1, Ly86, Bcl10, P2rx7, Casp1, Ly96, Mapk3, Rela, Adam17, Cebpb, Irf8, Myd88, Plcg2, Mapk8, Cxcl10, Lbp, Ccl2, Tlr4, Plscr1, Cxcl16, Zfp36, Nfkb1, Mapkapk2, Irak2, Nr1h3, Mapk14, Abca1, Zc3h12a, Jak2, Ifnar1, Sirpa, Tirap, Nfkbia, Akirin2, Serpine1, Snca, Mrc1, Cd36, Mapk1, Akt1, Stat1, Tnfaip3 |
|  |  | cellular response to biotic stimulus | 1.60E-38 | Cd14, Pycard, Havcr2, Axl, Gbp2, Tgfb1, Cd180, Xbp1, Ly86, Bcl10, Casp1, Tlr2, Ly96, Mapk3, Rela, Cebpb, Irf8, Tlr1, Myd88, Btk, Mapk8, Txnip, Cxcl10, Lbp, Ccl2, Tlr6, Tlr4, Syk, Plscr1, Cxcl16, Zfp36, Nfkb1, Irak2, Nr1h3, Mapk14, Abca1, Zc3h12a, Jak2, Sirpa, Tirap, Nfkbia, Serpine1, Mrc1, Cd36, Mapk1, Akt1, Stat1, Tnfaip3 |
|  |  | positive regulation of innate immune response | 3.56E-38 | Cd14, Pycard, Arf6, Havcr2, Trim30a, Tlr13, Lgals9, Cd300lf, Ubqln1, Bcl10, Cyba, Tlr2, Ly96, Rela, Tlr7, Tlr1, Myd88, Tmem173, Cav1, Lbp, Clec4e, Zc3hav1, Unc93b1, Tlr6, Tlr4, Nlrc5, Plscr1, Mavs, Traf3, Sarm1, Mapkapk2, Irak2, Polr3c, Nr1h3, Ikbke, Tirap, Nfkbia, Ifih1, Polr3g, Cd36, Trim15, Zbp1, Tnfaip3 |
|  |  | pattern recognition receptor signaling pathway | 6.30E-38 | Cd14, Arf6, Havcr2, Trim30a, Tlr13, Lgals9, Cd300lf, Ubqln1, Bcl10, Cyba, Tlr2, Ly96, Rela, Tlr7, Tlr1, Myd88, Tmem173, Cav1, Lbp, Clec4e, Zc3hav1, Unc93b1, Tlr6, Tlr4, Mavs, Traf3, Sarm1, Mapkapk2, Irak2, Nr1h3, Tirap, Nfkbia, Ifih1, Cd36, Trim15, Tnfaip3 |
|  | GO_CC | membrane raft | 2.59E-23 | Hck, Fcgr1, Cd14, Gnai3, Lcp2, Ctsb, Ptch1, Itgam, Tnfrsf1a, Bcl10, Anxa2, Stat6, Tlr2, Itgb2, Mapk3, Adam17, S100a10, Icam1, Tlr1, Casp8, Btk, Cav1, Orai1, Tlr6, Tlr4, Plscr1, Cd55, Cflar, Hmox1, Abca1, Gnai2, Jak2, Cd48, Birc3, Cd36, Mapk1, Traf2 |
|  |  | membrane microdomain | 2.85E-23 | Hck, Fcgr1, Cd14, Gnai3, Lcp2, Ctsb, Ptch1, Itgam, Tnfrsf1a, Bcl10, Anxa2, Stat6, Tlr2, Itgb2, Mapk3, Adam17, S100a10, Icam1, Tlr1, Casp8, Btk, Cav1, Orai1, Tlr6, Tlr4, Plscr1, Cd55, Cflar, Hmox1, Abca1, Gnai2, Jak2, Cd48, Birc3, Cd36, Mapk1, Traf2 |
|  |  | membrane region | 8.85E-23 | Hck, Fcgr1, Cd14, Gnai3, Lcp2, Ctsb, Ptch1, Itgam, Tnfrsf1a, Bcl10, Anxa2, Stat6, Tlr2, Itgb2, Mapk3, Adam17, S100a10, Icam1, Tlr1, Casp8, Btk, Cav1, Orai1, Tlr6, Tlr4, Plscr1, Cd55, Cflar, Hmox1, Abca1, Gnai2, Jak2, Cd48, Birc3, Cd36, Mapk1, Traf2 |
|  |  | receptor complex | 1.95E-11 | Cd14, Ptpn6, Axl, Csf1r, Cr1l, Tnfrsf1a, Mertk, Tlr2, Ly96, Itgb2, Tlr7, Tlr1, Cd200r1, Tlr6, Tlr4, Syk, Il4ra, Traf3, Ikbkb, Vldlr, Nr1h3, Cd36, Eps8, Traf2, Cd40 |
|  |  | plasma membrane raft | 3.61E-09 | Hck, Lcp2, Ctsb, Ptch1, Itgam, Itgb2, Mapk3, Cav1, Orai1, Hmox1, Jak2, Cd36, Mapk1 |
|  |  | lysosome | 1.81E-08 | Grn, Lgmn, Hck, Gabarap, Mtor, Ctss, Ctsb, Slc11a1, H2-Aa, Bcl10, Anxa2, Tlr7, Anxa1, Trpm2, Cxcr4, Cd1d1, Tecpr1, Zc3hav1, Unc93b1, Ifnar1, Pik3c3, Tnfaip3 |
|  |  | lytic vacuole | 1.89E-08 | Grn, Lgmn, Hck, Gabarap, Mtor, Ctss, Ctsb, Slc11a1, H2-Aa, Bcl10, Anxa2, Tlr7, Anxa1, Trpm2, Cxcr4, Cd1d1, Tecpr1, Zc3hav1, Unc93b1, Ifnar1, Pik3c3, Tnfaip3 |
|  |  | endocytic vesicle | 2.88E-08 | Arf6, Slc11a1, Cdc42, Drd2, Tlr7, Tlr1, Was, Ccl2, Unc93b1, Syk, Rab8a, Abca1, Tirap, Pik3c3 |
|  |  | phagocytic vesicle | 1.32E-07 | Slc11a1, Cdc42, Tlr7, Tlr1, Was, Unc93b1, Syk, Rab8a, Abca1, Pik3c3 |
|  |  | midbody | 1.40E-07 | Arf6, Gnai3, Ptch1, Anxa2, Pin1, Rab8a, Cyld, Gnai2, Bcl3, Hsp90b1, Pik3c3, Ctnnd1 |
|  | GO_MF | ubiquitin-like protein ligase binding | 6.28E-16 | Hif1a, Gabarap, Ltbr, H2-Ab1, Xbp1, Ppargc1a, Bcl10, Rela, Cebpb, Cxcr4, Rb1, Casp8, Trib2, Tmem173, Txnip, Tank, Syk, Stub1, Traf3, Myc, Ikbke, Mfn2, Nfkbia, Trib3, Traf2, Cd40, Stat1, Daxx |
|  |  | phosphatase binding | 2.13E-15 | Lgals3, Csf1r, Stat6, Eif4ebp1, Mapk3, Ppp3r1, Ppp1ca, Nfatc2, Stat3, Syk, Ap3b1, Traf3, Ikbkb, Pstpip1, Mapk14, Jak3, Sirpa, Ppp1cc, Mapk1, Hsp90b1, Traf2, Akt1, Stat1 |
|  |  | ubiquitin protein ligase binding | 1.00E-14 | Hif1a, Gabarap, Ltbr, H2-Ab1, Xbp1, Ppargc1a, Bcl10, Rela, Cxcr4, Rb1, Casp8, Trib2, Tmem173, Txnip, Tank, Syk, Stub1, Traf3, Myc, Ikbke, Mfn2, Nfkbia, Trib3, Traf2, Cd40, Daxx |
|  |  | Toll-like receptor binding | 1.03E-14 | Tlr2, Ly96, Tlr1, Myd88, Unc93b1, Tlr6, Syk, Tirap, Cd36 |
|  |  | protein phosphatase binding | 2.39E-12 | Lgals3, Csf1r, Stat6, Eif4ebp1, Ppp1ca, Stat3, Ap3b1, Traf3, Ikbkb, Pstpip1, Mapk14, Jak3, Sirpa, Ppp1cc, Hsp90b1, Traf2, Akt1, Stat1 |
|  |  | pattern recognition receptor activity | 1.08E-09 | Tlr2, Ly96, Tlr7, Trim12c, Clec4e, Tlr4, Cd36 |
|  |  | cytokine receptor binding | 4.45E-09 | Pycard, Tgfb1, Csf1, Cd300lf, Adam17, Myd88, Casp8, Irak4, Cxcl10, Stat3, Vegfa, Ccl2, Kitl, Syk, Cxcl16, Traf3, Cflar, Jak2, Traf2, Stat1 |
|  |  | phosphoprotein binding | 4.54E-09 | Hck, Mtor, Ptpn6, Mapk3, Rb1, Pin1, Plcg2, Syk, Fgr, Sirpa, Snca, Mapk1 |
|  |  | DNA-binding transcription activator activity, RNA polymerase II-specific | 5.79E-09 | Hif1a, Cebpa, Ybx1, Stat6, Rela, Cebpb, Elf1, Nfatc2, Cebpd, Stat3, Nfe2l2, Arntl, Sirt1, Plscr1, Nfkb1, Myc, Clock, Nr1h3, Klf4, Akna, Relb, Bcl11b, Stat1 |
|  |  | protein phosphorylated amino acid binding | 2.11E-08 | Hck, Ptpn6, Mapk3, Pin1, Plcg2, Syk, Fgr, Sirpa, Mapk1 |
| 7d | GO_BP | positive regulation of cytokine production | 2.37E-52 | Hdac1, Tlr2, Tyrobp, Unc93b1, Ly96, Ccl2, Cd14, Csf1r, Tlr1, Trem2, Naip5, Casp1, Trim32, Cebpb, Stat1, Cyba, Dlk1, Irf8, Il1r1, Ddx1, C3ar1, Bcl3, Pycard, Tlr8, Lgals9, Bcl10, Il1rl2, Hif1a, Casp8, Mapk14, Cd36, Tlr7, Zc3hav1, Ddx21, Clec5a, Ifih1, Plcg2, Drd2, Syk, Tlr4, Ifngr1, Myd88, C3, C5ar1, Akirin2, Slc11a1, Tlr6, Fgr, Clec4n, Il33, Tnfrsf1a, Il4ra, Havcr2, Adam17, Xbp1, Serpine1, Lrrfip2, Tmem173, P2rx7, Polr3a |
|  |  | positive regulation of defense response | 1.14E-39 | Ctss, Tlr2, Unc93b1, Ly96, Cd14, Tlr1, Trem2, Irgm1, Grn, Cd86, Cebpb, Cyba, Fcgr1, Arf6, Tlr13, Cebpa, Trim30a, Cd300a, Pycard, Tlr8, Lgals9, Cd300lf, Pik3ap1, Bcl10, Clock, Btk, Cd36, Tlr7, Zc3hav1, Ifih1, Ubqln1, Tlr4, Myd88, C3, Zbp1, Traf3, Tlr6, Il33, Tnfrsf1a, Havcr2, Nfkbia, Dab2ip, Serpine1, Nlrc5, Lrrfip2, Tmem173, Pik3cg |
|  |  | response to molecule of bacterial origin | 1.76E-35 | Abca1, Tlr2, Ly96, Ccl2, Cd14, Tlr1, Trem2, Sirpa, Casp1, Irf5, Cd86, Cebpb, Ly86, Mrc1, Stat1, Irf8, Axl, Mapk8, Pycard, Lgals9, Gbp2, Bcl10, Mapk14, Cd36, Gbp6, Plcg2, Tlr4, Myd88, Cd180, C5ar1, Prdm1, Akirin2, Cxcl16, Slc11a1, Tlr6, Havcr2, Adam17, Xbp1, Nfkbia, Dab2ip, Scarb1, Serpine1, P2rx7, Cxcl10 |
|  |  | myeloid leukocyte activation | 8.43E-35 | Tlr2, Tyrobp, C1qa, Tlr1, Trem2, Casp1, Grn, Nampt, Cd48, Prkcd, Cebpa, Lcp2, Itgb2, Cd300a, Pycard, Tlr8, Lgals9, Cd300lf, Bcl10, Clec4d, Btk, Itgam, Chga, Tlr7, Rac2, Syk, Tlr4, Ifngr1, Hmox1, C5ar1, Slc11a1, Tlr6, Fgr, Il33, Il4ra, Havcr2 |
|  |  | regulation of innate immune response | 6.46E-34 | Tlr2, Unc93b1, Ly96, Cd14, Tlr1, Trem2, Irgm1, Grn, Cd86, Cyba, Arf6, Tlr13, Trim30a, Cd300a, Pycard, Tlr8, Lgals9, Cd300lf, Pik3ap1, Bcl10, Cd36, Tlr7, Zc3hav1, Ifih1, Adar, Ubqln1, Drd2, Tlr4, Myd88, Zbp1, Traf3, Tlr6, Fgr, Havcr2, Nfkbia, Dab2ip, Nlrc5, Lrrfip2, Tmem173 |
|  |  | pattern recognition receptor signaling pathway | 8.24E-33 | Tlr2, Unc93b1, Ly96, Cd14, Tlr1, Cd86, Cyba, Arf6, Tlr13, Trim30a, Cd300a, Tlr8, Lgals9, Cd300lf, Pik3ap1, Bcl10, Cd36, Tlr7, Zc3hav1, Ifih1, Ubqln1, Tlr4, Myd88, Traf3, Tlr6, Havcr2, Nfkbia, Dab2ip, Lrrfip2, Tmem173 |
|  |  | innate immune response-activating signal transduction | 1.32E-32 | Tlr2, Unc93b1, Ly96, Cd14, Tlr1, Cd86, Cyba, Arf6, Tlr13, Trim30a, Cd300a, Tlr8, Lgals9, Cd300lf, Pik3ap1, Bcl10, Cd36, Tlr7, Zc3hav1, Ifih1, Ubqln1, Tlr4, Myd88, Traf3, Tlr6, Havcr2, Nfkbia, Dab2ip, Lrrfip2, Tmem173 |
|  |  | toll-like receptor signaling pathway | 6.21E-32 | Tlr2, Unc93b1, Ly96, Cd14, Tlr1, Cd86, Cyba, Arf6, Tlr13, Trim30a, Cd300a, Tlr8, Lgals9, Cd300lf, Pik3ap1, Bcl10, Cd36, Tlr7, Ubqln1, Tlr4, Myd88, Traf3, Tlr6, Havcr2, Nfkbia, Dab2ip, Lrrfip2 |
|  |  | activation of innate immune response | 9.41E-32 | Tlr2, Unc93b1, Ly96, Cd14, Tlr1, Cd86, Cyba, Arf6, Tlr13, Trim30a, Cd300a, Pycard, Tlr8, Lgals9, Cd300lf, Pik3ap1, Bcl10, Cd36, Tlr7, Zc3hav1, Ifih1, Ubqln1, Tlr4, Myd88, Traf3, Tlr6, Havcr2, Nfkbia, Dab2ip, Lrrfip2, Tmem173 |
|  |  | positive regulation of response to external stimulus | 2.43E-31 | Ctss, Tlr2, Ly96, Ccl2, Trem2, Rarres2, Grn, Cebpb, Ly86, Cyba, Fcgr1, Lgmn, Cebpa, C3ar1, Lgals9, Clock, Csf1, Btk, Mapk14, Vegfa, Zc3hav1, Rac2, Tlr4, Myd88, C3, Cxcr4, Cd180, C5ar1, Tlr6, Il33, Tnfrsf1a, Havcr2, Adam17, Nfkbia, Gas6, Serpine1, Pik3cg, Cxcl10 |
|  | GO_CC | membrane raft | 6.96E-18 | Abca1, Tlr2, Ctsb, Ptch1, Cd14, Hck, Tlr1, Cd48, Fcgr1, S100a10, Lcp2, Itgb2, Bcl10, Btk, Casp8, Itgam, Cd36, Stat6, Ctnnb1, Cflar, Gnai3, Tlr4, Hmox1, Cd55, Tlr6, Tnfrsf1a, Adam17, Scarb1 |
|  |  | membrane microdomain | 7.48E-18 | Abca1, Tlr2, Ctsb, Ptch1, Cd14, Hck, Tlr1, Cd48, Fcgr1, S100a10, Lcp2, Itgb2, Bcl10, Btk, Casp8, Itgam, Cd36, Stat6, Ctnnb1, Cflar, Gnai3, Tlr4, Hmox1, Cd55, Tlr6, Tnfrsf1a, Adam17, Scarb1 |
|  |  | membrane region | 1.74E-17 | Abca1, Tlr2, Ctsb, Ptch1, Cd14, Hck, Tlr1, Cd48, Fcgr1, S100a10, Lcp2, Itgb2, Bcl10, Btk, Casp8, Itgam, Cd36, Stat6, Ctnnb1, Cflar, Gnai3, Tlr4, Hmox1, Cd55, Tlr6, Tnfrsf1a, Adam17, Scarb1 |
|  |  | receptor complex | 1.22E-10 | Ptpn6, Itgax, Tlr2, Ly96, Mertk, Cd14, Csf1r, Tlr1, Axl, Itgb2, Cd200r1, Cd36, Tlr7, Vldlr, Syk, Tlr4, Traf3, Tlr6, Tnfrsf1a, Il4ra, Cr1l |
|  |  | endocytic vesicle | 8.30E-09 | Abca1, Unc93b1, Ccl2, Tlr1, Irgm1, Arf6, Was, Tlr7, Drd2, Syk, Cdc42, Slc11a1, Dab2ip |
|  |  | postsynaptic cytosol | 1.13E-07 | Mtor, Eif4e, Prkcd, Pin1, Eif4ebp1, Pten |
|  |  | phagocytic vesicle | 1.36E-07 | Abca1, Unc93b1, Tlr1, Irgm1, Was, Tlr7, Syk, Cdc42, Slc11a1 |
|  |  | plasma membrane raft | 2.18E-07 | Ctsb, Ptch1, Hck, Lcp2, Itgb2, Itgam, Cd36, Ctnnb1, Hmox1, Scarb1 |
|  |  | host cell part | 1.47E-06 | Axl, Iigp1, Gbp2, Gbp6, Gbp7 |
|  |  | region of cytosol | 1.69E-06 | Mtor, Eif4e, Prkcd, Pin1, Eif4ebp1, Pten |
|  | GO_MF | Toll-like receptor binding | 1.29E-13 | Tlr2, Unc93b1, Ly96, Tlr1, Cd36, Syk, Myd88, Tlr6 |
|  |  | signaling pattern recognition receptor activity | 1.02E-12 | Tlr2, Ly96, Tlr8, Cd36, Tlr7, Tlr4, Trim12c, Scarb1 |
|  |  | pattern recognition receptor activity | 1.82E-12 | Tlr2, Ly96, Tlr8, Cd36, Tlr7, Tlr4, Trim12c, Scarb1 |
|  |  | phosphatase binding | 1.45E-11 | Lgals3, Csf1r, Sirpa, Stat1, Cd300a, Eif4ebp1, Pstpip1, Mapk14, Stat6, Ctnnb1, Syk, Ppp1ca, Ppp3r1, Traf3, Jak3, Nfatc2, Dab2ip |
|  |  | ubiquitin-like protein ligase binding | 1.43E-09 | H2-Ab1, Cebpb, Stat1, Txnip, Gabarap, Ppargc1a, Bcl10, Hif1a, Casp8, Syk, Cxcr4, Myc, Trib2, Traf3, Xbp1, Nfkbia, Rb1, Tmem173 |
|  |  | protein phosphatase binding | 4.90E-09 | Lgals3, Csf1r, Sirpa, Stat1, Eif4ebp1, Pstpip1, Mapk14, Stat6, Ctnnb1, Ppp1ca, Traf3, Jak3, Dab2ip |
|  |  | lipopolysaccharide binding | 5.08E-09 | Tlr2, Ly96, Cd14, Trem2, Tlr4, Scarb1, P2rx7 |
|  |  | cargo receptor activity | 5.36E-09 | Abca1, Mrc1, Itgb2, Msr1, Cd36, Vldlr, Cxcl16, Cd5l, Scarb1, Siglech |
|  |  | ubiquitin protein ligase binding | 2.71E-08 | H2-Ab1, Txnip, Gabarap, Ppargc1a, Bcl10, Hif1a, Casp8, Syk, Cxcr4, Myc, Trib2, Traf3, Xbp1, Nfkbia, Rb1, Tmem173 |
|  |  | cytokine receptor binding | 6.80E-08 | Ccl2, Stat1, Pycard, Cd300lf, Csf1, Casp8, Vegfa, Cflar, Syk, Myd88, Irak4, Cxcl16, Traf3, Adam17, Dab2ip, Cxcl10 |
| 28d | GO_BP | positive regulation of cytokine production | 2.26E-60 | Unc93b1,Csf1r,C3,Tlr2,Lgals9,Slc11a1,Tyrobp,Il33,Mapkapk2,Cyba,Casp1,Cd14,Plcg2,Irf8,Clec5a,Tnfrsf1a,Zc3hav1,Cebpb,Pycard,Naip5,Bcl10,Tlr1,Tgfb1,Lbp,Havcr2,Trem2,Hif1a,Casp8,Serpine1,Drd2,Ly96,Ifngr1,Fgr,Stat3,Dlk1,Peli1,Rela,Hdac1,Cd36,Adam17,P2rx7,Ifih1,C5ar1,Tlr7,Trim32,Eif2ak2,Irf1,Zfpm1,C3ar1,Bcl3,Akirin2,Jak2,Myd88,Tlr4,Il1a,Ddx1,Ccl2,Anxa1,Il1r1,Lrrfip2,Stat1,Clec4n,Tlr3,Xbp1,Tmem173,Syk |
|  |  | positive regulation of defense response | 9.81E-45 | Ctss, Unc93b1, C3, Tlr2, Lgals9, Grn, Tlr13, Il33, Mapkapk2, Cyba, Fcgr1, Cd14, Cd300a, Tnfrsf1a, Zc3hav1, Cebpb, Pycard, Bcl10, Nfkbia, Tlr1, Lbp, Havcr2, Cebpa, Cd86, Trem2, Serpine1, Ly96, Traf3, Peli1, Rela, Tnfaip3, Cd36, Ifih1, Pik3cg, Coch, Tlr7, Cd300lf, Trim30a, Dab2ip, Irf1, Jak2, Myd88, Tlr4, Pik3ap1, Lrrfip2, Ubqln1, Sarm1, Tlr3, Arf6, Btk, Tmem173 |
|  |  | response to molecule of bacterial origin | 2.60E-44 | Cxcl16, Ly86, Tlr2, Lgals9, Slc11a1, Abca1, Mapkapk2, Casp1, Cd14, Plcg2, Irf8, Cebpb, Pycard, Bcl10, Nfkbia, Tlr1, Tgfb1, Axl, Lbp, Havcr2, Cd86, Trem2, Sirpa, Serpine1, Ly96, Peli1, Rela, Tnfaip3, Cd36, Adam17, P2rx7, C5ar1, Mrc1, Dab2ip, Eif2ak2, Zfp36, Gbp2, Mapk1, Cd180, Akirin2, Nfkb1, Jak2, Gbp6, Myd88, Tlr4, Ccl2, Stat1, Irf5, Mapk8, Cxcl10, Xbp1 |
|  |  | pattern recognition receptor signaling pathway | 2.78E-42 | Unc93b1, Tlr2, Lgals9, Tlr13, Mapkapk2, Cyba, Cd14, Cd300a, Zc3hav1, Bcl10, Nfkbia, Tlr1, Lbp, Havcr2, Cd86, Ly96, Traf3, Peli1, Rela, Tnfaip3, Cd36, Ifih1, Tlr7, Cd300lf, Trim30a, Dab2ip, Irf1, Myd88, Tlr4, Pik3ap1, Lrrfip2, Ubqln1, Sarm1, Tlr3, Arf6, Tmem173 |
|  |  | innate immune response-activating signal transduction | 5.00E-42 | Unc93b1, Tlr2, Lgals9, Tlr13, Mapkapk2, Cyba, Cd14, Cd300a, Zc3hav1, Bcl10, Nfkbia, Tlr1, Lbp, Havcr2, Cd86, Ly96, Traf3, Peli1, Rela, Tnfaip3, Cd36, Ifih1, Tlr7, Cd300lf, Trim30a, Dab2ip, Irf1, Myd88, Tlr4, Pik3ap1, Lrrfip2, Ubqln1, Sarm1, Tlr3, Arf6, Tmem173 |
|  |  | activation of innate immune response | 9.91E-41 | Unc93b1, Tlr2, Lgals9, Tlr13, Mapkapk2, Cyba, Cd14, Cd300a, Zc3hav1, Pycard, Bcl10, Nfkbia, Tlr1, Lbp, Havcr2, Cd86, Ly96, Traf3, Peli1, Rela, Tnfaip3, Cd36, Ifih1, Tlr7, Cd300lf, Trim30a, Dab2ip, Irf1, Myd88, Tlr4, Pik3ap1, Lrrfip2, Ubqln1, Sarm1, Tlr3, Arf6, Tmem173 |
|  |  | regulation of innate immune response | 1.86E-40 | Unc93b1, Tlr2, Lgals9, Grn, Tlr13, Mapkapk2, Cyba, Serping1, Cd14, Cd300a, Zc3hav1, Pycard, Bcl10, Nfkbia, Tlr1, Lbp, Havcr2, Cd86, Trem2, Drd2, Ly96, Fgr, Traf3, Peli1, Rela, Tnfaip3, Cd36, Ifih1, Coch, Tlr7, Adar, Cd300lf, Trim30a, Dab2ip, Irf1, Myd88, Tlr4, Pik3ap1, Lrrfip2, Ubqln1, Sarm1, Tlr3, Arf6, Tmem173 |
|  |  | toll-like receptor signaling pathway | 2.73E-40 | Unc93b1, Tlr2, Lgals9, Tlr13, Mapkapk2, Cyba, Cd14, Cd300a, Bcl10, Nfkbia, Tlr1, Lbp, Havcr2, Cd86, Ly96, Traf3, Peli1, Tnfaip3, Cd36, Tlr7, Cd300lf, Trim30a, Dab2ip, Irf1, Myd88, Tlr4, Pik3ap1, Lrrfip2, Ubqln1, Sarm1, Tlr3, Arf6 |
|  |  | response to lipopolysaccharide | 4.32E-40 | Cxcl16, Ly86, Lgals9, Slc11a1, Abca1, Mapkapk2, Casp1, Cd14, Plcg2, Irf8, Cebpb, Pycard, Bcl10, Nfkbia, Tgfb1, Axl, Lbp, Havcr2, Cd86, Trem2, Sirpa, Serpine1, Ly96, Peli1, Rela, Tnfaip3, Cd36, Adam17, P2rx7, Mrc1, Dab2ip, Eif2ak2, Zfp36, Gbp2, Mapk1, Cd180, Akirin2, Nfkb1, Jak2, Gbp6, Myd88, Tlr4, Ccl2, Stat1, Mapk8, Cxcl10, Xbp1 |
|  |  | cellular response to biotic stimulus | 1.47E-37 | Cxcl16, Ly86, Tlr2, Abca1, Casp1, Cd14, Irf8, Cebpb, Pycard, Bcl10, Nfkbia, Tlr1, Tgfb1, Axl, Lbp, Havcr2, Cd86, Trem2, Sirpa, Txnip, Serpine1, Ly96, Rela, Tnfaip3, Cd36, Mrc1, Dab2ip, Zfp36, Gbp2, Mapk1, Cd180, Nfkb1, Jak2, Gbp6, Myd88, Tlr4, Ccl2, Stat1, Mapk8, Btk, Cxcl10, Xbp1, Syk |
|  | GO_CC | membrane raft | 8.53E-19 | Cd48, Tlr2, Ctsb, Angpt1, Abca1, Anxa2, Hck, Fcgr1, Cd14, Tnfrsf1a, S100a10, Icam1, Itgb2, Lcp2, Itgam, Bcl10, Tlr1, Gnai3, Stat6, Casp8, Cd36, Adam17, Ptch1, Mapk1, Jak2, Tlr4, Cflar, Btk, Gnai2 |
|  |  | membrane microdomain | 9.20E-19 | Cd48, Tlr2, Ctsb, Angpt1, Abca1, Anxa2, Hck, Fcgr1, Cd14, Tnfrsf1a, S100a10, Icam1, Itgb2, Lcp2, Itgam, Bcl10, Tlr1, Gnai3, Stat6, Casp8, Cd36, Adam17, Ptch1, Mapk1, Jak2, Tlr4, Cflar, Btk, Gnai2 |
|  |  | membrane region | 2.22E-18 | Cd48, Tlr2, Ctsb, Angpt1, Abca1, Anxa2, Hck, Fcgr1, Cd14, Tnfrsf1a, S100a10, Icam1, Itgb2, Lcp2, Itgam, Bcl10, Tlr1, Gnai3, Stat6, Casp8, Cd36, Adam17, Ptch1, Mapk1, Jak2, Tlr4, Cflar, Btk, Gnai2 |
|  |  | receptor complex | 3.43E-08 | Csf1r, Tlr2, Itgax, Cd14, Tnfrsf1a, Itgb2, Ptpn6, Tlr1, Axl, Cr1l, Mertk, Ly96, Traf3, Cd36, Cd200r1, Tlr7, Tlr4, Syk |
|  |  | endocytic vesicle | 8.46E-08 | Unc93b1, Slc11a1, Abca1, Tlr1, Cdc42, Drd2, Tlr7, Dab2ip, Was, Ccl2, Arf6, Syk |
|  |  | lysosome | 1.32E-07 | Ctss, Unc93b1, Grn, Ctsb, Slc11a1, Anxa2, Hck, Zc3hav1, Bcl10, Cxcr4, Mtor, H2-Aa, Gabarap, Tnfaip3, Trpm2, Tlr7, Anxa1, Lgmn |
|  |  | lytic vacuole | 1.36E-07 | Ctss, Unc93b1, Grn, Ctsb, Slc11a1, Anxa2, Hck, Zc3hav1, Bcl10, Cxcr4, Mtor, H2-Aa, Gabarap, Tnfaip3, Trpm2, Tlr7, Anxa1, Lgmn |
|  |  | collagen-containing extracellular matrix | 2.83E-07 | Lgals3, Lgals9, Dcn, Ctsb, Anxa2, Serping1, S100a10, Mfge8, Rarres2, Tgfb1, Serpine1, Coch, Igf1, Mmp9, Anxa1, Vegfa |
|  |  | extracellular matrix | 5.48E-07 | Lgals3, Lgals9, Dcn, Ctsb, Anxa2, Serping1, S100a10, Mfge8, Rarres2, Tgfb1, Serpine1, Ptx3, Coch, Igf1, Cd180, Mmp9, Anxa1, Vegfa |
|  |  | extracellular organelle | 1.14E-06 | Anxa2, Icam1, Cd86, Anpep, Serpine1, Gbp2, Gbp7, Gbp6, Anxa1 |
|  | GO_MF | cytokine receptor binding | 5.58E-12 | Cxcl16, Angpt1, Pycard, Tgfb1, Casp8, Stat3, Traf3, Adam17, Csf1, Cd300lf, Dab2ip, Jak2, Myd88, Il1a, Irak4, Ccl2, Stat1, Vegfa, Cflar, Cxcl10, Syk |
|  |  | Toll-like receptor binding | 1.94E-11 | Unc93b1, Tlr2, Tlr1, Ly96, Cd36, Myd88, Syk |
|  |  | ubiquitin-like protein ligase binding | 3.94E-11 | Cebpb, Bcl10, H2-Ab1, Nfkbia, Ltbr, Cxcr4, Rb1, Gabarap, Hif1a, Txnip, Casp8, Traf3, Rela, Trib2, Stat1, Ppargc1a, Myc, Xbp1, Tmem173, Syk |
|  |  | phosphatase binding | 2.07E-10 | Lgals3, Csf1r, Cd300a, Eif4ebp1, Sirpa, Stat6, Stat3, Traf3, Dab2ip, Mapk1, Nfatc2, Ppp1ca, Ppp3r1, Stat1, Hsp90b1, Syk |
|  |  | ubiquitin protein ligase binding | 8.02E-10 | Bcl10, H2-Ab1, Nfkbia, Ltbr, Cxcr4, Rb1, Gabarap, Hif1a, Txnip, Casp8, Traf3, Rela, Trib2, Ppargc1a, Myc, Xbp1, Tmem173, Syk |
|  |  | DNA-binding transcription activator activity, RNA polymerase II-specific | 1.28E-09 | Nfe2l2, Cebpb, Cebpa, Stat6, Hif1a, Stat3, Cebpd, Rela, Ybx1, Irf1, Nfatc2, Nfkb1, Srebf1, Relb, Stat1, Nr3c1, Akna, Sirt1, Myc, Bcl11b, Arntl |
|  |  | lipopolysaccharide binding | 5.99E-09 | Tlr2, Cd14, Lbp, Trem2, Ly96, P2rx7, Tlr4 |
|  |  | signaling pattern recognition receptor activity | 7.41E-09 | Tlr2, Ly96, Cd36, Tlr7, Trim12c, Tlr4 |
|  |  | pattern recognition receptor activity | 1.10E-08 | Tlr2, Ly96, Cd36, Tlr7, Trim12c, Tlr4 |
|  |  | complement binding | 4.29E-08 | Serping1, Itgb2, Ptx3, C5ar1, C3ar1, Cfh |

GO: Gene Ontology; h: hours; d: days. BP: Biological process; CC: Cellular component; MF: Molecular function.

**Supplemental Table 3 Ten KEGG terms of upregulated immune-related genes and downregulated immune-related genes with the lowest P values at each time point**

| Sub-groups | | KEGG terms | pvalue | p.adjust | Genes |
| --- | --- | --- | --- | --- | --- |
| 0.5h | Upregulate innate  immune-related genes | Chagas disease | 2.91E-05 | 0.00099024 | C1qa, Serpine1, Ccl2 |
|  |  | Complement and coagulation cascades | 0.001568385 | 0.020932345 | C1qa, Serpine1 |
|  |  | AGE-RAGE signaling pathway in diabetic complications | 0.001846972 | 0.020932345 | Serpine1, Ccl2 |
|  |  | Fluid shear stress and atherosclerosis | 0.003922668 | 0.033342675 | Dusp1, Ccl2 |
|  |  | Coronavirus disease - COVID-19 | 0.010633237 | 0.072306014 | C1qa, Ccl2 |
|  |  | Malaria | 0.037727252 | 0.170889404 | Ccl2 |
|  |  | p53 signaling pathway | 0.047455833 | 0.170889404 | Serpine1 |
|  |  | Pertussis | 0.050680421 | 0.170889404 | C1qa |
|  |  | Rheumatoid arthritis | 0.057102313 | 0.170889404 | Ccl2 |
|  |  | IL-17 signaling pathway | 0.059660912 | 0.170889404 | Ccl2 |
|  | Downregulate innate  immune-related genes | GnRH secretion | 0.007059615 | 0.02614672 | Spp1 |
|  |  | ECM-receptor interaction | 0.009861049 | 0.02614672 | Spp1 |
|  |  | Toll-like receptor signaling pathway | 0.011205737 | 0.02614672 | Spp1 |
|  |  | Apelin signaling pathway | 0.015239803 | 0.026669655 | Spp1 |
|  |  | Focal adhesion | 0.022523532 | 0.031532945 | Spp1 |
|  |  | PI3K-Akt signaling pathway | 0.040228597 | 0.040564769 | Spp1 |
|  |  | Human papillomavirus infection | 0.040564769 | 0.040564769 | Spp1 |
| 4h | Upregulate innate  immune-related genes | TNF signaling pathway | 7.28E-21 | 1.22E-18 | Ccl2, Cebpb, Tnfrsf1a, Tnfaip3, Atf4, Nfkbia, Bcl3, Icam1, Cxcl1, Birc3, Cxcl10, Nfkb1, Csf1, Il6, Mapk14, Rela, Cflar, Casp8 |
|  |  | Toxoplasmosis | 6.72E-15 | 5.64E-13 | Tnfrsf1a, Nfkbia, Birc3, Nfkb1, Tgfb1, Mapk14, Rela, Tlr2, Irgm1, Igtp, Myd88, Jak2, Stat3, Casp8 |
|  |  | NF-kappa B signaling pathway | 1.00E-13 | 5.60E-12 | Tnfrsf1a, Tnfaip3, Il1r1, Nfkbia, Cd14, Icam1, Cxcl1, Birc3, Nfkb1, Bcl10, Rela, Cflar, Myd88 |
|  |  | Lipid and atherosclerosis | 3.48E-13 | 1.46E-11 | Ccl2, Tnfrsf1a, Atf4, Nfkbia, Cd14, Icam1, Cxcl1, Nfkb1, Il6, Mapk14, Rela, Tlr2, Myd88, Jak2, Stat3, Casp8 |
|  |  | IL-17 signaling pathway | 4.54E-13 | 1.53E-11 | Ccl2, Lcn2, Cebpb, Tnfaip3, Nfkbia, Cxcl1, Cxcl10, Nfkb1, Il6, Mapk14, Rela, Casp8 |
|  |  | Toll-like receptor signaling pathway | 1.45E-12 | 4.05E-11 | Nfkbia, Cd14, Cxcl10, Nfkb1, Il6, Mapk14, Rela, Tlr2, Myd88, Cd86, Casp8, Tollip |
|  |  | Chagas disease | 2.07E-12 | 4.98E-11 | Ccl2, Tnfrsf1a, Nfkbia, Nfkb1, Il6, Tgfb1, Mapk14, Rela, Tlr2, Cflar, Myd88, Casp8 |
|  |  | Tuberculosis | 6.70E-12 | 1.41E-10 | Cebpb, Tnfrsf1a, Cd14, Nfkb1, Il6, Il1a, Tgfb1, Mapk14, Bcl10, Rela, Tlr2, Myd88, Jak2, Casp8 |
|  |  | Kaposi sarcoma-associated herpesvirus infection | 9.29E-12 | 1.73E-10 | Tnfrsf1a, Zfp36, Myc, Nfkbia, Icam1, Cxcl1, Nfkb1, Il6, Mapk14, Rela, Mapkapk2, Cd86, Jak2, Stat3, Casp8 |
|  |  | Hepatitis B | 3.05E-11 | 5.12E-10 | Myc, Atf4, Nfkbia, Nfkb1, Il6, Tgfb1, Mapk14, Rela, Tlr2, Myd88, Jak2, Stat3, Casp8 |
|  | Downregulate innate  immune-related genes | Cholesterol metabolism | 0.032504912 | 0.155505487 | Pltp |
|  |  | Hedgehog signaling pathway | 0.038378389 | 0.155505487 | Ptch1 |
|  |  | Basal cell carcinoma | 0.041628572 | 0.155505487 | Ptch1 |
|  |  | Acute myeloid leukemia | 0.046163445 | 0.155505487 | Csf1r |
|  |  | Adipocytokine signaling pathway | 0.046809821 | 0.155505487 | Ppargc1a |
|  |  | PPAR signaling pathway | 0.058382336 | 0.155505487 | Pltp |
|  |  | Longevity regulating pathway | 0.059021805 | 0.155505487 | Ppargc1a |
|  |  | Hematopoietic cell lineage | 0.061576062 | 0.155505487 | Csf1r |
|  |  | Viral protein interaction with cytokine and cytokine receptor | 0.062213723 | 0.155505487 | Csf1r |
|  |  | TGF-beta signaling pathway | 0.062213723 | 0.155505487 | Rgmb |
| 0.5h | Upregulate innate  immune-related genes | Chagas disease | 2.91E-05 | 0.00099024 | C1qa, Serpine1, Ccl2 |
|  |  | Complement and coagulation cascades | 0.001568385 | 0.020932345 | C1qa, Serpine1 |
|  |  | AGE-RAGE signaling pathway in diabetic complications | 0.001846972 | 0.020932345 | Serpine1, Ccl2 |
| 24h | Upregulate innate  immune-related genes | Lipid and atherosclerosis | 1.67E-19 | 3.60E-17 | Cyba, Xbp1, Stat3, Cxcl1, Cdc42, Nras, Casp8, Ccl2, Cd14, Pycard, Atf4, Hspd1, Jak2, Tnfrsf1a, Icam1, Casp1, Tlr2, Traf2, Myd88, Rela, Abca1, Mapk14, Ikbke, Cxcl2, Akt1, Nfe2l2, Cd36 |
|  |  | Tuberculosis | 1.14E-14 | 1.23E-12 | Fcgr1, Clec4e, Clec7a, Ctss, Casp8, Cd14, Cr1l, Tgfb1, Hspd1, Jak2, Tnfrsf1a, Bcl10, Cebpb, Tlr2, Itgam, Myd88, Rela, H2-Ab1, Itgb2, Mapk14, Akt1 |
|  |  | Legionellosis | 2.69E-14 | 1.93E-12 | Cxcl1, Casp8, Cd14, Pycard, Cr1l, Hspd1, Casp1, Tlr2, Itgam, Myd88, Rela, Itgb2, Naip2, Cxcl2 |
|  |  | TNF signaling pathway | 6.78E-14 | 3.64E-12 | Cxcl1, Bcl3, Casp8, Ccl2, Csf1, Atf4, Tnfrsf1a, Icam1, Edn1, Cebpb, Ripk3, Traf2, Rela, Mapk14, Cxcl2, Akt1, Cxcl10 |
|  |  | C-type lectin receptor signaling pathway | 8.93E-13 | 3.84E-11 | Clec4d, Clec4e, Clec7a, Bcl3, Nras, Casp8, Clec4n, Pycard, Plcg2, Bcl10, Casp1, Ptpn11, Rela, Mapk14, Ikbke, Akt1 |
|  |  | Leishmaniasis | 4.35E-12 | 1.56E-10 | Cyba, Fcgr1, Cr1l, Tgfb1, Jak2, Ptpn6, Tlr2, Itgam, Myd88, Rela, H2-Ab1, Itgb2, Mapk14 |
|  |  | Pertussis | 1.56E-11 | 4.79E-10 | Gnai3, Cd14, Pycard, Casp1, Itgam, Myd88, C1qb, Rela, Itgb2, Mapk14, C1qa, Irf8, C1qc |
|  |  | NOD-like receptor signaling pathway | 2.98E-11 | 8.02E-10 | Cyba, Cxcl1, Ctsb, Casp8, Ccl2, Pycard, Gabarap, Mefv, Casp1, Ripk3, Traf2, Myd88, Gbp2, Rela, Mapk14, Naip2, Ikbke, Cxcl2, Ifnar1 |
|  |  | Chagas disease | 5.21E-11 | 1.24E-09 | Serpine1, Gnai3, Casp8, Ccl2, Tgfb1, Tnfrsf1a, Tlr2, Myd88, C1qb, Rela, Mapk14, C1qa, Akt1, C1qc |
|  |  | NF-kappa B signaling pathway | 6.80E-11 | 1.40E-09 | Trim25, Cxcl1, Cd14, Plcg2, Tnfrsf1a, Icam1, Bcl10, Traf2, Myd88, Rela, Ltbr, Btk, Cxcl2, Il1r1 |
|  | Downregulate innate  immune-related genes | Kaposi sarcoma-associated herpesvirus infection | 5.55E-05 | 3.64E-03 | Tbk1, Tlr3, Mapk8, Traf3, C3, Pik3c3 |
|  |  | RIG-I-like receptor signaling pathway | 6.17E-05 | 3.64E-03 | Tbk1, Mapk8, Cyld, Traf3 |
|  |  | Toll-like receptor signaling pathway | 2.47E-04 | 9.72E-03 | Tbk1, Tlr3, Mapk8, Traf3 |
|  |  | Autophagy - animal | 8.87E-04 | 2.15E-02 | Tbk1, Mapk8, Pik3c3, Tsc1 |
|  |  | Coronavirus disease - COVID-19 | 9.10E-04 | 2.15E-02 | Tbk1, Tlr3, Mapk8, Traf3, C3 |
|  |  | Hepatitis B | 1.56E-03 | 2.75E-02 | Tbk1, Tlr3, Mapk8, Traf3 |
|  |  | Hepatitis C | 1.63E-03 | 2.75E-02 | Tbk1, Tlr3, Traf3, Cd81 |
|  |  | IL-17 signaling pathway | 2.79E-03 | 4.12E-02 | Tbk1, Mapk8, Traf3 |
|  |  | NOD-like receptor signaling pathway | 4.12E-03 | 5.11E-02 | Trpm2, Tbk1, Mapk8, Traf3 |
|  |  | Lipid and atherosclerosis | 4.33E-03 | 5.11E-02 | Tbk1, Vldlr, Mapk8, Traf3 |
| 72h | Upregulate innate  immune-related genes | Lipid and atherosclerosis | 7.24E-26 | 1.60E-23 | Cd14, Pycard, Xbp1, Tnfrsf1a, Casp1, Cdc42, Cyba, Tlr2, Ly96, Mapk3, Rela, Casp7, Icam1, Myd88, Casp8, Nras, Irak4, Stat3, Lbp, Ccl2, Nfe2l2, Tank, Tlr6, Tlr4, Ikbkb, Nfkb1, Mapk14, Ikbke, Abca1, Jak2, Tirap, Nfkbia, Cd36, Hsp90b1, Traf2, Akt1, Cd40, Rac1 |
|  |  | Toll-like receptor signaling pathway | 4.21E-25 | 4.66E-23 | Spp1, Cd14, Tlr2, Ly96, Irf5, Mapk3, Rela, Tlr7, Tlr1, Myd88, Casp8, Irak4, Cxcl10, Lbp, Tlr6, Tlr4, Ikbkb, Nfkb1, Mapk14, Ikbke, Ifnar1, Tirap, Nfkbia, Map3k8, Akt1, Cd40, Stat1, Rac1 |
|  |  | Tuberculosis | 3.23E-24 | 2.38E-22 | Fcgr1, Ifngr1, Cd14, Ctss, H2-Aa, Tgfb1, H2-Ab1, Itgam, Cr1l, Tnfrsf1a, Bcl10, Tlr2, Itgb2, Mapk3, Rela, Cebpb, Tlr1, Myd88, Casp8, Irak4, Lbp, Clec4e, Tlr6, Tlr4, Syk, Nfkb1, Irak2, Mapk14, Jak2, Tirap, C3, Mrc1, Akt1, Stat1 |
|  |  | NOD-like receptor signaling pathway | 1.00E-21 | 5.54E-20 | Pycard, Gabarap, Ctsb, Naip2, Gbp2, P2rx7, Casp1, Cyba, Naip5, Mapk3, Rela, Myd88, Casp8, Sting1, Irak4, Txnip, Ccl2, Tank, Tlr4, Ripk3, Mavs, Ikbkb, Nfkb1, Pstpip1, Mapk14, Ikbke, Ifnar1, Nfkbia, Gbp7, Birc3, Traf2, Prkcd, Stat1, Tnfaip3 |
|  |  | Pertussis | 1.80E-21 | 7.94E-20 | Cd14, Pycard, Gnai3, C1qb, Itgam, Casp1, Ly96, Itgb2, Mapk3, Rela, Casp7, C1qa, Irf8, Myd88, C1qc, Irak4, Tlr4, Serping1, Nfkb1, Mapk14, Gnai2, Tirap, C3 |
|  |  | Toxoplasmosis | 2.79E-21 | 1.03E-19 | Ifngr1, Gnai3, H2-Aa, Tgfb1, H2-Ab1, Tnfrsf1a, Tlr2, Ly96, Pik3cg, Mapk3, Rela, Myd88, Casp8, Irak4, Stat3, Tlr4, Ikbkb, Nfkb1, Mapk14, Gnai2, Jak2, Nfkbia, Birc3, Akt1, Cd40, Stat1 |
|  |  | Leishmaniasis | 3.99E-21 | 1.26E-19 | Fcgr1, Ifngr1, Ptpn6, H2-Aa, Tgfb1, H2-Ab1, Itgam, Cr1l, Cyba, Tlr2, Itgb2, Mapk3, Rela, Myd88, Irak4, Tlr4, Nfkb1, Mapk14, Jak2, Nfkbia, C3, Stat1 |
|  |  | Chagas disease | 8.47E-21 | 2.34E-19 | Ifngr1, Gnai3, C1qb, Tgfb1, Tnfrsf1a, Tlr2, Mapk3, Rela, C1qa, Myd88, Casp8, C1qc, Irak4, Ccl2, Tlr6, Tlr4, Ikbkb, Nfkb1, Cflar, Mapk14, Gnai2, Nfkbia, C3, Serpine1, Akt1 |
|  |  | NF-kappa B signaling pathway | 1.41E-20 | 3.47E-19 | Cd14, Ltbr, Tnfrsf1a, Bcl10, Ly96, Rela, Trim25, Icam1, Myd88, Btk, Plcg2, Irak4, Lbp, Tlr4, Syk, Ikbkb, Nfkb1, Cflar, Tirap, Nfkbia, Relb, Birc3, Traf2, Cd40, Tnfaip3 |
|  |  | Osteoclast differentiation | 2.48E-18 | 5.31E-17 | Fcgr1, Ifngr1, Lcp2, Tgfb1, Csf1r, Csf1, Tnfrsf1a, Cyba, Mapk3, Rela, Tyrobp, Btk, Plcg2, Syk, Ikbkb, Nfkb1, Mapk14, Ifnar1, Sirpa, Nfkbia, Relb, Traf2, Akt1, Stat1, Rac1 |
|  | Downregulate innate  immune-related genes | Th17 cell differentiation | 1.39E-05 | 0.001346943 | Mtor, Ppp3r1, Nfatc2, Mapk8, Rora, Mapk1 |
|  |  | Kaposi sarcoma-associated herpesvirus infection | 1.59E-05 | 0.001346943 | Mtor, Ppp3r1, Nfatc2, Mapk8, Vegfa, Traf3, Mapk1, Pik3c3 |
|  |  | RIG-I-like receptor signaling pathway | 2.74E-05 | 0.001545961 | Pin1, Mapk8, Cyld, Traf3, Tbkbp1 |
|  |  | Dopaminergic synapse | 6.11E-05 | 0.002530005 | Drd2, Camk2a, Mapk8, Arntl, Clock, Gsk3a |
|  |  | Autophagy - animal | 7.49E-05 | 0.002530005 | Mtor, Mapk8, Camkk2, Rheb, Mapk1, Pik3c3 |
|  |  | Lipid and atherosclerosis | 0.000105899 | 0.002586153 | Ppp3r1, Camk2a, Nfatc2, Mapk8, Traf3, Vldlr, Mapk1 |
|  |  | MAPK signaling pathway | 0.000110581 | 0.002586153 | Map4k2, Ppp3r1, Ecsit, Dusp16, Mapk8, Vegfa, Stmn1, Mapk1 |
|  |  | Oxytocin signaling pathway | 0.000122421 | 0.002586153 | Trpm2, Ppp3r1, Camk2a, Nfatc2, Camkk2, Mapk1 |
|  |  | VEGF signaling pathway | 0.000213915 | 0.00401684 | Ppp3r1, Nfatc2, Vegfa, Mapk1 |
|  |  | C-type lectin receptor signaling pathway | 0.00025897 | 0.004376587 | Ppp3r1, Nfatc2, Mapk8, Cyld, Mapk1 |
| 7d | Upregulate innate  immune-related genes | Tuberculosis | 1.41E-18 | 2.89E-16 | Ctss, Itgax, Tlr2, Clec7a, Cd14, Tlr1, H2-Ab1, Cebpb, Mrc1, Stat1, Fcgr1, H2-Aa, Itgb2, Bcl10, Casp8, Mapk14, Itgam, Syk, Tlr4, Ifngr1, Myd88, C3, Irak4, Tlr6, Tnfrsf1a, Cr1l |
|  |  | Leishmaniasis | 2.10E-16 | 2.16E-14 | Ptpn6, Tlr2, H2-Ab1, Stat1, Cyba, Fcgr1, H2-Aa, Itgb2, Mapk14, Itgam, Tlr4, Ifngr1, Myd88, C3, Irak4, Nfkbia, Cr1l |
|  |  | Toll-like receptor signaling pathway | 4.49E-16 | 3.07E-14 | Tlr2, Spp1, Ly96, Cd14, Tlr1, Irf5, Cd86, Stat1, Tlr8, Casp8, Mapk14, Tlr7, Tlr4, Myd88, Irak4, Tlr6, Nfkbia, Map3k8, Cxcl10 |
|  |  | Chagas disease | 1.32E-14 | 6.74E-13 | C1qb, C1qc, Tlr2, C1qa, Ccl2, Casp8, Mapk14, Cflar, Gnai3, Tlr4, Ifngr1, Myd88, C3, Irak4, Tlr6, Tnfrsf1a, Nfkbia, Serpine1 |
|  |  | Pertussis | 2.42E-14 | 9.91E-13 | C1qb, C1qc, C1qa, Ly96, Cd14, Casp1, Irf8, Itgb2, Pycard, Mapk14, Itgam, Gnai3, Tlr4, Myd88, C3, Irak4 |
|  |  | NOD-like receptor signaling pathway | 1.05E-13 | 3.59E-12 | Ctsb, Ccl2, Naip5, Casp1, Nampt, Stat1, Cyba, Naip2, Prkcd, Txnip, Gabarap, Pycard, Gbp2, Pstpip1, Casp8, Mapk14, Tlr4, Myd88, Gbp7, Irak4, Nfkbia, Sting1, P2rx7 |
|  |  | Legionellosis | 2.51E-13 | 7.35E-12 | Tlr2, Cd14, Naip5, Casp1, Naip2, Itgb2, Pycard, Casp8, Itgam, Tlr4, Myd88, C3, Nfkbia, Cr1l |
|  |  | Coronavirus disease - COVID-19 | 3.04E-13 | 7.79E-12 | C1qb, C1qc, Tlr2, C1qa, Ccl2, Casp1, Stat1, C3ar1, Tlr8, Mapk14, Tlr7, Ifih1, Plcg2, Syk, Tlr4, Myd88, C3, Irak4, C5ar1, Tnfrsf1a, Adam17, Nfkbia, Sting1, Cxcl10 |
|  |  | Toxoplasmosis | 6.16E-13 | 1.40E-11 | Tlr2, Ly96, Irgm1, H2-Ab1, Stat1, H2-Aa, Casp8, Mapk14, Gnai3, Tlr4, Ifngr1, Myd88, Irak4, Igtp, Tnfrsf1a, Nfkbia, Pik3cg |
|  |  | Osteoclast differentiation | 7.78E-12 | 1.60E-10 | Tyrobp, Csf1r, Trem2, Sirpa, Stat1, Cyba, Fcgr1, Il1r1, Lcp2, Csf1, Btk, Mapk14, Plcg2, Syk, Ifngr1, Tnfrsf1a, Nfkbia |
|  | Downregulate innate  immune-related genes | RIG-I-like receptor signaling pathway | 0.000119651 | 0.004052338 | Mapk8, Pin1, Tbkbp1, Traf3 |
|  |  | Lipid and atherosclerosis | 0.000120238 | 0.004052338 | Mapk8, Vldlr, Ppp3r1, Mmp9, Traf3, Nfatc2 |
|  |  | Dopaminergic synapse | 0.000124198 | 0.004052338 | Mapk8, Arntl, Clock, Drd2, Gsk3a |
|  |  | Adipocytokine signaling pathway | 0.000126464 | 0.004052338 | Mtor, Mapk8, Ppargc1a, Camkk2 |
|  |  | Kaposi sarcoma-associated herpesvirus infection | 0.000146824 | 0.004052338 | Mtor, Mapk8, Vegfa, Ppp3r1, Traf3, Nfatc2 |
|  |  | Longevity regulating pathway | 0.000316541 | 0.007280444 | Mtor, Ppargc1a, Camkk2, Rheb |
|  |  | Th17 cell differentiation | 0.000549266 | 0.010368687 | Mtor, Mapk8, Ppp3r1, Nfatc2 |
|  |  | MAPK signaling pathway | 0.000634814 | 0.010368687 | Mapk8, Ecsit, Vegfa, Map4k2, Ppp3r1, Stmn1 |
|  |  | Insulin resistance | 0.000678961 | 0.010368687 | Ppargc1b, Mtor, Mapk8, Ppargc1a |
|  |  | TNF signaling pathway | 0.000751354 | 0.010368687 | Mapk8, Mmp9, Traf3, Dab2ip |
| 28d | Upregulate innate  immune-related genes | Pertussis | 2.63E-23 | 5.37E-21 | C1qb, C1qa, C3, Serping1, Casp1, Cd14, Irf8, Itgb2, C1qc, Pycard, Itgam, C2, Gnai3, Ly96, Rela, C1ra, Irf1, Nfkb1, Myd88, Tlr4, Il1a, Irak4, Gnai2 |
|  |  | Tuberculosis | 4.04E-22 | 4.12E-20 | Ctss, C3, Tlr2, Clec7a, Itgax, Fcgr1, Cd14, Tnfrsf1a, Itgb2, Cebpb, Itgam, Bcl10, H2-Ab1, Tlr1, H2-Aa, Tgfb1, Lbp, Cr1l, Casp8, Ifngr1, Rela, Mrc1, Nfkb1, Jak2, Myd88, Tlr4, Il1a, Irak4, Stat1, Syk |
|  |  | Leishmaniasis | 2.16E-21 | 1.47E-19 | C3, Tlr2, Cyba, Fcgr1, Itgb2, Itgam, H2-Ab1, Nfkbia, Ptpn6, H2-Aa, Tgfb1, Cr1l, Ifngr1, Rela, Nfkb1, Jak2, Myd88, Tlr4, Il1a, Irak4, Stat1 |
|  |  | Influenza A | 3.54E-19 | 1.80E-17 | Il33, Casp1, Tnfrsf1a, Icam1, Pycard, H2-Ab1, Nfkbia, Oas1a, H2-Aa, Casp8, Ifngr1, Rela, Ifih1, Trim25, Tlr7, Eif2ak2, Oas1b, Nfkb1, Jak2, Myd88, Tlr4, Il1a, Irak4, Ccl2, Stat1, Tlr3, Cxcl10 |
|  |  | Coronavirus disease - COVID-19 | 4.41E-19 | 1.80E-17 | C1qb, C1qa, C3, Tlr2, Casp1, Plcg2, Tnfrsf1a, C1qc, Nfkbia, Oas1a, C2, Stat3, Rela, Adam17, Ifih1, C5ar1, C1ra, Tlr7, Eif2ak2, Oas1b, C3ar1, Nfkb1, Myd88, Tlr4, Irak4, Ccl2, Stat1, Tlr3, Cxcl10, Sting1, Syk |
|  |  | Toll-like receptor signaling pathway | 7.54E-18 | 2.56E-16 | Tlr2, Spp1, Cd14, Nfkbia, Tlr1, Lbp, Cd86, Casp8, Ly96, Rela, Tlr7, Rac1, Nfkb1, Myd88, Tlr4, Map3k8, Irak4, Stat1, Irf5, Tlr3, Cxcl10 |
|  |  | NF-kappa B signaling pathway | 2.20E-17 | 6.41E-16 | Cd14, Plcg2, Tnfrsf1a, Icam1, Bcl10, Nfkbia, Ltbr, Lbp, Ly96, Rela, Tnfaip3, Trim25, Nfkb1, Myd88, Tlr4, Irak4, Relb, Il1r1, Cflar, Btk, Syk |
|  |  | Toxoplasmosis | 6.05E-17 | 1.54E-15 | Tlr2, Tnfrsf1a, H2-Ab1, Nfkbia, H2-Aa, Tgfb1, Gnai3, Casp8, Ly96, Ifngr1, Stat3, Rela, Pik3cg, Nfkb1, Jak2, Myd88, Tlr4, Irak4, Stat1, Igtp, Gnai2 |
|  |  | Osteoclast differentiation | 1.10E-16 | 2.49E-15 | Csf1r, Tyrobp, Cyba, Fcgr1, Plcg2, Tnfrsf1a, Lcp2, Nfkbia, Tgfb1, Trem2, Sirpa, Ifngr1, Rela, Csf1, Rac1, Nfkb1, Il1a, Relb, Il1r1, Stat1, Btk, Syk |
|  |  | Lipid and atherosclerosis | 1.24E-16 | 2.53E-15 | Tlr2, Nfe2l2, Abca1, Cyba, Casp1, Cd14, Tnfrsf1a, Icam1, Pycard, Nfkbia, Lbp, Cdc42, Abcg1, Casp8, Ly96, Stat3, Rela, Cd36, Rac1, Nfkb1, Jak2, Myd88, Tlr4, Irak4, Ccl2, Hsp90b1, Xbp1 |
|  | Downregulate innate  immune-related genes | Kaposi sarcoma-associated herpesvirus infection | 2.50E-06 | 0.00039813 | Mtor, Traf3, Mapk1, Nfatc2, Ppp3r1, Mapk8, Vegfa |
|  |  | Th17 cell differentiation | 1.06E-05 | 0.000839819 | Mtor, Mapk1, Nfatc2, Ppp3r1, Mapk8 |
|  |  | TNF signaling pathway | 1.58E-05 | 0.000839819 | Traf3, Dab2ip, Mapk1, Mmp9, Mapk8 |
|  |  | VEGF signaling pathway | 2.16E-05 | 0.000858133 | Mapk1, Nfatc2, Ppp3r1, Vegfa |
|  |  | Lipid and atherosclerosis | 2.88E-05 | 0.000915671 | Traf3, Mapk1, Nfatc2, Mmp9, Ppp3r1, Mapk8 |
|  |  | RIG-I-like receptor signaling pathway | 4.56E-05 | 0.001207375 | Traf3, Pin1, Tbkbp1, Mapk8 |
|  |  | Pancreatic cancer | 6.30E-05 | 0.001431015 | Mtor, Mapk1, Mapk8, Vegfa |
|  |  | Hepatitis B | 9.22E-05 | 0.001778002 | Traf3, Mapk1, Nfatc2, Mmp9, Mapk8 |
|  |  | Th1 and Th2 cell differentiation | 0.000111824 | 0.001778002 | Mapk1, Nfatc2, Ppp3r1, Mapk8 |
|  |  | PD-L1 expression and PD-1 checkpoint pathway in cancer | 0.000111824 | 0.001778002 | Mtor, Mapk1, Nfatc2, Ppp3r1 |

**SCI: Spinal cord injury; h: hours; d: days; KEGG: Kyoto Encyclopedia of Genes and Genomes.**

**Supplemental Table 4 Annotation of hub innate immune-related genes**

| Gene  Item | Full name | Function |
| --- | --- | --- |
| Ccl2 | Chemokine (C-C motif) ligand 2 | Cause a strong chemotactic response and mobilization of intracellular calcium ions.  Plays an important role in mediating peripheral nerve injury-induced neuropathic pain.  Exhibits a chemotactic activity for monocytes and basophils but not neutrophils or eosinophils. |
| Myd88 | Myeloid differentiation primary response gene 88 | Participates in the Toll-like receptor and IL-1 receptor signaling pathway in the innate immune response.  Leads to NF-kappa-B activation, cytokine secretion and the inflammatory response.  Participates in IL-18-mediated signaling pathway.  Acts as a negative regulator of activation by IL-1 or lipopolysaccharide.  Maintains gut homeostasis and controls the expression of the antimicrobial lectin REG3G in the small intestine.  Mediates leukocyte recruitment at the inflammatory site. |
| Stat3 | Signal transducer and activator of transcription 3 | Mediates cellular responses to interleukins, KITLG/SCF, LEP and other growth factors.  Acts as a regulator of inflammatory response by regulating differentiation of naive CD4+ T-cells into T-helper Th17 or regulatory T-cells.  Plays an apoptotic role by transactivating BIRC5 expression under LEP activation.  Cytoplasmic STAT3 represses macro autophagy by inhibiting EIF2AK2/PKR  Activity. |
| Cxcl10 | Chemokine (C-X-C motif) ligand 10 | Participates in some processes such as chemotaxis, differentiation, and activation of peripheral immune cells, regulation of cell growth, apoptosis and modulation of angiostatin effects.  Plays an important role in during viral infections by stimulating the activation and migration of immune cells to the infected sites.  Activates and directs microglia to the brain injury lesion site and affects neuronal reorganization. |
| Mapk14 | Mitogen-activated protein kinase 14 | Plays an important role in the cascades of cellular responses evoked by extracellular stimuli.  Plays an important role in the rapid induction of immediate-early genes in response to stress or mitogenic stimuli.  Plays an important role in protein turnover.  Regulates the endocytosis of membrane receptors.  Inhibits the lysosomal degradation pathway of autophagy.  Plays an essential role in developmental and stress-induced erythropoiesis. |
| Stat1 | Signal transducer and activator of transcription 1 | Mediates cellular responses to interferons, cytokine KITLG/SCF and other cytokines and other growth factors.  Mediates cellular responses to activated FGFR1, FGFR2, FGFR3 and FGFR4.  Induces a cellular antiviral state. |
| Tlr2 | toll-like receptor 2 | Leading to NF-kappa-B activation, cytokine secretion and the inflammatory response.  Promotes apoptosis in response to lipoproteins. |
| Itgam | integrin alpha M | Participates in various adhesive interactions of monocytes, macrophages and granulocytes.  Regulates neutrophil migration and activation.  May regulate phagocytosis-induced apoptosis in extravasated neutrophils.  May play a role in mast cell development.  Promotes the neuronal apoptosis that occurs during brain development. |
| Mapk8 | mitogen-activated protein kinase 8 | Participates in various processes such as cell proliferation, differentiation, migration, transformation and programmed cell death.  Participates in the polarized differentiation of T-helper cells into Th1 cells.  Participates in the activation of autophagy.  Controls the axon elongation of cortical neurons. |
